# Approaches to analyzing binary data for large-scale A/B testing

**DOI:** 10.1016/j.conctc.2023.101091

**Published:** 2023-02-16

**Authors:** Wenru Zhou, Miranda Kroehl, Maxene Meier, Alexander Kaizer

**Affiliations:** a Department of Biostatistics & Informatics, University of Colorado, United States; b Digital Platforms Organization Charter Communications, United States; c Charter Communications, United States

**Keywords:** A/B testing, Interim monitoring, O'Brien-Fleming boundaries, Academic-industry partnership

## Abstract

An industry-academic collaboration was established to evaluate the choice of statistical test and study design for A/B testing in larger-scale industry experiments. Specifically, the standard approach at the industry partner was to apply a *t*-test for all outcomes, both continuous and binary, and to apply naïve interim monitoring strategies that had not evaluated the potential implications on operating characteristics such as power and type I error rates. Although many papers have summarized the robustness of the *t*-test, its performance for the A/B testing context of large-scale proportion data, with or without interim analyses, is needed. Investigating the effect of interim analyses on the robustness of the *t*-test is important, because interim analyses rely on a fraction of the total sample size and one should ensure that desired properties are maintained when a *t*-test is implemented not just at the end of the study, but for making interim decisions. Through simulation studies, the performance of the *t*-test, Chi-squared test, and Chi-squared test with Yate's correction when applied to binary outcomes data is evaluated. Further, interim monitoring through a naïve approach with no correction for multiple testing versus the O'Brien-Fleming boundary are considered in designs that allow early termination for futility, difference, or both. Results indicate that the *t*-test achieves similar power and type I error rates for binary outcomes data with the large sample sizes used in industrial A/B tests with and without interim monitoring, and naïve interim monitoring without corrections leads to poorly performing studies.

## 1 Introduction

“A/B tests” are randomized experiments where two or more variants are compared and represent a context-specific use case of existing randomized controlled trials (RCT) designs and evaluation approaches. For example, experimentation is a key strategy used within Charter Communications' Product & Technology organization to inform how product modification impacts product function or customer experience so they can identify the optimal features for a given product. Certain product decisions rely on the results of an A/B test thus necessitating timely answers to a study, and teams will monitor their experiment's data on an on-going basis in order to facilitate rapid data-driven decision making.

From the industry perspective, it is imperative to standardize design, implementation, and analysis techniques in order to meet the needs of an online experimentation program at scale. Arguably the most common hypothesis test taught in statistical courses is the two-sample *t*-test [15], which is used to compare the means between two groups of variables arising from continuous distributions. Another common method is the Chi-square test ($\chi^{2}$), used to compare the two independent binomial populations. Based on the theoretical assumptions for each test, the *t*-test would be applied to data arising from a continuous distribution, whereas the $\chi^{2}$ test, or Chi-square test with Yate's correction ($\chi_{c}^{2}$), would be used to test binomial outcomes. However, given the desire to standardize as much as possible, the ideal test based on statistical theory is not always implemented. The *t*-test is a popular choice to compare both group means and group proportions due to its' simplicity in implementation and automation, as well as its' ease for interpretation. Furthermore, when results can be made readily available through automated jobs, it feels reasonable as a product team member to monitor your data on a regular basis and end a study as quickly as possible when the data suggest a clear winner.

From the academic perspective, the choice of statistical test is based on the underlying data and accompanying model assumptions. For example, in an experiment with a binary outcome the chi-squared test is often used to compare two independent proportions when the sample size is not too small [1]. When sample size is small, Fisher's exact test or $\chi^{2}$ test with Yate's correction would be used [2]. However, there has been work that shows that the Chi-square test with Yate's correction [3] and Fisher's exact test [4] overcorrect the p-values, resulting in diminished statistical power at the expense of an overly conservative type I error rate. These approaches are recommended over the *t*-test, because the binary data is not continuous. However, asymptotic theory indicates that the difference between two proportions is approximately normal distributed as the sample size increases.

From the traditional biomedical randomized trial literature, there is extensive prior research on interim monitoring approaches [5]. summarized methodology for controlling type I errors so that an experiment can be stopped early if there is strong evidence of some difference and/or futility during an interim analysis for medical clinical trials. One approach is to use ad hoc rules to attempt to ensure that study operating characteristics (e.g., power and type I error rates) are maintained through the implementation of interim analyses [6]. Alternatively, group sequential tests incorporate increasingly flexible stopping boundaries and have been proposed by Refs. [[7], [8], [9], [10], [16]], have to be incorporated into clinical research and maintain the study operating characteristics while incorporating interim evaluations of the data to determine if a study should stop early for futility (i.e., not detecting an effect), difference (i.e., finding superiority or inferiority), or both. More details are in the appendix. However, because the stopping boundaries for interim analyses require careful design for each study, it is not feasible for companies to run hundreds of ongoing studies due to time and resource constraints. Inappropriate designs may lead to inflated type I error [13] rates where a type I error for a company means that this company will implement changes that make no difference from the existing standard but may lose both money and customers. Further, investigating the effect of interim analyses on the robustness of the *t*-test is important, because interim analyses rely on a fraction of the total sample size and one should ensure that desired properties are maintained when a *t*-test is implemented not just at the end of the study, but for making interim decisions.

There are differences in the approach and motivation by industry and academic perspectives. In this paper we outline an academic-industry partnership that seeks to address the questions raised by an industry partner through possible solutions and methods provided by the academic partner. In the “Data and Methods” section we detail the collaborative process and the use of simulation studies to evaluate trial operating characteristics across a range of scenarios. The "Results” present the simulation studies across a range of sample sizes, effect sizes, and stopping boundaries with a varying number of interim looks. We conclude with a brief discussion of the collaboration and implications of the results for future A/B testing.

## 2 Motivating example

Charter Communications, Inc. is a broadband connectivity company and cable operator through its Spectrum brand [11]. Spectrum Business is a division of Charter Communications providing superior Internet, phone, and TV services to small businesses [12]. Several million Spectrum customers use the one-time-payment (OTP) feature each month. The standard OTP flow required customers to walk through a four-step process, and multiple steps presented multiple opportunities for failure. While OTP failure rates were low, the Billing team hypothesized they could improve the customer experience and reduce failures with a new “quick pay” flow that would reduce the number of steps by half. An A/B test with 1:1 randomization and binary outcome of an OTP failure was designed to evaluate the impact of this new payment flow using the company's standardized design process, which set the experiment duration for a month. Given the number of customers who could benefit from this improved flow should it prove as successful as anticipated, establishing guidelines for robust interim monitoring for industry A/B tests would provide significant value to the company.

## 3 Data and Methods

### 3.1 The collaboration

An industry-academic collaboration was established between stakeholders at Charter Communications and a faculty-student pair at the University of Colorado. While the client has a PhD in Biostatistics, she oversees an analytic team with quantitative, but heterogeneous, backgrounds that did not necessarily include training on complex study design. Further, other corporate stakeholders have varying levels of statistical training and desire to avoid overly complicated approaches when possible to prioritize those that can be easily scaled up and automated across a series of A/B tests.

Given these considerations, the collaboration identified two questions of interest. First, is the use of the *t*-test appropriate even though the outcomes in many experiments are binary? Especially, since the *t*-test is a common method in industry, does it provide similar power and type I error rates to a chi-squared test? Second, are rigorous boundaries, such as O'Brien-Fleming boundaries, necessary, or are naïve looks at the data without any adjustment of the alpha level appropriate for A/B testing? The chief challenge was identifying methods that were considered approachable to be of interest to the broad audience of stakeholders at the industry client, while still incorporating the extensive prior research conducted in the academic clinical trial research community for interim monitoring.

### 3.2 Simulation studies

A common cross-sectional A/B scenario is simulated to compare the proportion responding in the “A” variant ($\theta_{A}$) and the “B” variant ($\theta_{B}$) under the null hypothesis of no difference versus the alternative hypothesis that there is some difference in variants. A two-sample two-sided *t*-test is used as our primary benchmark based on the existing industry approach and is compared to the chi-squared test $\left(\chi^{2}\right)$ test with and without continuity correction. For interim monitoring approaches, a naïve method without any adjustment in alpha level is compared to the O'Brien Fleming stopping boundary. Each interim monitoring approach is evaluated under three stopping strategies: futility only, difference only, or both. A final approach is a fixed sample design without interim monitoring. The number of interim looks at the data are examined in simulations with 1-, 3-, and 19-interim analyses for a maximum number of 2-, 4-, 20-looks at the data, respectively.

Assuming a constant response in variant “A” of $\theta_{A}$ = is $\theta_{B}$ : 0.589 (large effect), 0.528 (moderate effect), 0.509 (small effect), 0.504 (tiny effect), and 0.500 (no effect). These effects were driven to reflect A/B tests that would enroll 500, 5000, 50,000, or 250,000 per variant to detect the decreasing effect sizes, respectively, in a fixed sample design without interim monitoring. The maximum sample size needed to detect a given effect size for the corresponding stopping boundaries for the O'Brien Fleming interim monitoring method were calculated using PROC SEQDESIGN in SAS (Cary, North Carolina). The naïve interim monitoring approach is implemented assuming the same maximum sample size calculated for the fixed sample design but with arbitrary thresholds set by the collaborator for what they would use in practice without application of existing group sequential designs such as O'Brien-Fleming boundaries: p < 0.025 and p > 0.975 were used to declare significance while p-values between 0.25 and 0.75 were used to terminate for futility in interim analysis.

A total of 10,000 simulated studies were conducted in R (Vienna, Austria) for each effect size. Equal accrual was assumed between all interim analyses. The simulation design and evaluation are summarized in Table 1. Sample size calculations assumed α = 0.05 and 80% power.

**Table 1:** Summary of simulation scenarios and assumptions.

Simulation scenarios				
Fixed study sample size per arm	500	5000	50000	250000
θ_B_	0.589	0.528	0.509	0.504
θ_A_	0.5			
Boundaries	Naïve and O'Brien Fleming interim monitoring method with stopping for futility, difference, and both; Fixed sample design			
Statistical tests	Two-sided T test Chi-sq test without Yates' correction Chi-sq test with Yates' correction			
Number of total analyses	2-, 4-, 20-total analyses			
Type I error rate	5%			
Power	80%			

## 4 Results

The power and type I error for all approaches and tests are shown by the different effect sizes in Table 2, Table 3, Table 4, Table 5. In general, the *t*-test and chi-squared test *without* correction perform similarly across all examined effect sizes with minimal differences in performance decreasing as the sample sizes increase until identical results are observed with the largest sample size (Table 5). The chi-squared test *with* correction is more conservative than the other two tests with lower powers and type I error rates. The shrinkage of differences as the sample size increases reflects the expected asymptotic properties of the chi-squared tests as the sample size increases. These trends are observed across all analysis strategies and varying numbers of interim looks, where an increasing sample size leads to increasingly similar results across all three analytic strategies.

**Table 2:** Power and type I error rates from 10,000 simulated A/B tests to detect a large effect size (fixed sample size of 500 per group).

Number of total analyses	Boundaries		Power for large effect size			Type I error for large effect size		
	Interim Monitoring Boundaries	Stopping rules	T-test	Chi-sq test	Chi-sq test with Yate's correction	T-test	Chi-sq test	Chi-sq test with Yate's correction
1	Fixed sample size	No Early Stop	0.8098	0.8098	0.7933	0.0543	0.0543	0.0468
2	O'Brien Fleming	Early Stop for Futility	0.8048	0.8048	0.7883	0.0552	0.0552	0.0484
	O'Brien Fleming	Early Stop for Both	***0.8029***	***0.8038***	0.7819	0.0488	0.0488	0.0432
	O'Brien Fleming	Early Stop for Difference	0.8006	0.8006	0.7801	0.0473	0.0473	0.0402
	Naive	Early Stop for Futility	0.780178	0.780178	0.759676	0.0496	0.0496	0.042
	Naive	Early Stop for Both	0.8003	0.8003	0.7783	0.0852	0.0852	0.0698
	Naive	Early Stop for Difference	0.8299	0.8299	0.8119	0.0899	0.0899	0.0746
4	O'Brien Fleming	Early Stop for Futility	0.7983	0.7983	0.7766	0.0499	0.0499	0.0435
	O'Brien Fleming	Early Stop for Both	0.8040	0.8040	0.7844	0.0511	0.0511	0.0428
	O'Brien Fleming	Early Stop for Difference	***0.8017***	***0.8024***	0.7816	0.0470	0.0470	0.0406
	Naive	Early Stop for Futility	0.656966	0.656966	0.613861	0.0365	0.0365	0.0295
	Naive	Early Stop for Both	0.6921	0.6921	0.6451	0.1047	0.1047	0.0836
	Naive	Early Stop for Difference	0.85	0.85	0.8297	0.1312	0.1312	0.1096
20	O'Brien Fleming	Early Stop for Futility	***0.8101***	***0.8104***	0.7933	***0.0516***	***0.0517***	0.0439
	O'Brien Fleming	Early Stop for Both	0.8109	0.8109	0.7912	0.0535	0.0535	0.0461
	O'Brien Fleming	Early Stop for Difference	0.7981	0.7981	0.7795	0.0500	0.0500	0.0438
	Naive	Early Stop for Futility	**0.289429**	**0.289529**	0.214721	0.0129	0.0129	0.0079
	Naive	Early Stop for Both	**0.3441**	**0.3443**	0.2491	**0.1351**	**0.1356**	0.0797
	Naive	Early Stop for Difference	**0.8902**	**0.8904**	0.8677	**0.2652**	**0.2669**	0.2047

Note: The power and type I error are bolded if they are different between *t*-test and chi-squared test without correction.

**Table 3:** Power and type I error rates from 10,000 simulated A/B tests to detect a moderate effect size (fixed sample size of 5000 per group).

Number of total analyses	Boundaries		Power for moderate effect size			Type I error for moderate effect size		
	Interim Monitoring Boundaries	Stopping rules	T-test	Chi-sq test	Chi-sq test with Yate's correction	T-test	Chi-sq test	Chi-sq test with Yate's correction
1	Fixed sample size	No Early Stop	***0.8019***	***0.8020***	0.7968	***0.0512***	***0.0518***	0.0491
2	O'Brien Fleming	Early Stop for Futility	0.8013	0.8013	0.7973	0.0527	0.0527	0.0502
	O'Brien Fleming	Early Stop for Both	0.8014	0.8014	0.7949	0.0523	0.0523	0.0493
	O'Brien Fleming	Early Stop for Difference	0.7989	0.7989	0.7927	0.0493	0.0493	0.0468
	Naive	Early Stop for Futility	0.7739	0.774	0.7669	**0.0467**	**0.0472**	0.0446
	Naive	Early Stop for Both	0.7924	0.7924	0.7856	0.08	0.0803	0.0763
	Naive	Early Stop for Difference	0.8204	0.8204	0.8155	**0.0845**	**0.0849**	0.0808
4	O'Brien Fleming	Early Stop for Futility	0.7993	0.7993	0.7927	0.0531	0.0531	0.0509
	O'Brien Fleming	Early Stop for Both	0.7958	0.7958	0.7905	0.0500	0.0500	0.0483
	O'Brien Fleming	Early Stop for Difference	0.7990	0.7990	0.7935	0.0502	0.0502	0.0469
	Naive	Early Stop for Futility	0.6595	0.6596	0.6473	0.0353	0.0356	0.0328
	Naive	Early Stop for Both	**0.6924**	**0.6925**	0.6801	**0.1038**	**0.1053**	0.0986
	Naive	Early Stop for Difference	**0.8413**	**0.8414**	0.8363	**0.127**	**0.1288**	0.1218
20	O'Brien Fleming	Early Stop for Futility	0.8029	0.8029	0.7979	0.0509	0.0509	0.0483
	O'Brien Fleming	Early Stop for Both	0.8017	0.8017	0.7955	0.0511	0.0511	0.0486
	O'Brien Fleming	Early Stop for Difference	0.7972	0.7972	0.7912	0.0491	0.0491	0.0468
	Naive	Early Stop for Futility	0.3048	0.3048	0.2783	**0.0126**	**0.0128**	0.0114
	Naive	Early Stop for Both	0.354	0.354	0.3223	0.1281	0.1282	0.1097
	Naive	Early Stop for Difference	0.8841	0.8841	0.8789	**0.2586**	**0.2594**	0.2401

Note: The power and type I error are bolded if they are different between *t*-test and chi-squared test without correction.

**Table 4:** Power and type I error rates from 10,000 simulated A/B tests to detect a small effect size (fixed sample size of 50000 per group).

Number of total analyses	Boundaries		Power for small effect size			Type I error for small effect size		
	Interim Monitoring Boundaries	Stopping rules	T-test	Chi-sq test	Chi-sq test with Yate's correction	T-test	Chi-sq test	Chi-sq test with Yate's correction
1	Fixed sample size	No Early Stop	0.7958	0.7958	0.7933	0.0470	0.047	0.0460
2	O'Brien Fleming	Early Stop for Futility	0.7964	0.7964	0.7943	0.0496	0.0496	0.0488
	O'Brien Fleming	Early Stop for Both	0.7986	0.7986	0.7964	0.0485	0.0485	0.0480
	O'Brien Fleming	Early Stop for Difference	0.7945	0.7945	0.7931	0.0464	0.0464	0.0458
	Naive	Early Stop for Futility	0.7663	0.7663	0.7636	0.0418	0.0418	0.0409
	Naive	Early Stop for Both	0.7882	0.7882	0.7853	0.076	0.076	0.0743
	Naive	Early Stop for Difference	0.8177	0.8177	0.815	0.0812	0.0812	0.0794
4	O'Brien Fleming	Early Stop for Futility	0.7948	0.7948	0.7927	0.0500	0.0500	0.0492
	O'Brien Fleming	Early Stop for Both	0.7955	0.7955	0.7929	0.0493	0.0493	0.0482
	O'Brien Fleming	Early Stop for Difference	0.7976	0.7976	0.7958	0.0490	0.0490	0.0481
	Naive	Early Stop for Futility	0.6519	0.6519	0.6476	0.0317	0.0317	0.0313
	Naive	Early Stop for Both	0.6887	0.6887	0.6841	0.1001	0.1001	0.0972
	Naive	Early Stop for Difference	0.8386	0.8386	0.8361	0.1227	0.1227	0.1192
20	O'Brien Fleming	Early Stop for Futility	0.7968	0.7968	0.7953	0.0495	0.0495	0.0485
	O'Brien Fleming	Early Stop for Both	0.7972	0.7972	0.7954	0.0482	0.0482	0.0475
	O'Brien Fleming	Early Stop for Difference	0.7967	0.7967	0.7938	0.0488	0.0488	0.0474
	Naive	Early Stop for Futility	0.3101	0.3101	0.3026	0.0128	0.0128	0.0124
	Naive	Early Stop for Both	0.359	0.359	0.3504	0.126	0.126	0.1207
	Naive	Early Stop for Difference	0.8804	0.8804	0.8781	0.2457	0.2457	0.2396

Note: The power and type I error are bolded if they are different between *t*-test and chi-squared test without correction.

**Table 5:** Power and type I error rates from 10,000 simulated A/B tests to detect a tiny effect size (fixed sample size of 250000 per group).

Number of total analyses	Boundaries		Power for tiny effect size			Type I error tiny effect size		
	Interim Monitoring Boundaries	Stopping rules	T-test	Chi-sq test	Chi-sq test with Yate's correction	T-test	Chi-sq test	Chi-sq test with Yate's correction
1	Fixed sample size	No Early Stop	0.7972	0.7972	0.7962	0.0446	0.0446	0.0442
2	O'Brien Fleming	Early Stop for Futility	0.8016	0.8016	0.8005	0.0471	0.0471	0.0466
	O'Brien Fleming	Early Stop for Both	0.8027	0.8027	0.8021	0.0455	0.0455	0.0452
	O'Brien Fleming	Early Stop for Difference	0.7975	0.7975	0.7966	0.0454	0.0454	0.0450
	Naive	Early Stop for Futility	0.766	0.766	0.7647	0.0405	0.0405	0.0402
	Naive	Early Stop for Both	0.7875	0.7875	0.7863	0.0723	0.0723	0.0718
	Naive	Early Stop for Difference	0.8187	0.8187	0.8178	0.0764	0.0764	0.0758
4	O'Brien Fleming	Early Stop for Futility	0.8023	0.8023	0.8011	0.0473	0.0473	0.0467
	O'Brien Fleming	Early Stop for Both	0.7995	0.7995	0.7985	0.0483	0.0483	0.0476
	O'Brien Fleming	Early Stop for Difference	0.7964	0.7964	0.7954	0.0459	0.0459	0.0454
	Naive	Early Stop for Futility	0.6527	0.6527	0.6507	0.0312	0.0312	0.0309
	Naive	Early Stop for Both	0.6907	0.6907	0.6885	0.0996	0.0996	0.0982
	Naive	Early Stop for Difference	0.8417	0.8417	0.8405	0.1209	0.1209	0.1196
20	O'Brien Fleming	Early Stop for Futility	0.7976	0.7976	0.7965	0.0491	0.0491	0.0487
	O'Brien Fleming	Early Stop for Both	0.8048	0.8048	0.8037	0.0485	0.0485	0.0482
	O'Brien Fleming	Early Stop for Difference	0.7993	0.7993	0.7984	0.0473	0.0473	0.0471
	Naive	Early Stop for Futility	0.3068	0.3068	0.3033	0.0134	0.0134	0.0127
	Naive	Early Stop for Both	0.3562	0.3562	0.3517	0.1269	0.1269	0.123
	Naive	Early Stop for Difference	0.8831	0.8831	0.8816	0.2436	0.2436	0.2407

When considering a naïve approach to interim monitoring with early stopping for futility, the type I error is overly conservative by being less than the target of 0.05, and correspondingly the power is much lower than its target of 0.8. For naive peeking approach with early stopping for difference, the power is slightly above 0.8; but, the type I error is severally inflated above 0.08 for 2-total looks and 0.l1 for 4-total looks. For naïve peeking approach with early stop for both, neither of power nor type I error is acceptable except for the power of design with 2-total look and 500 per arm. As the total number of analyses increases, the impact also increases with operating characteristics increasingly far off their targets of 0.05 for type I error and 0.8 for power.

In contrast, the O'Brien-Fleming stopping boundaries do maintain the approximately desired operating characteristics across all scenarios and the increasing number of interim analyses. This is because the stopping boundaries are chosen based on the targeted type I error and power. While this implies a more complex series of boundaries than the naïve approach, it better maintains the study integrity. The trade-off to using these O'Brien-Fleming boundaries is that the maximum sample size does increase relative to the fixed sample or naïve approaches. For example, with 1-interim look the maximum sample size increases from 500 to 502 (a 0.4% increase), 517 (a 3.4% increase), and 519 (a 3.8% increase) when stopping for only a difference, only futility, or both, respectively. With 19-interim looks, the maximum sample sizes increase to 524 (a 4.8% increase), 530 (a 6.0% increase), and 546 (a 9.2% increase) when stopping for only a difference, only futility, or both, respectively.

## 5 Discussion

The intention of this article was to present the industry-academic collaboration to evaluate the acceptability of applying the two sample *t*-test with unequal variance when comparing two proportions on large scale A/B testing environments and to examine the study type I error and power when we are evaluating the data multiple times with and without formal interim monitoring boundaries. A simulation study was implemented across a range of scenarios to determine if general guidance of the statistical tests could be given for A/B experimentation. We found that the *t*-test is feasible for comparing two independent proportions with large sample sizes ( ≥ 500 per variant) since it provides very similar results with chi-squared test. This suggests the sample sizes explored to reflect the common A/B testing contexts at Charter Communication achieved adequate convergence towards the limiting distributions and result in generally similar conclusions. These results may contrast from traditional clinical settings, where studies are often based on much smaller sample sizes and the differences in choice of test may have much larger impacts on the type I error rate and power.

With respect to choice of interim monitoring boundary, our simulation results indicate that naïve approaches lead to suboptimal performance with type I error rates and power that do not meet the desired level. In certain circumstances, such as stopping for both futility or a difference, one may end up with *both* inflated type I error rates and an underpowered study, especially as the number of interim looks increases. Conversely, using an approach based on maintaining a desired type I error rate that accounts for the number of looks, such as the O'Brien Fleming boundaries, leads to results near the targeted power and type I error rates. The trade-off is an increase in the maximum sample size if the study does not terminate early.

This research has limitations worth discussing, with room for further research. We performed statistical tests assuming all individuals are independent. However, in practice, the participants may not be independent. It is possible that customers have multiple accounts with Charter, and family members or friends might have strongly correlated outcomes. In addition, we only conducted the simulation for an adequately powered design and the O'Brien-Fleming error spending function. It is worth extending the simulation to over- and under-powered design and additional error spending functions to determine the extent to which automation can take place without having each study designed specifically for the expected effect size. Lastly, for all three statistical tests, the sample size calculations were all based on the $\chi^{2}$ test, since it was the only choice for binary data in PROC SEQDESIGN. However, this limitation did not have a big impact on the type I error and power in our simulation results for the *t*-test.

With respect to the collaborative process, these results can be generalized to other A/B tests with large sample sizes (>500 per group) to suggest that the *t*-test is appropriate for binary data. This may facilitate greater automation, especially if A/B tests over time use a mixture of binary and continuous outcomes. The results were effectively communicated to the industry client through presentations and a report to communicate the statistical methodology and results aimed at a non-statistical audience. Additional technical details were provided through small group meetings and a technical appendix. For future projects, we will spend more time listening to the unique needs of an industry setting and on bridging the difference of terminology and study concerns between academic and industry settings.

## Declaration of competing interest

The authors declare that they have no known competing financial interests or personal relationships that could have appeared to influence the work reported in this paper.

## Data Availability

Data will be made available on request.
