# Supplementary material for: Approaches to analyzing binary data for large-scale A/B testing
Source: Contemp Clin Trials Commun. 2023 Feb 16;32:101091. doi: 10.1016/j.conctc.2023.101091 (PMC9982610; doi:10.1016/j.conctc.2023.101091)

# **Appendix**

## Error Spending Functions

It has been demonstrated previously, both via asymptotic properties and simulation studies, that multiple testing without any correction leads to an inflated type I error rate. Error spending functions proposed by Demets and Lan (1994) are functions that govern the cumulative type I error that is "spent" at each interim analysis during a sequential testing. By using error spending functions to carefully design a study, one can do multiple testing without inflating the type I error, since the overall type I error rate is still controlled at the end of the study. Common error spending functions include Pocock boundaries, whose mathematical function is αln[1+(e−1)t*], and O'Brien-Fleming boundaries, whose function is 2−2Φ(Z1−α/2/√ t*).

## Expected Sample Size Figures

The following figures summarize the expected sample size across all simulation settings with reference to the fixed sample design. 2-total looks is presented on pages 2-11, 4-total looks is on pages 12-21, and 20-total looks is on pages 22-31.

## 2-Total Looks

##
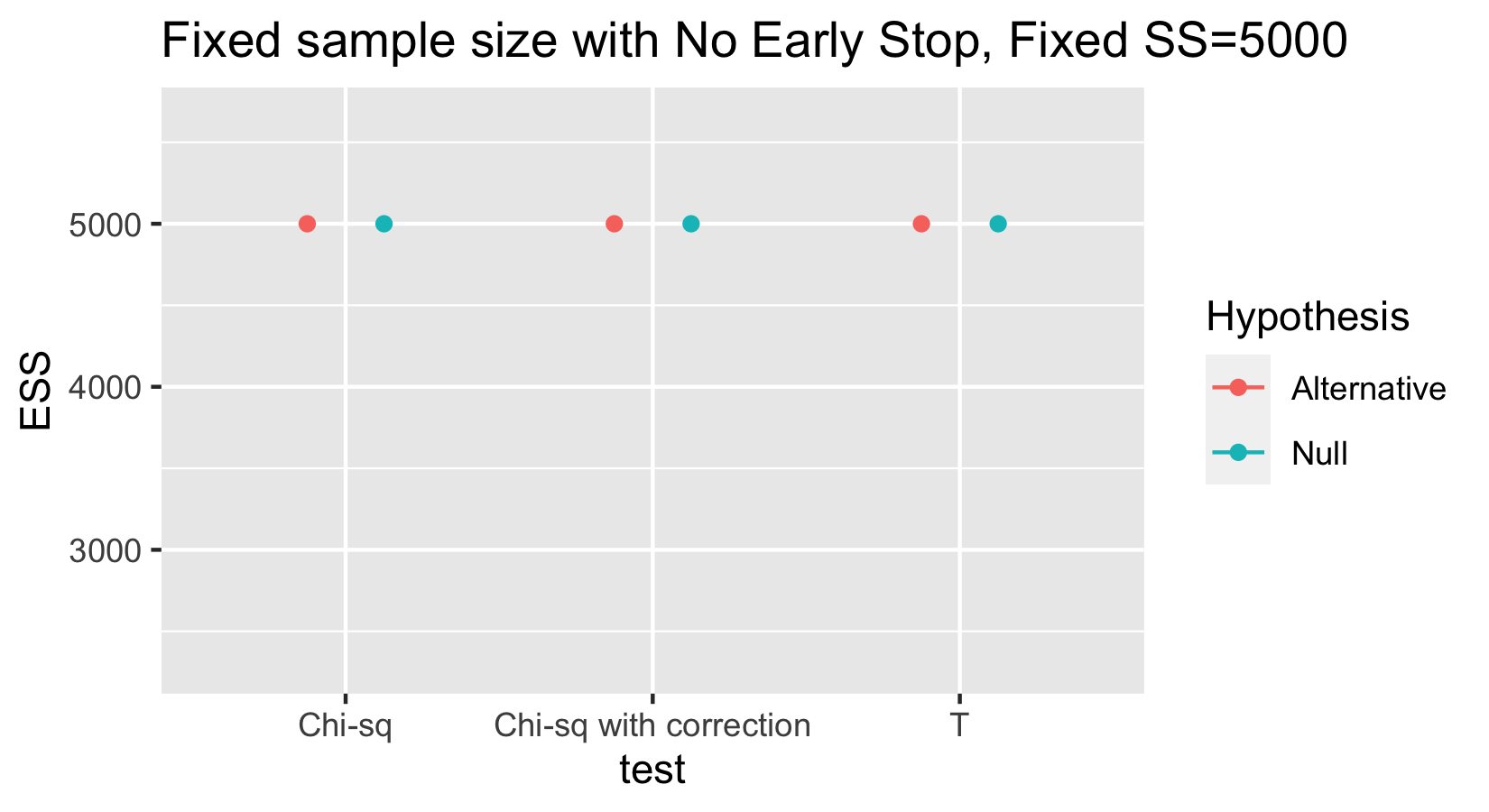

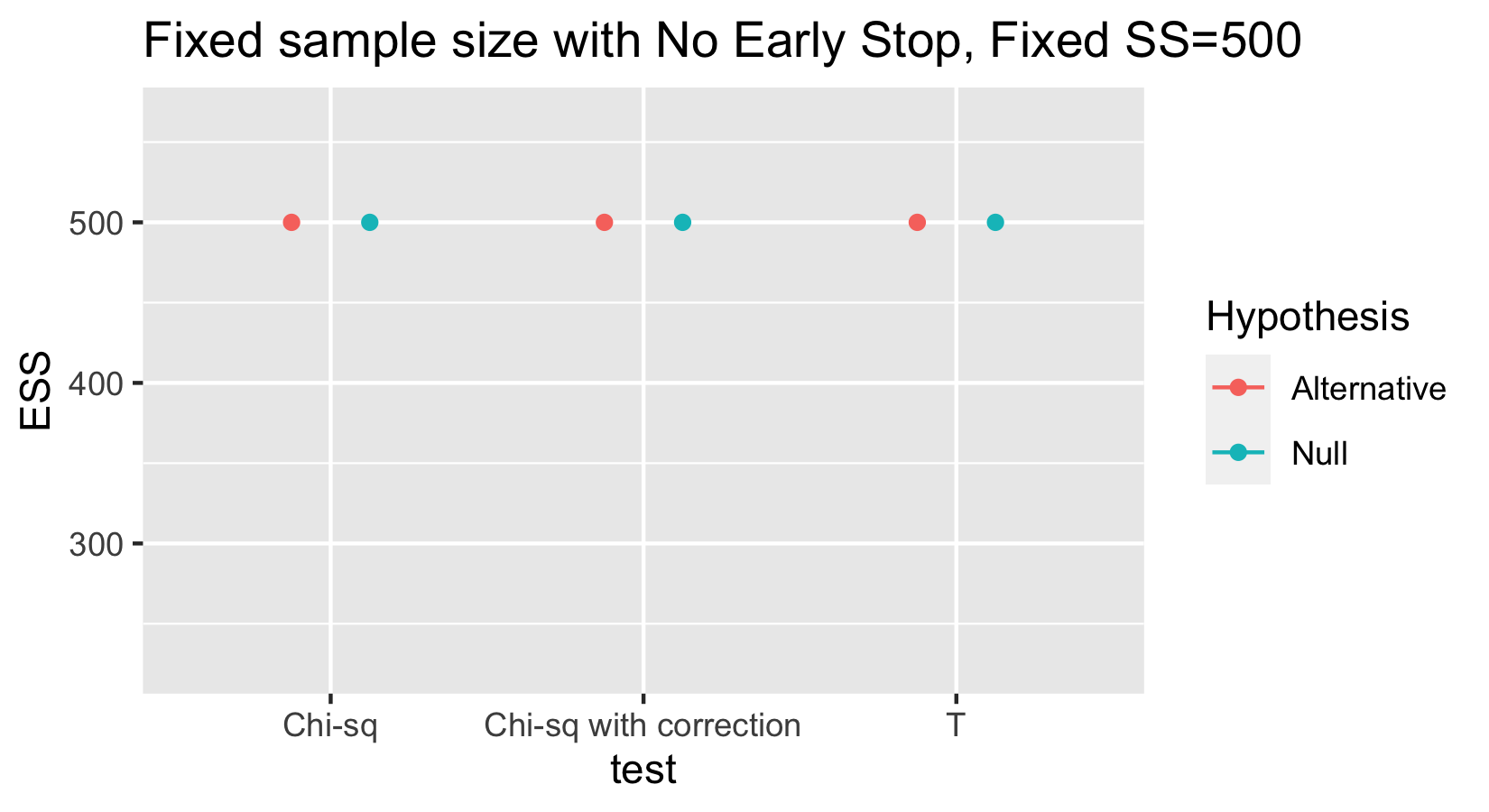

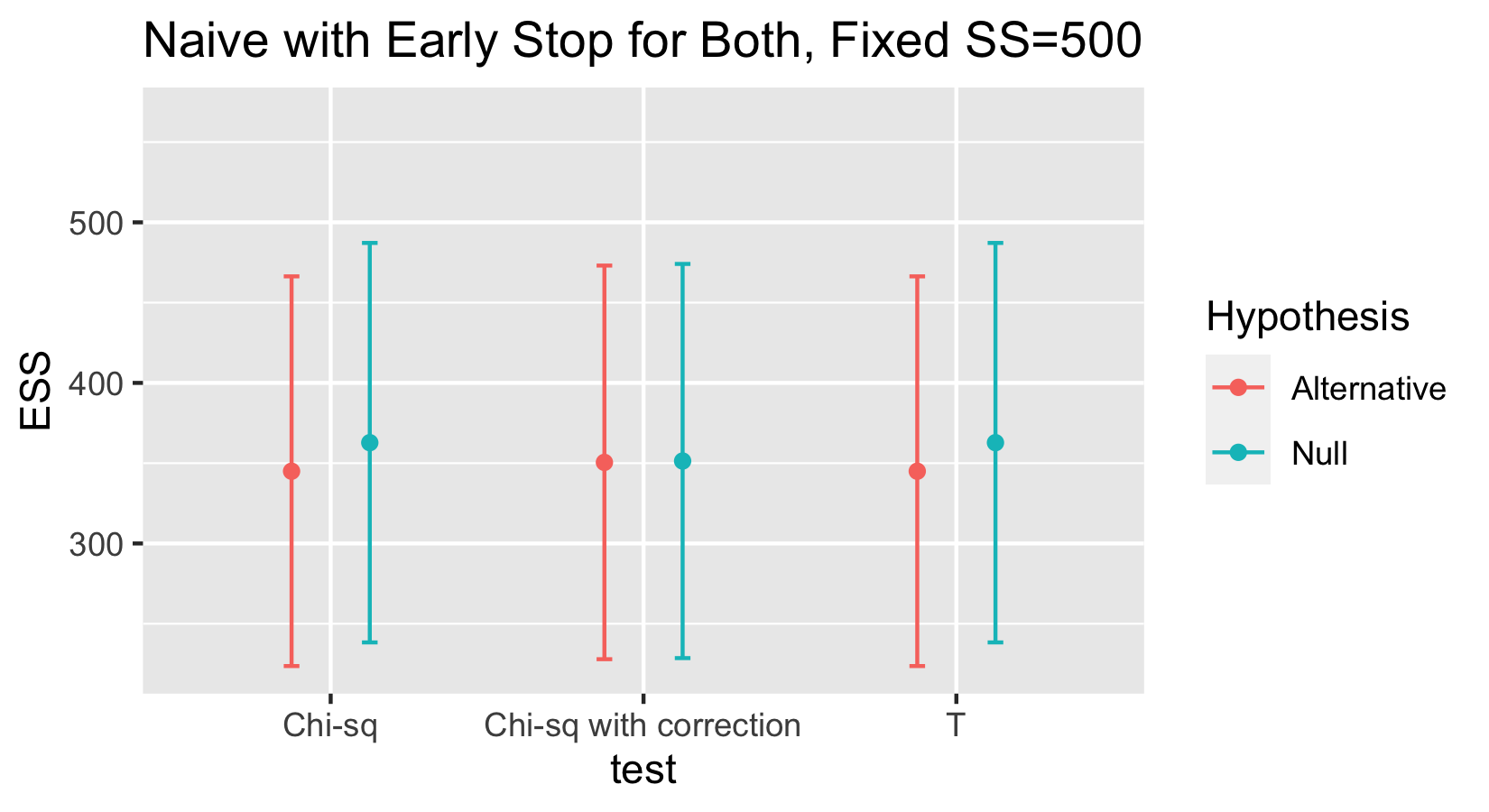


##
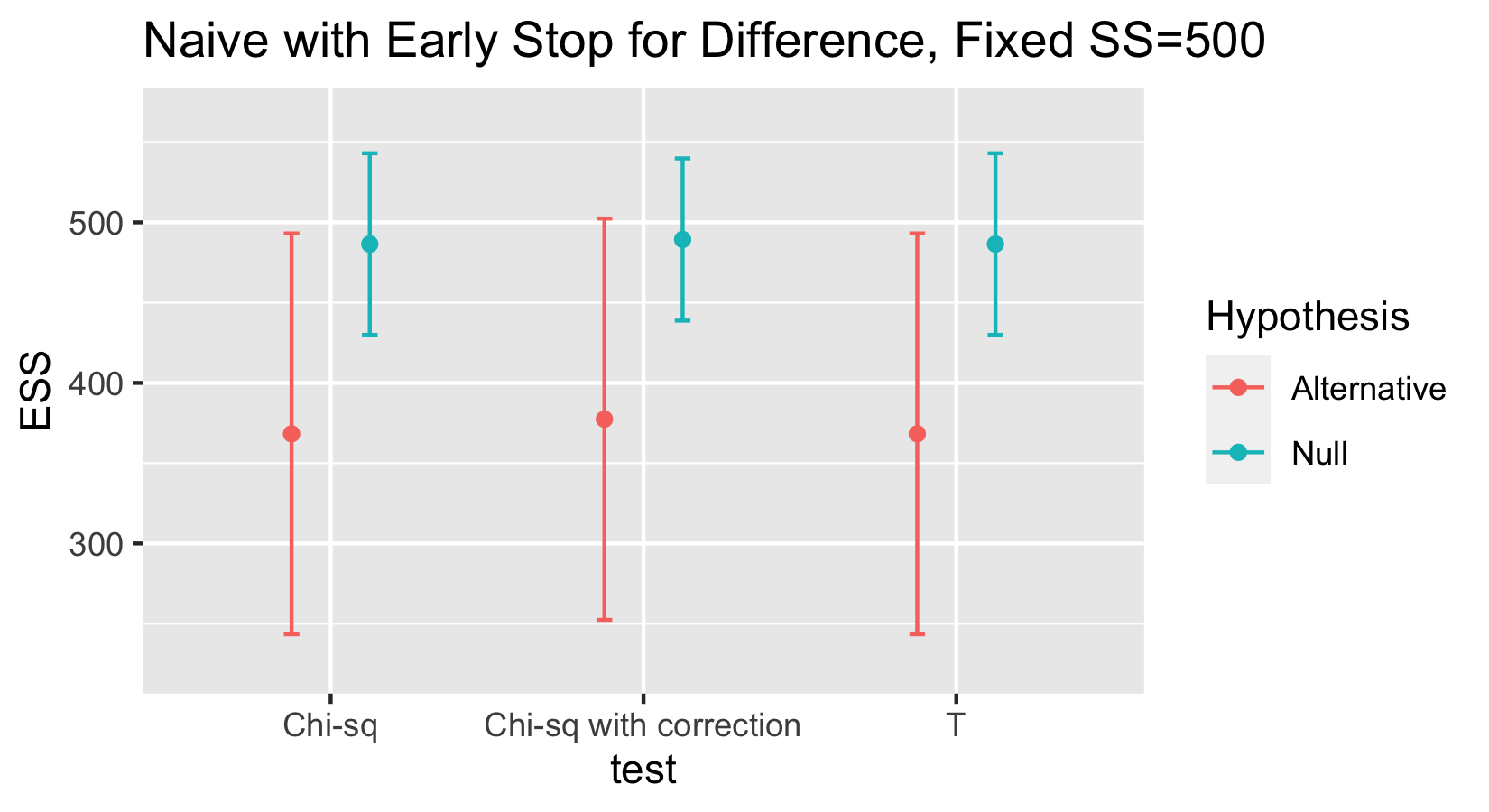

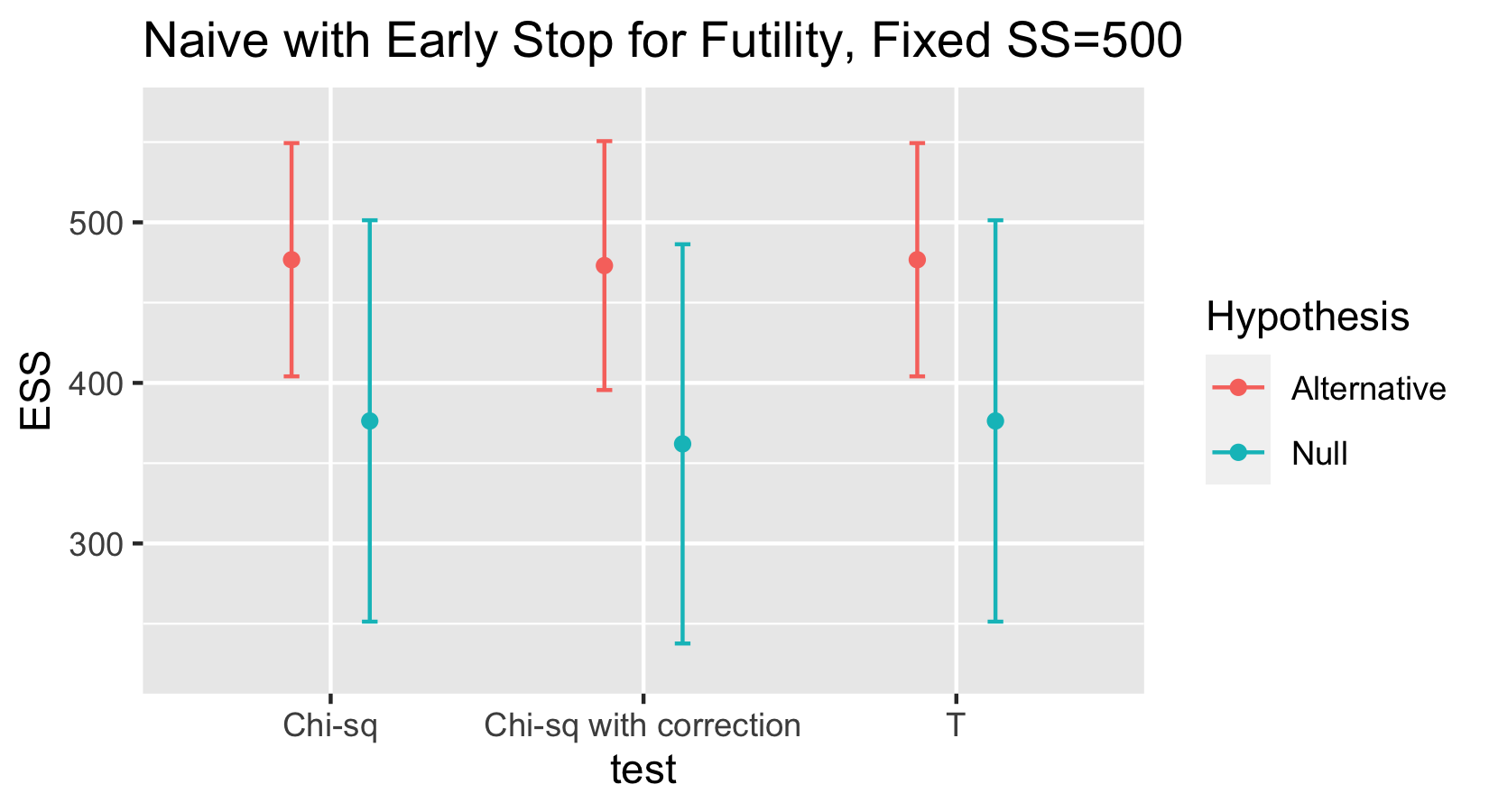

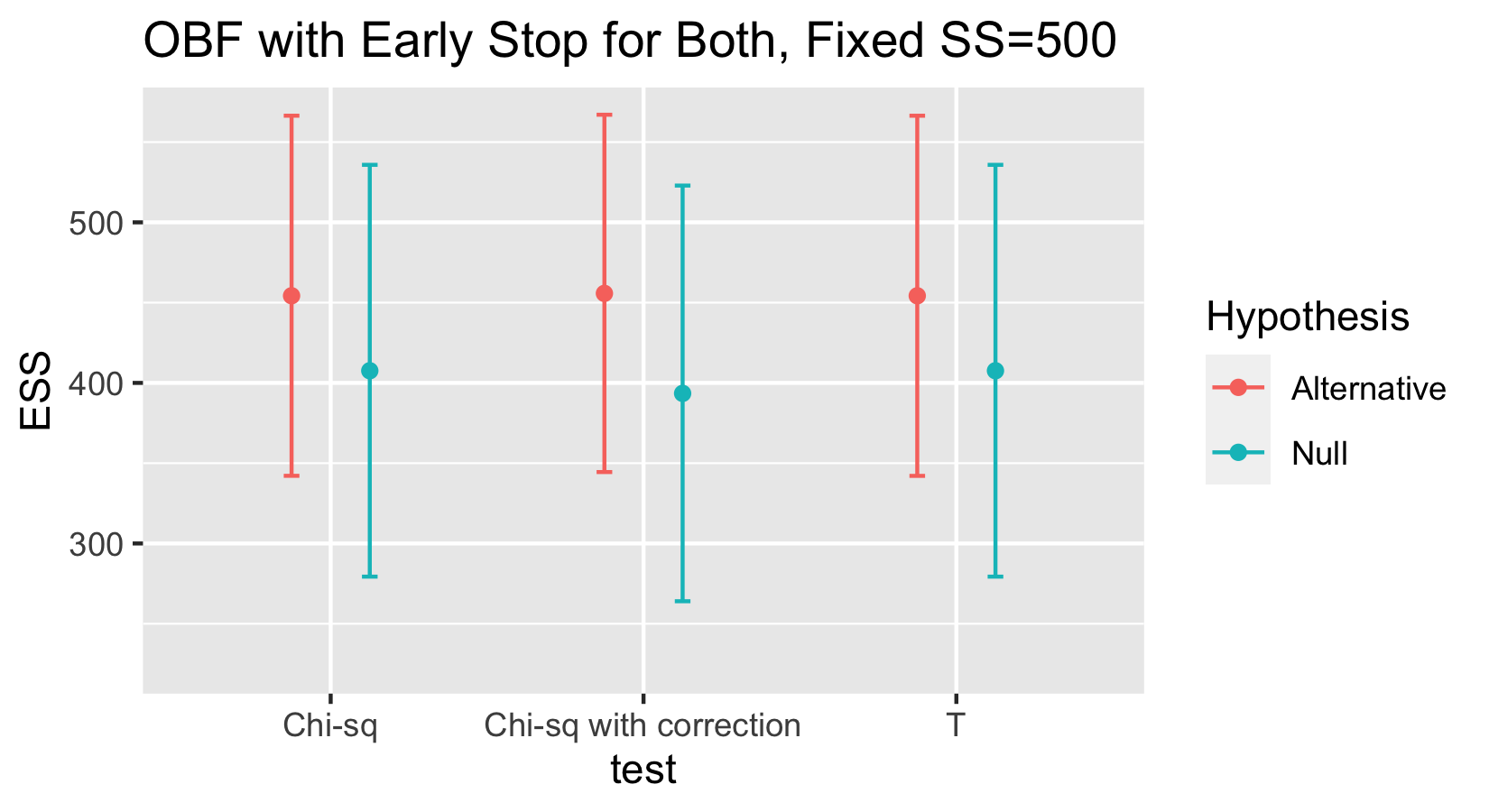


##
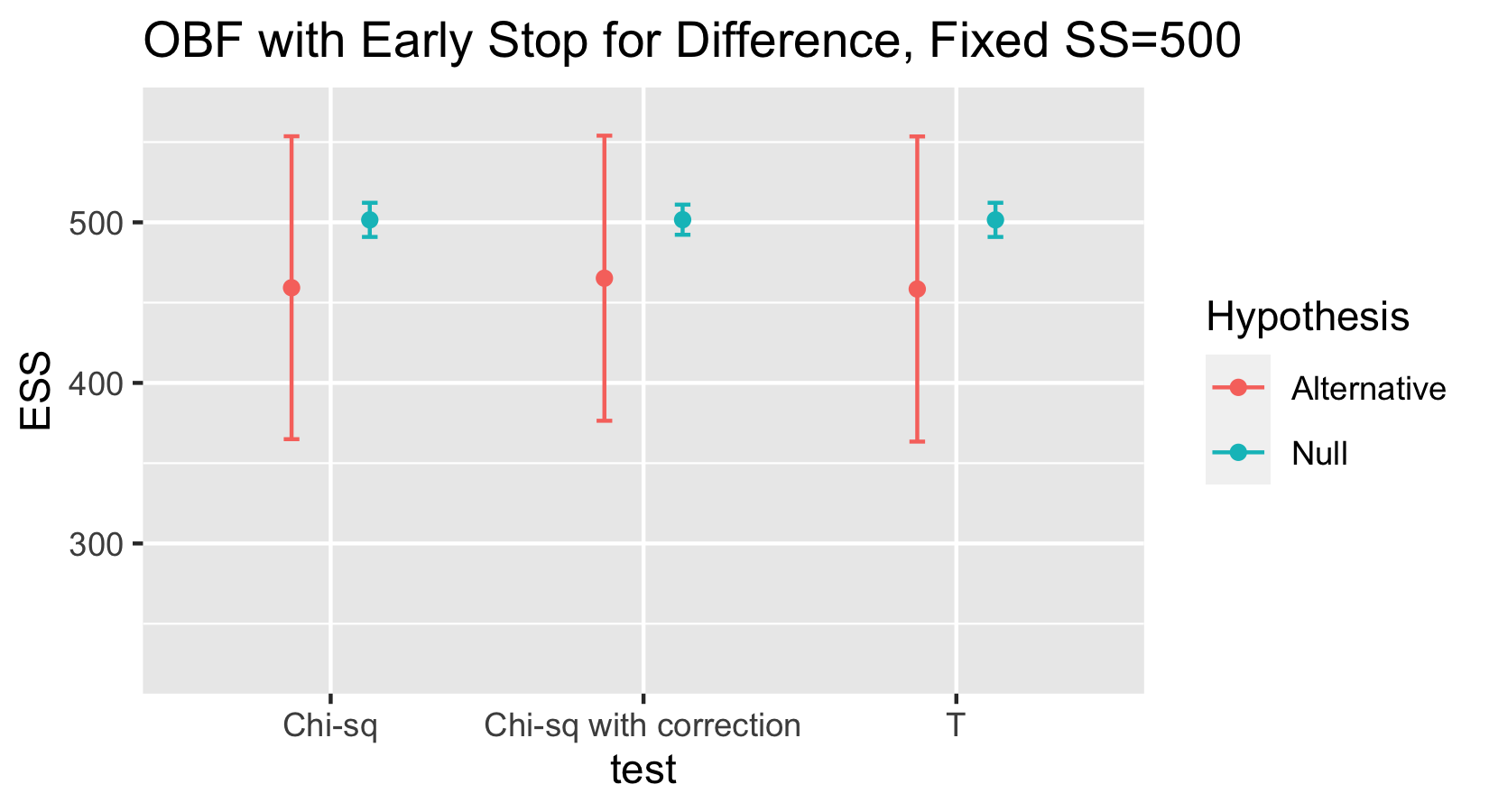

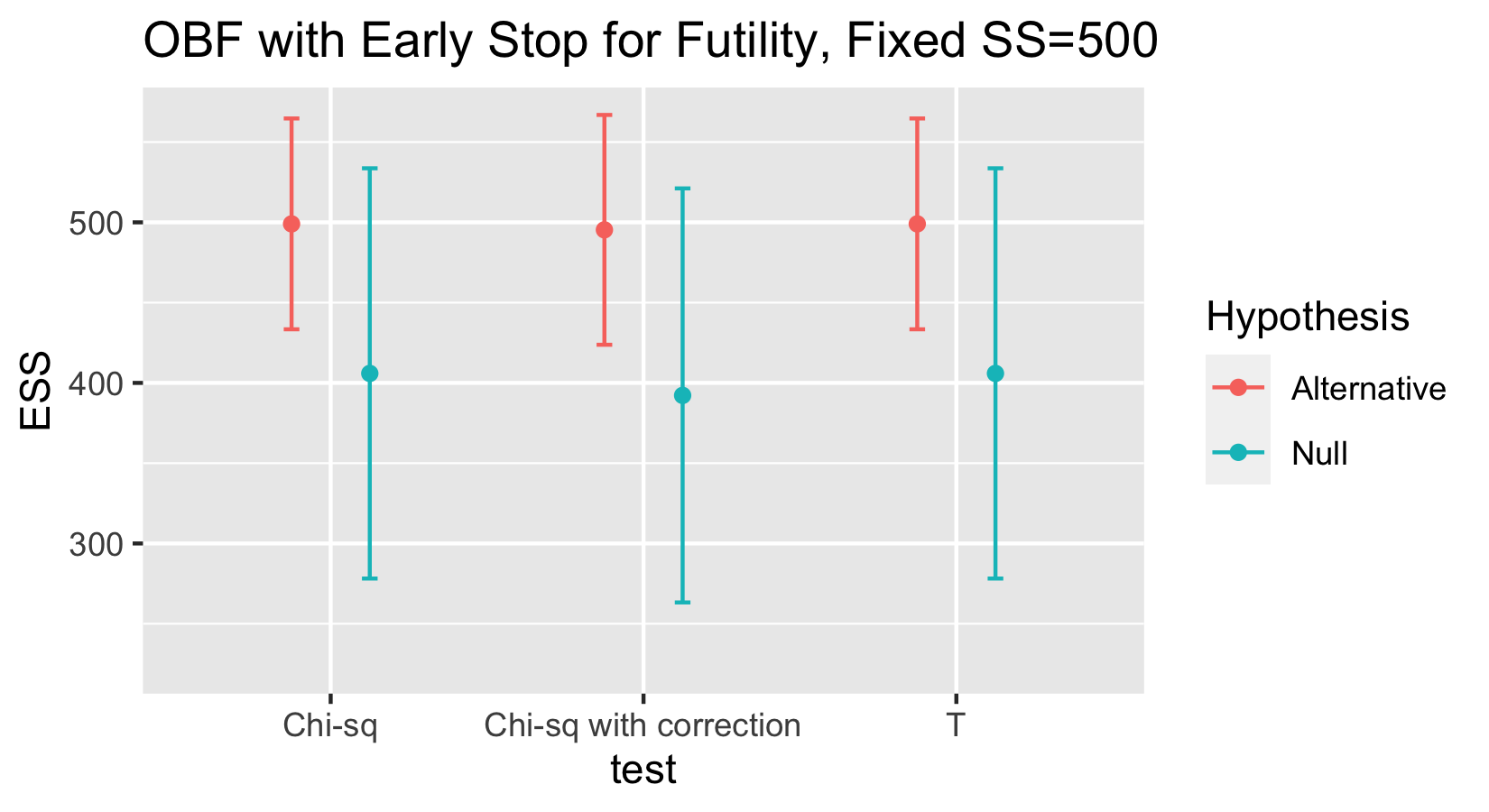

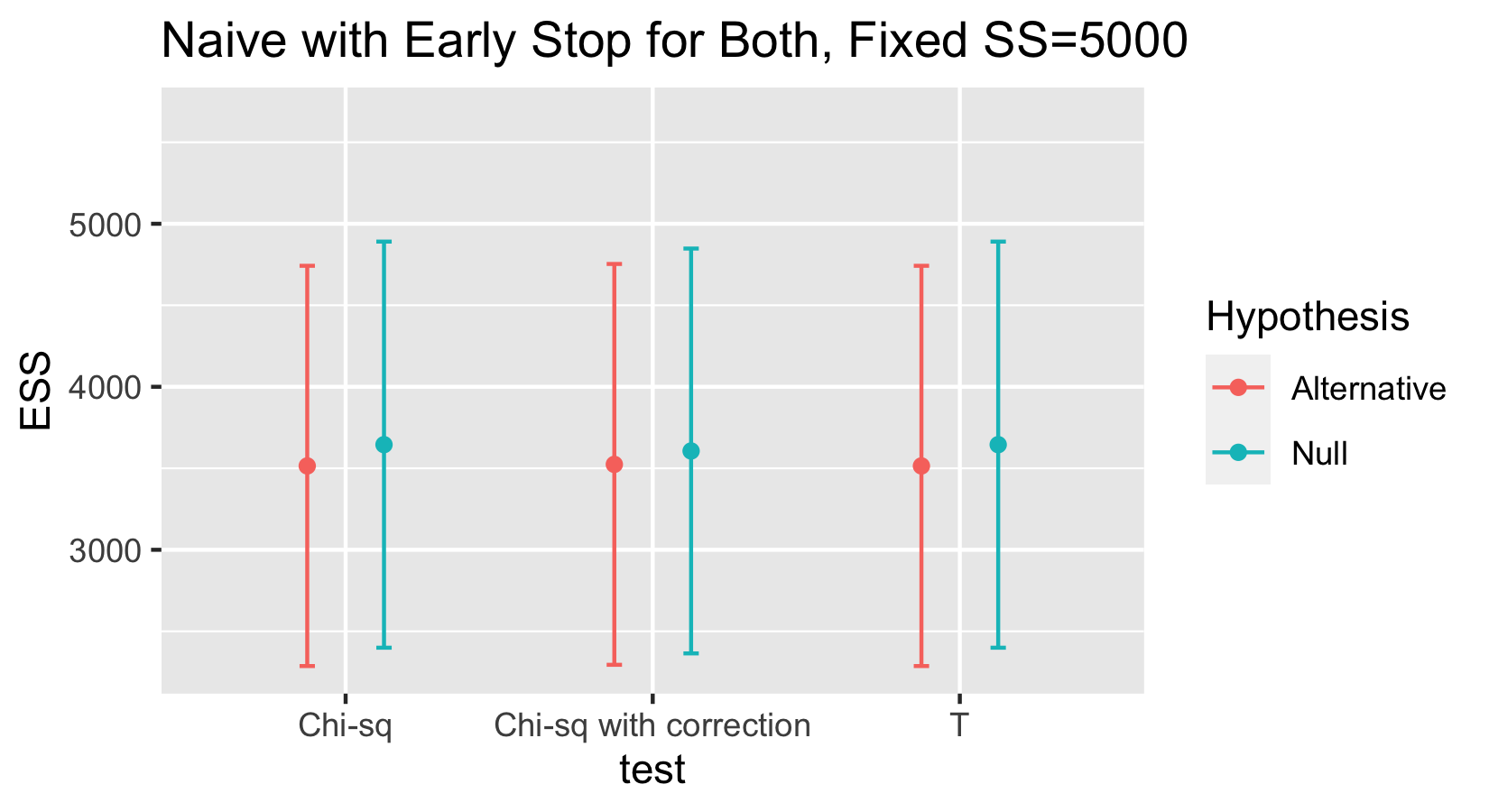


##
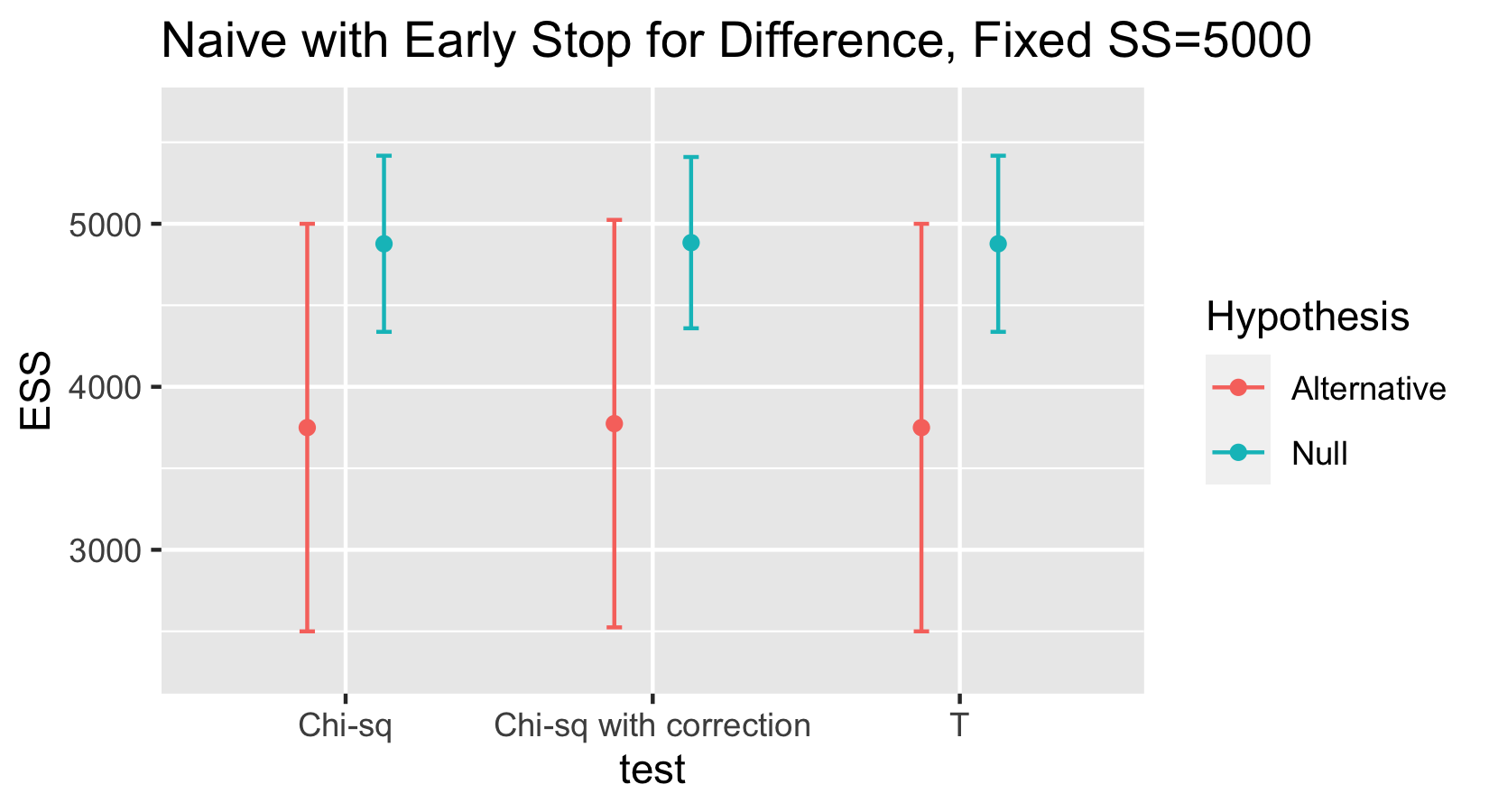


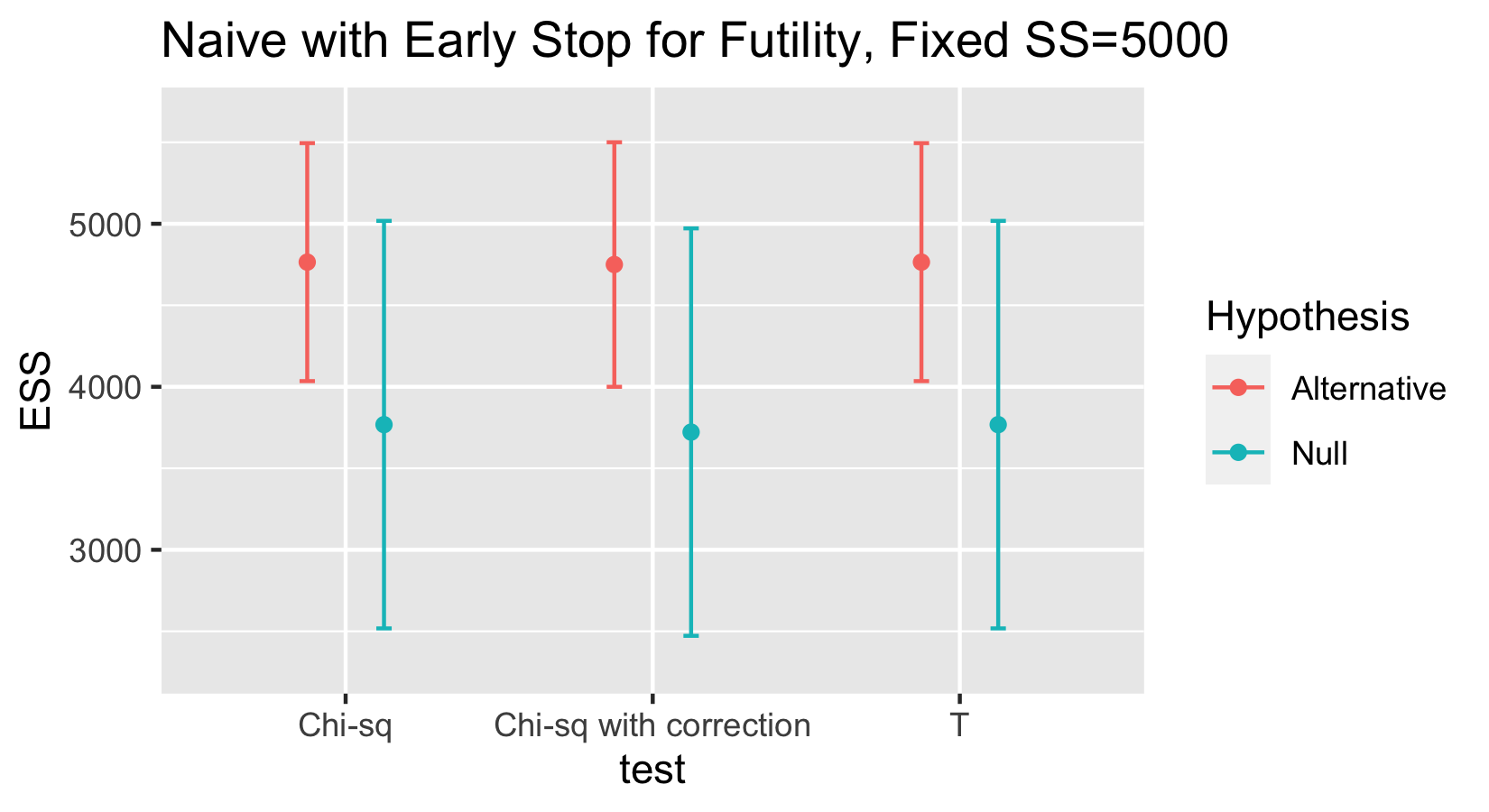

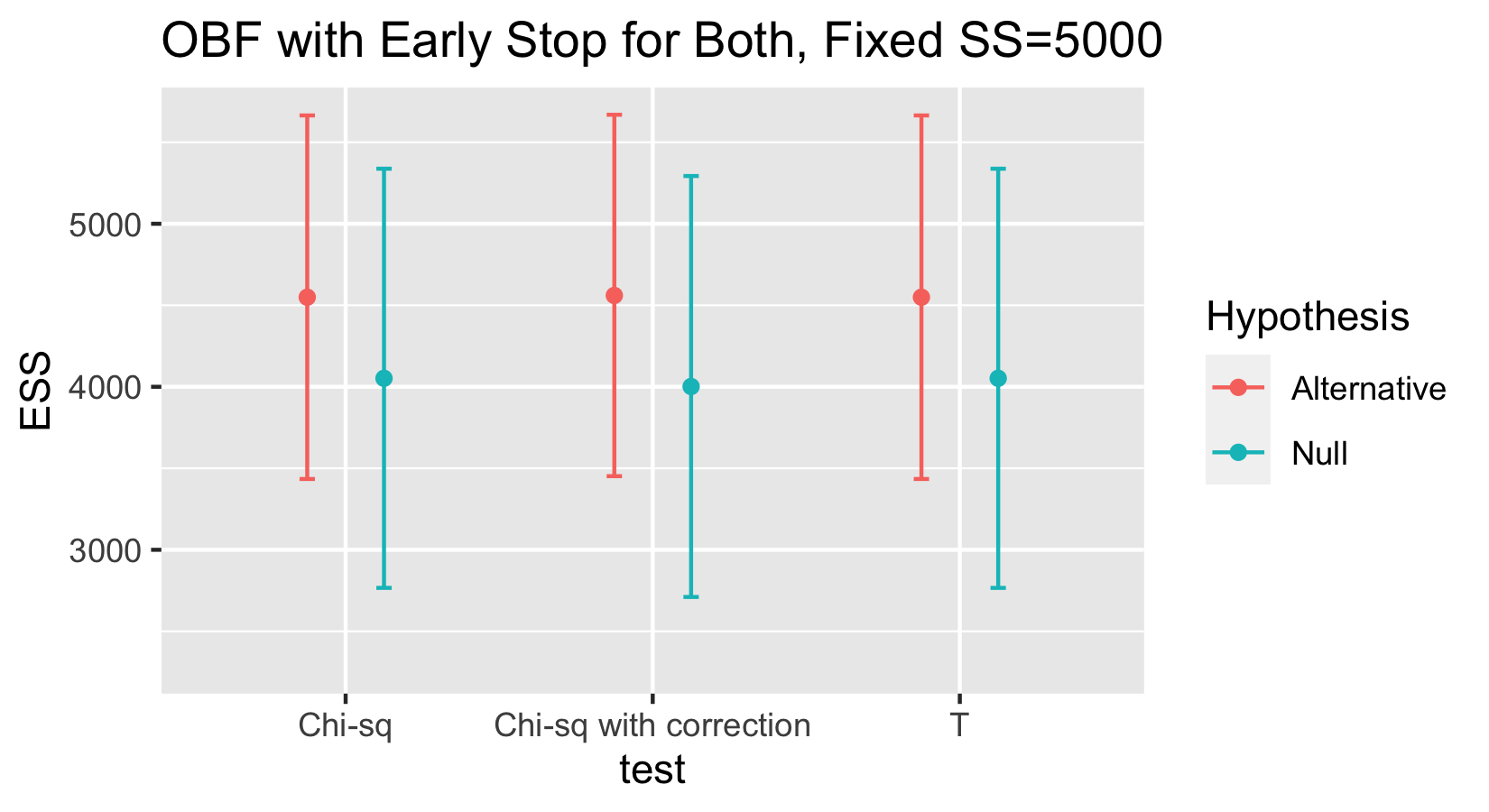


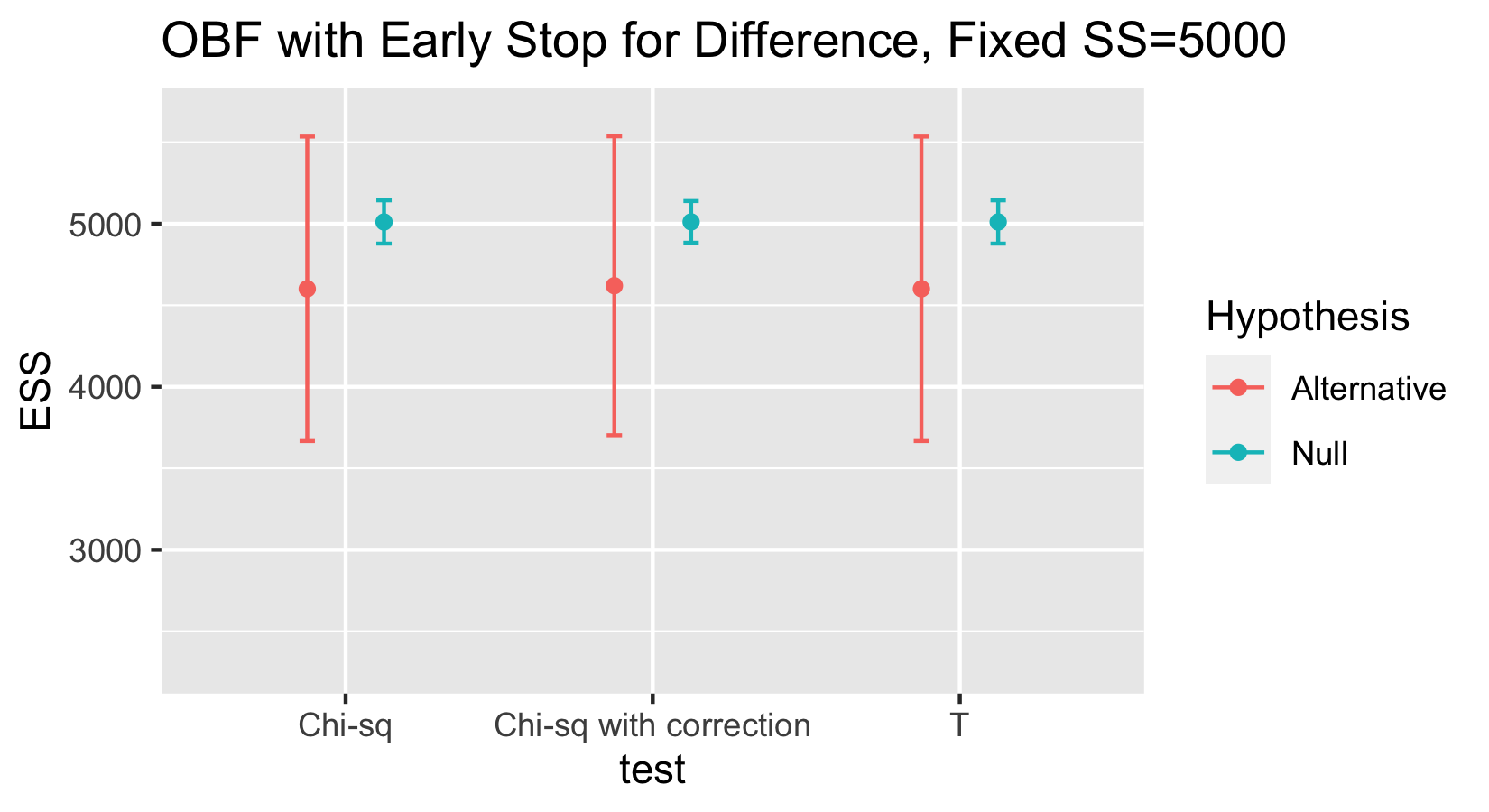

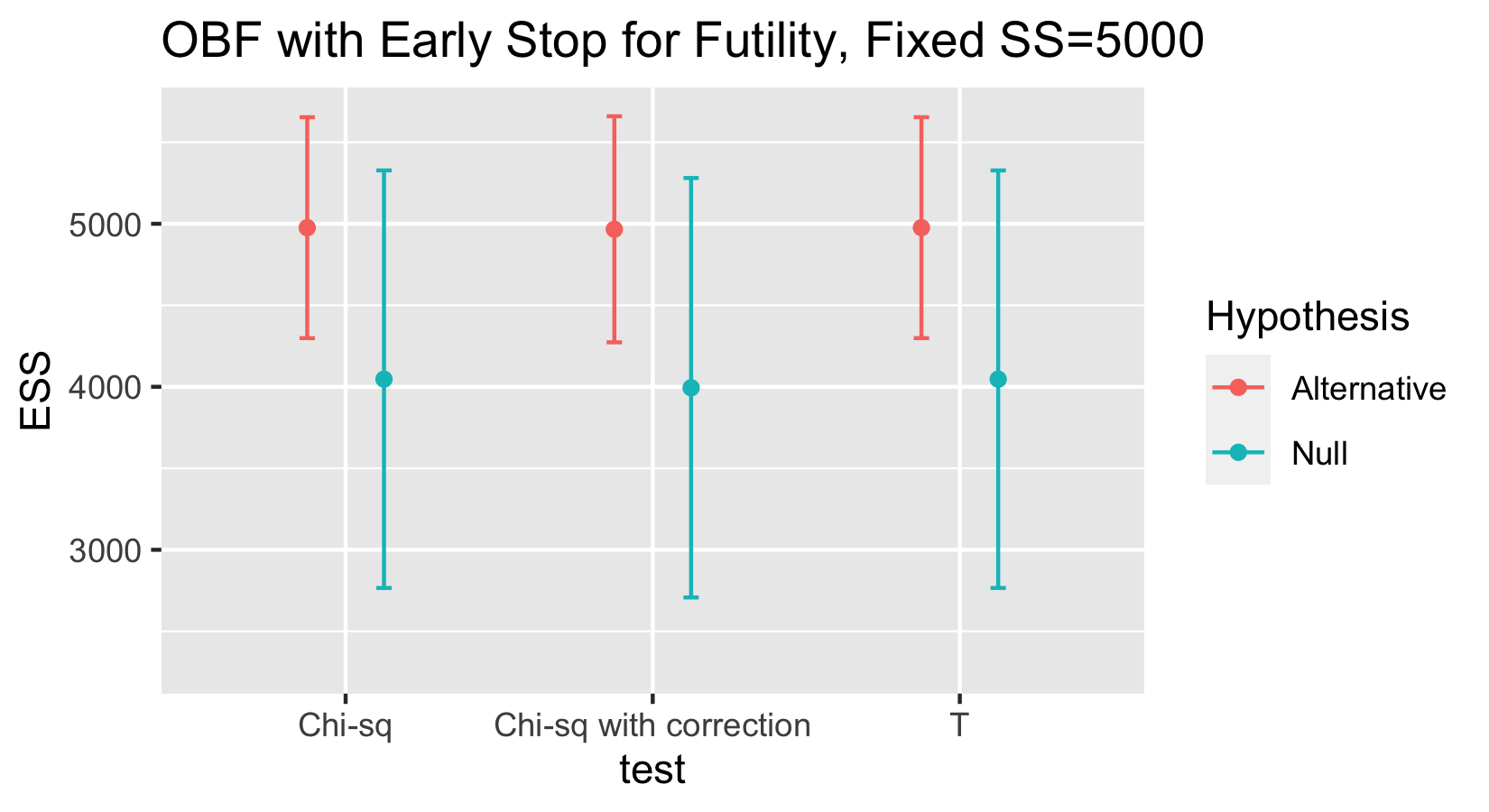


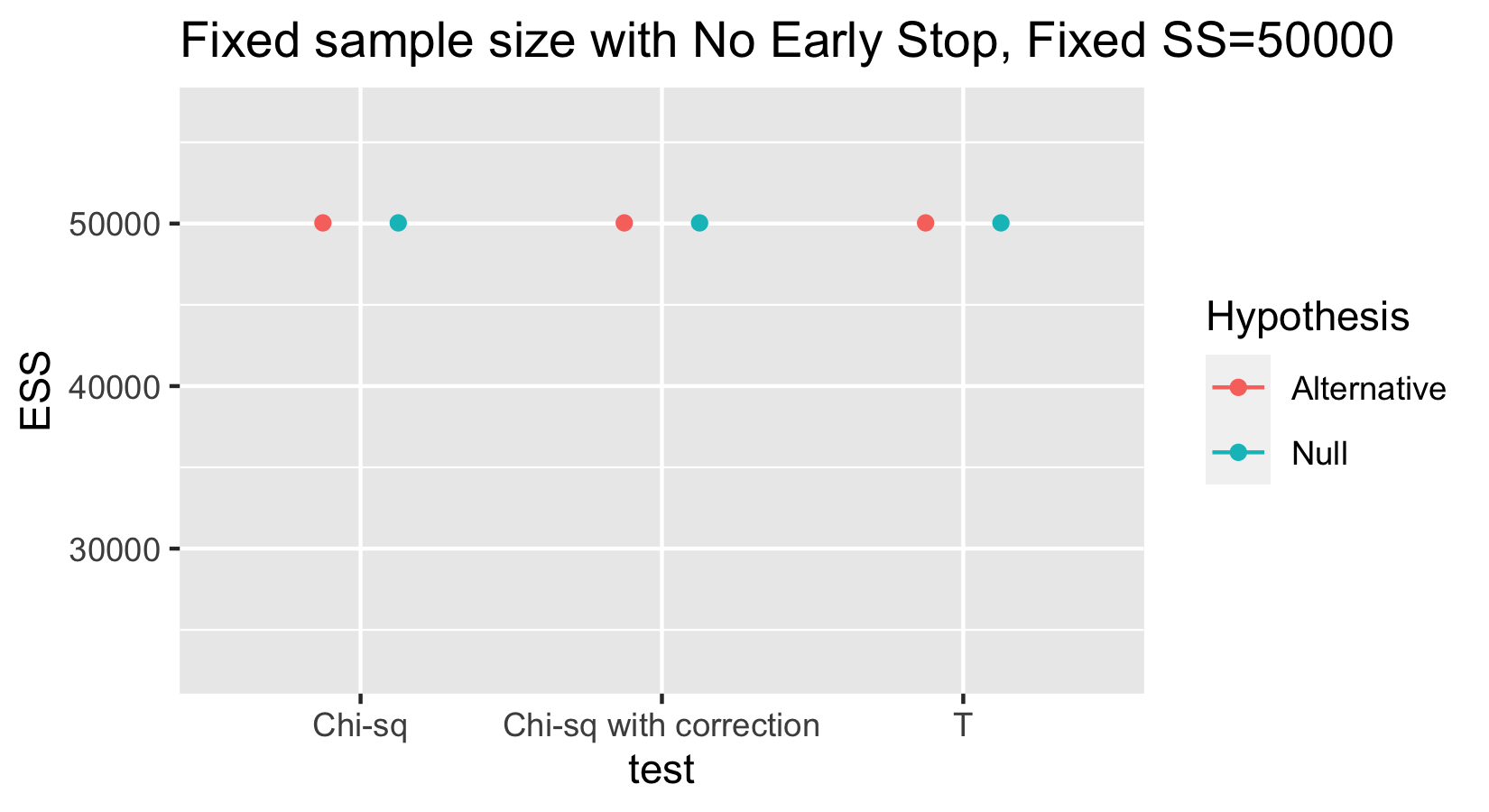


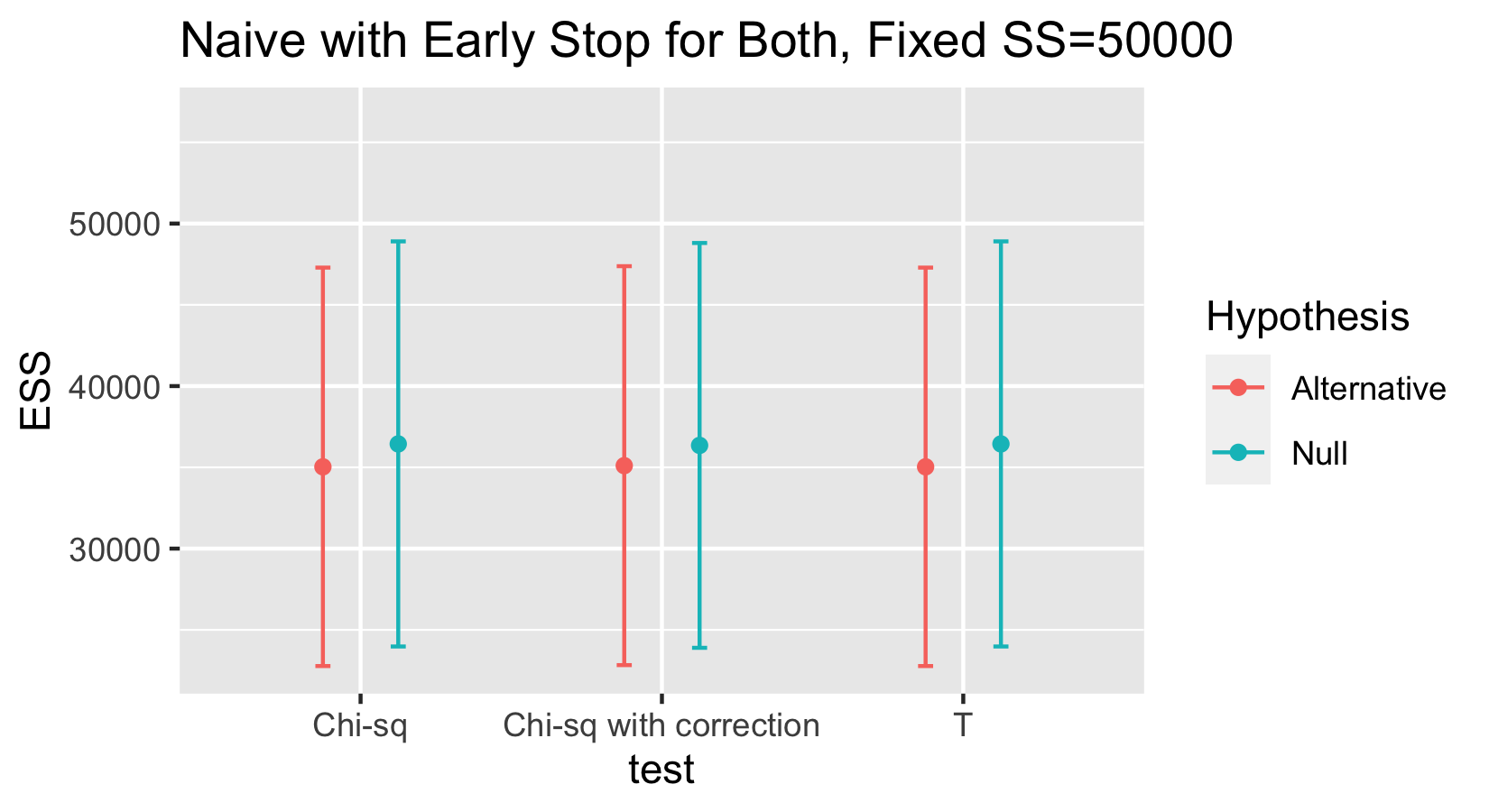

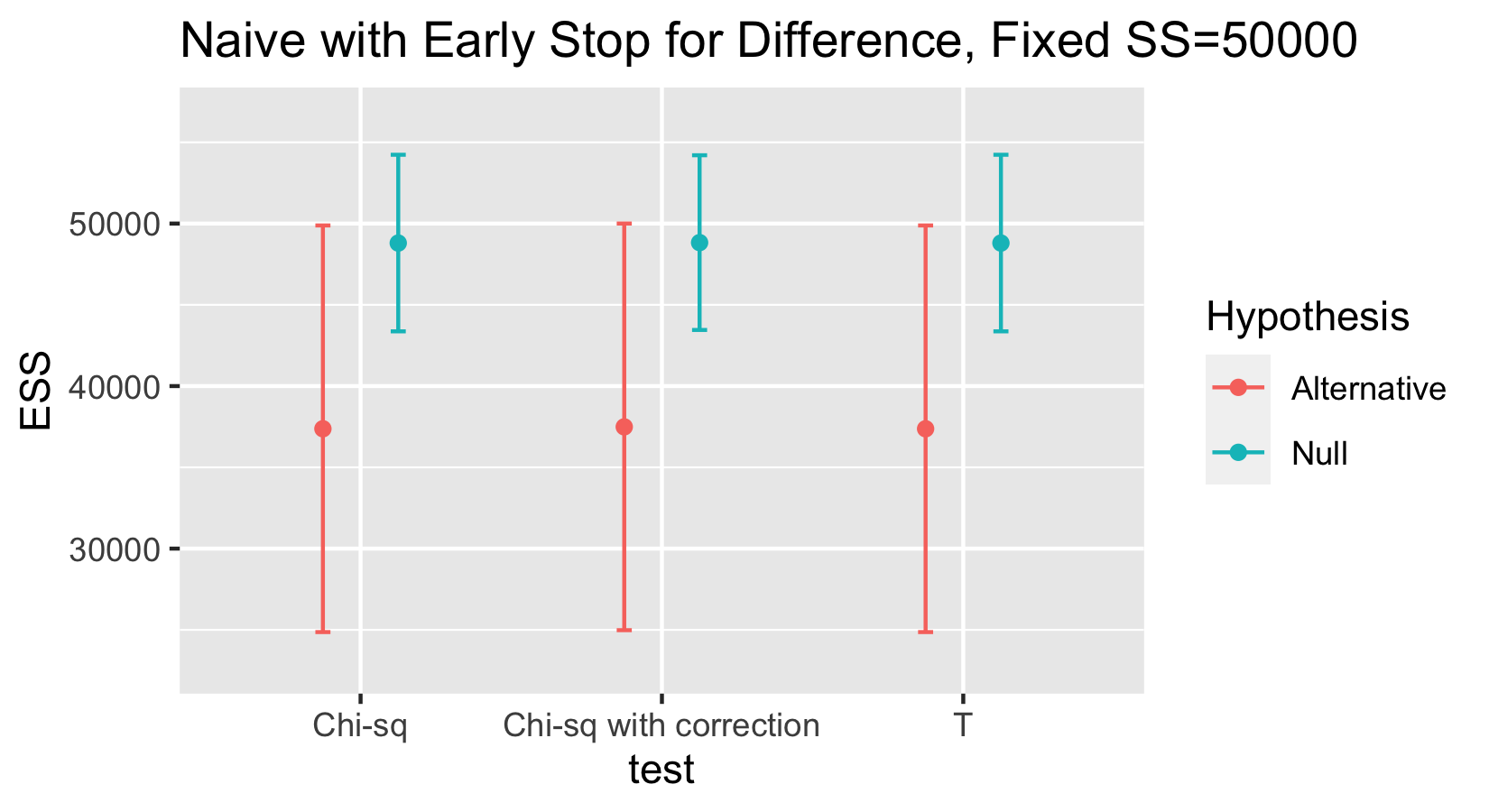

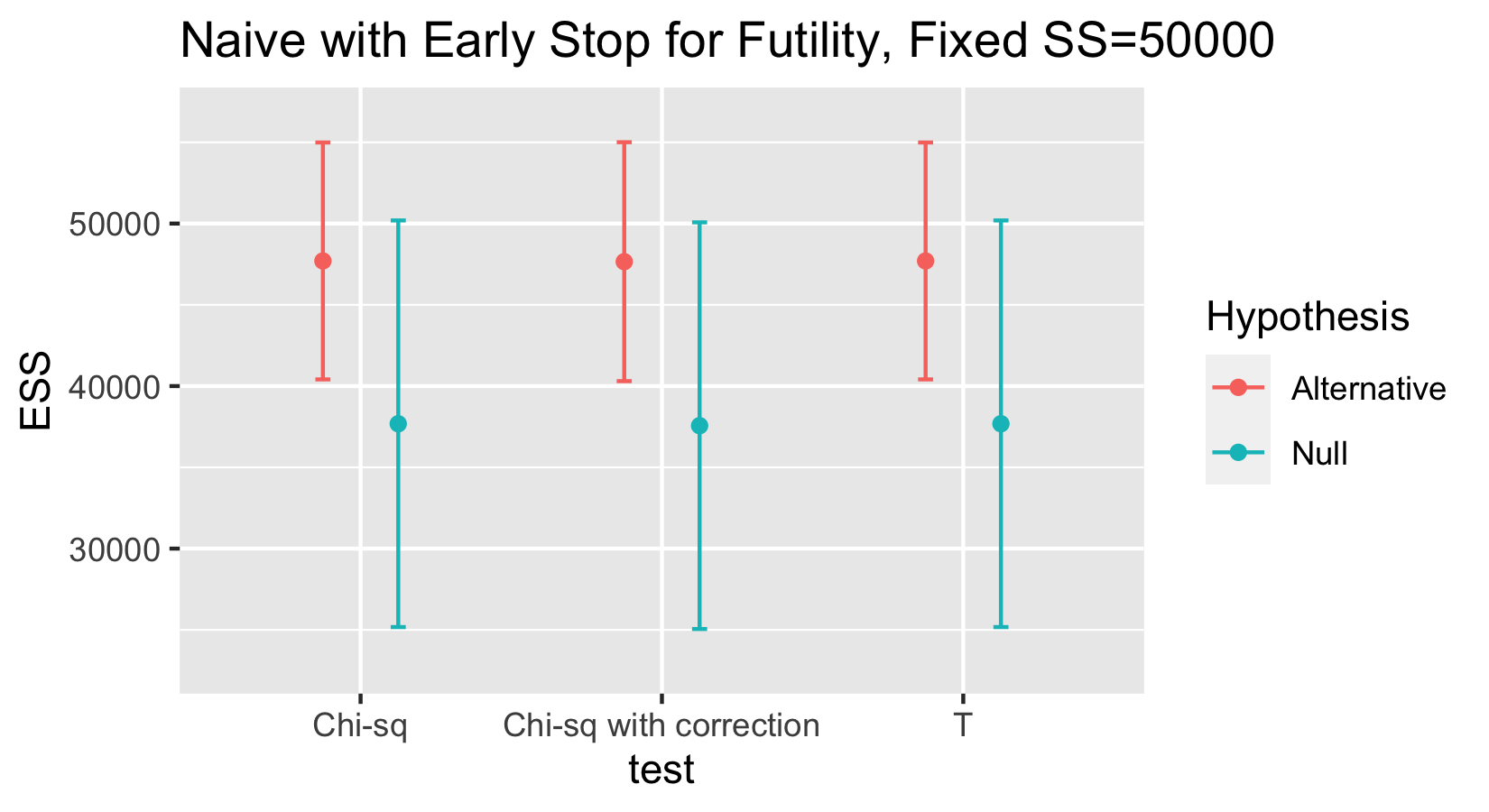


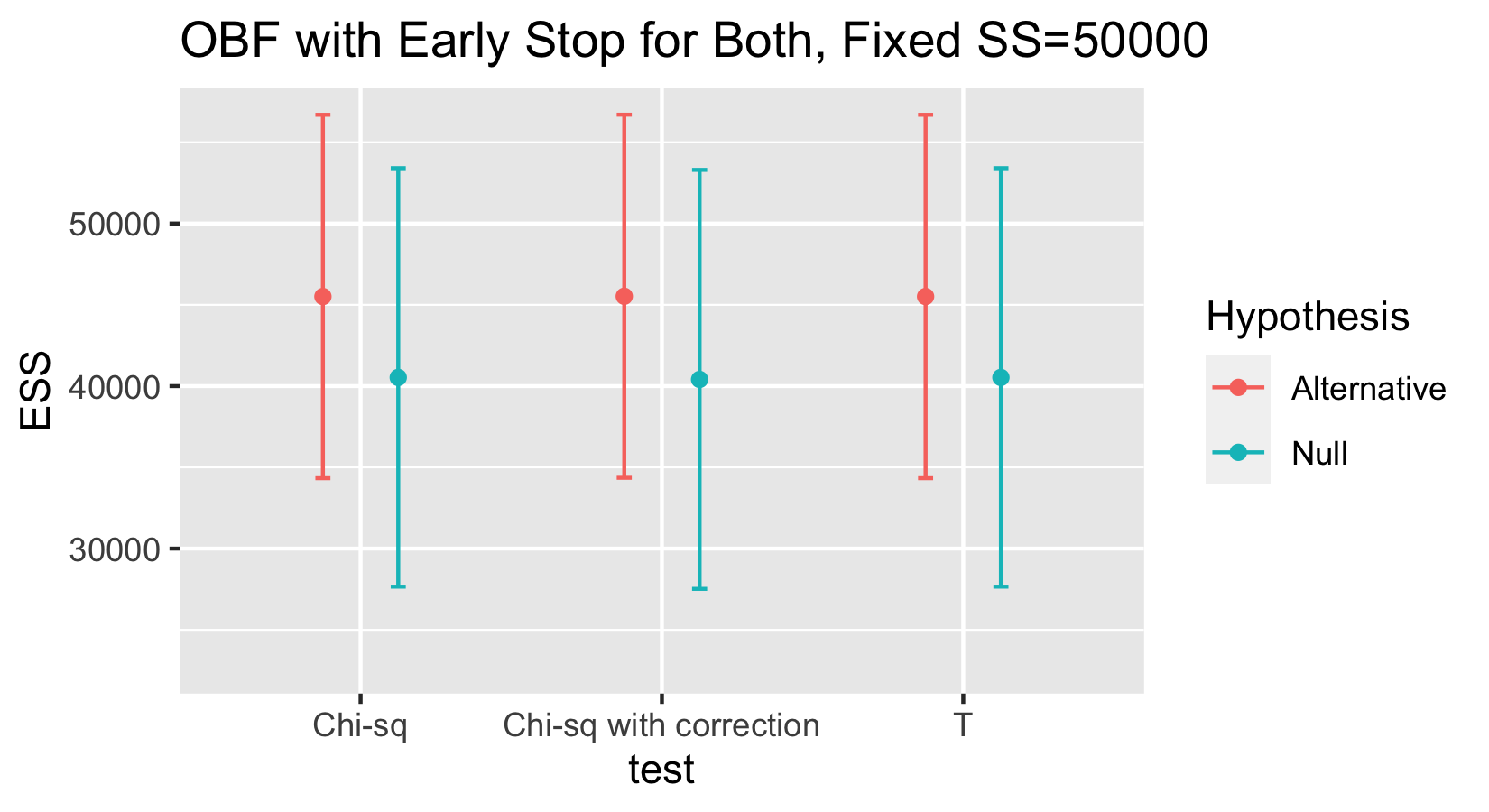

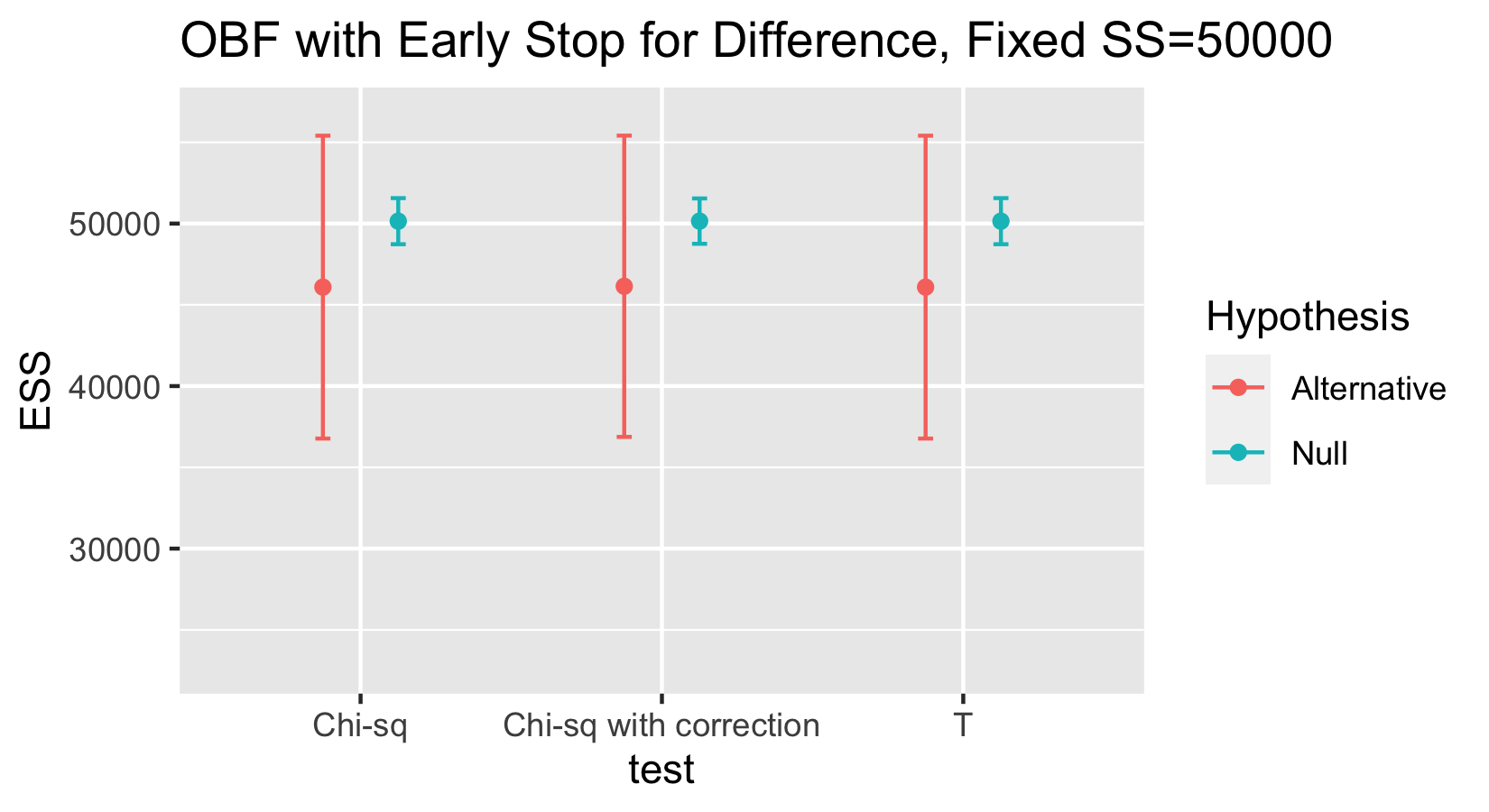

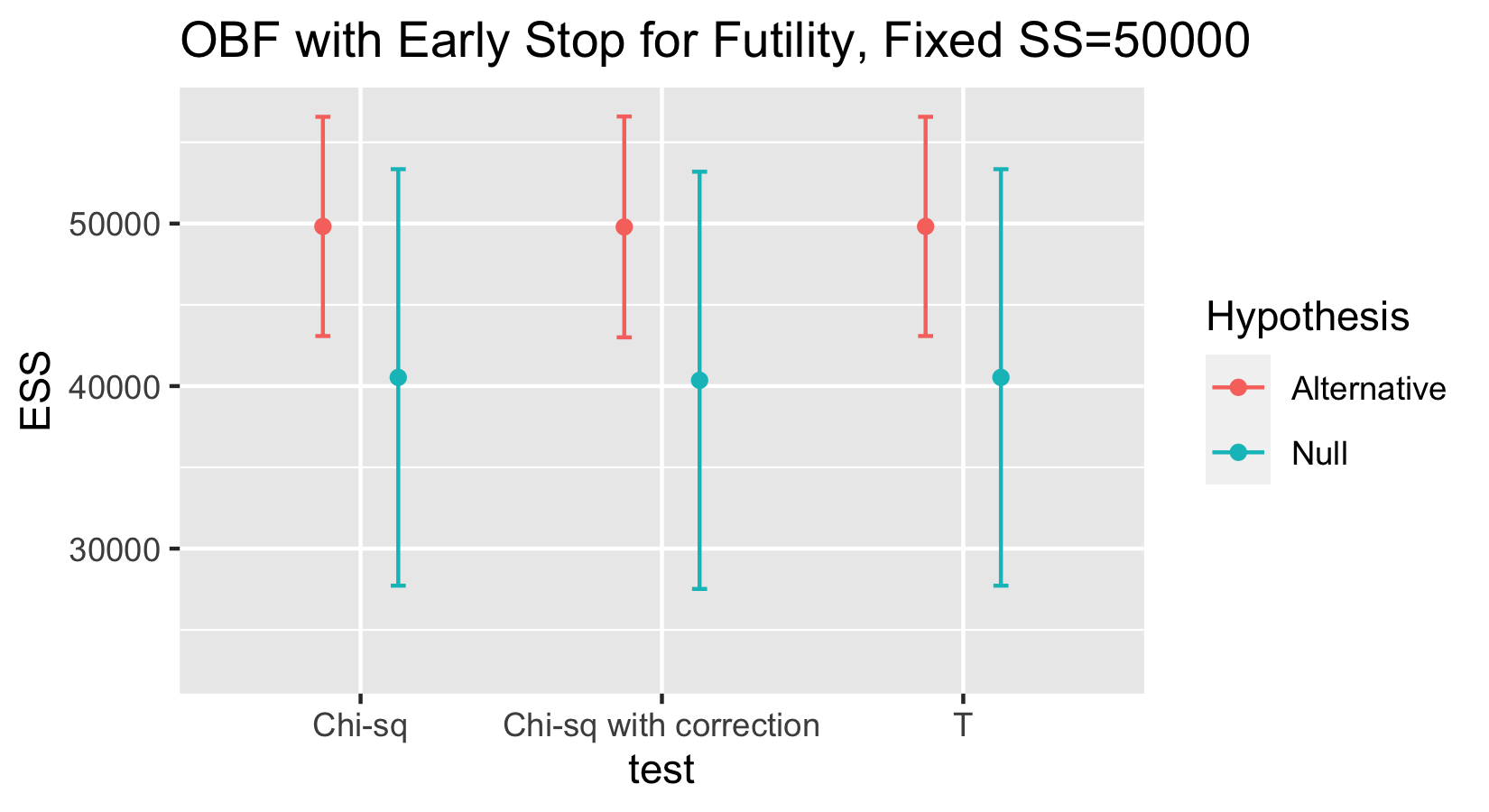


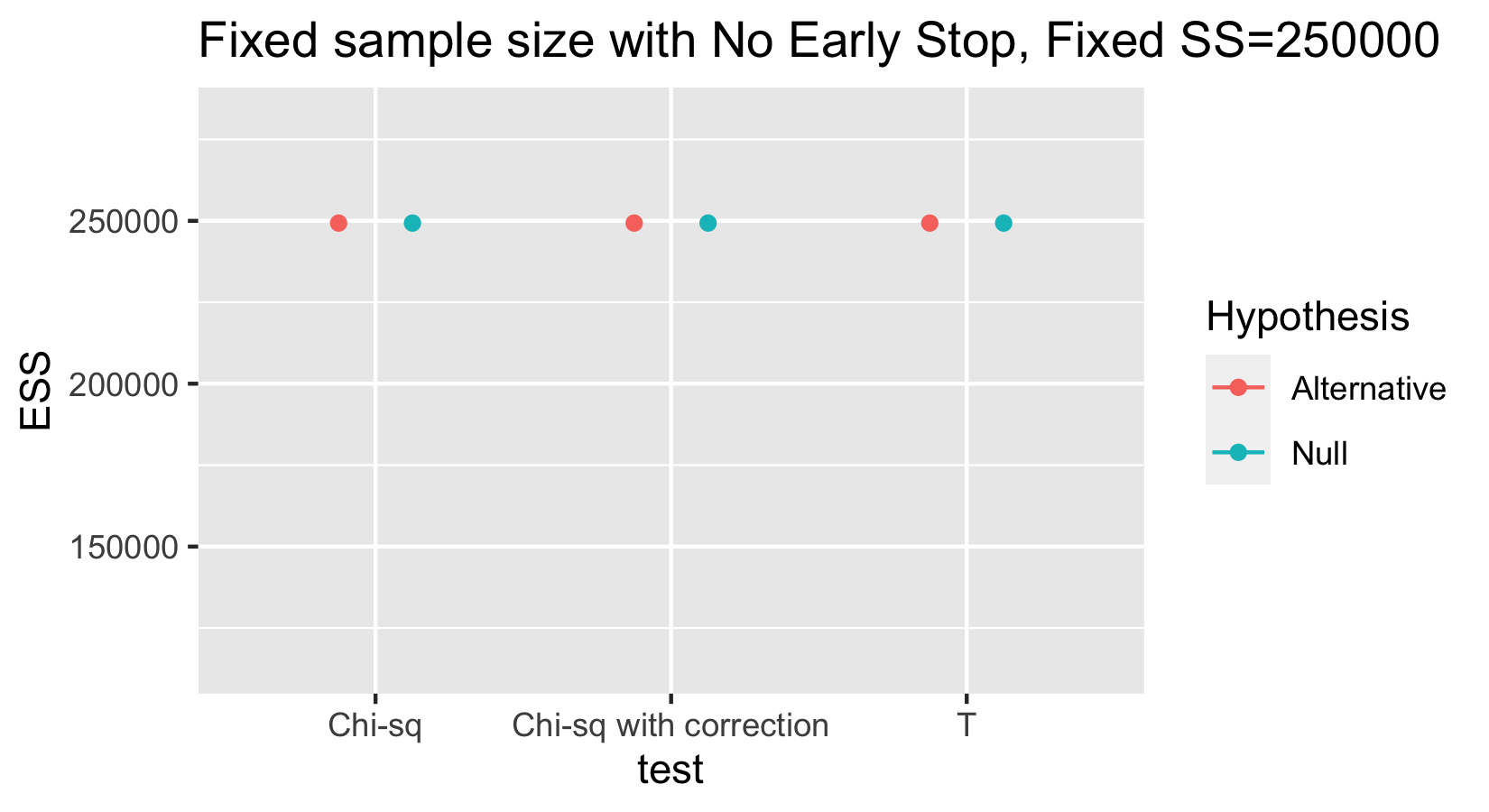

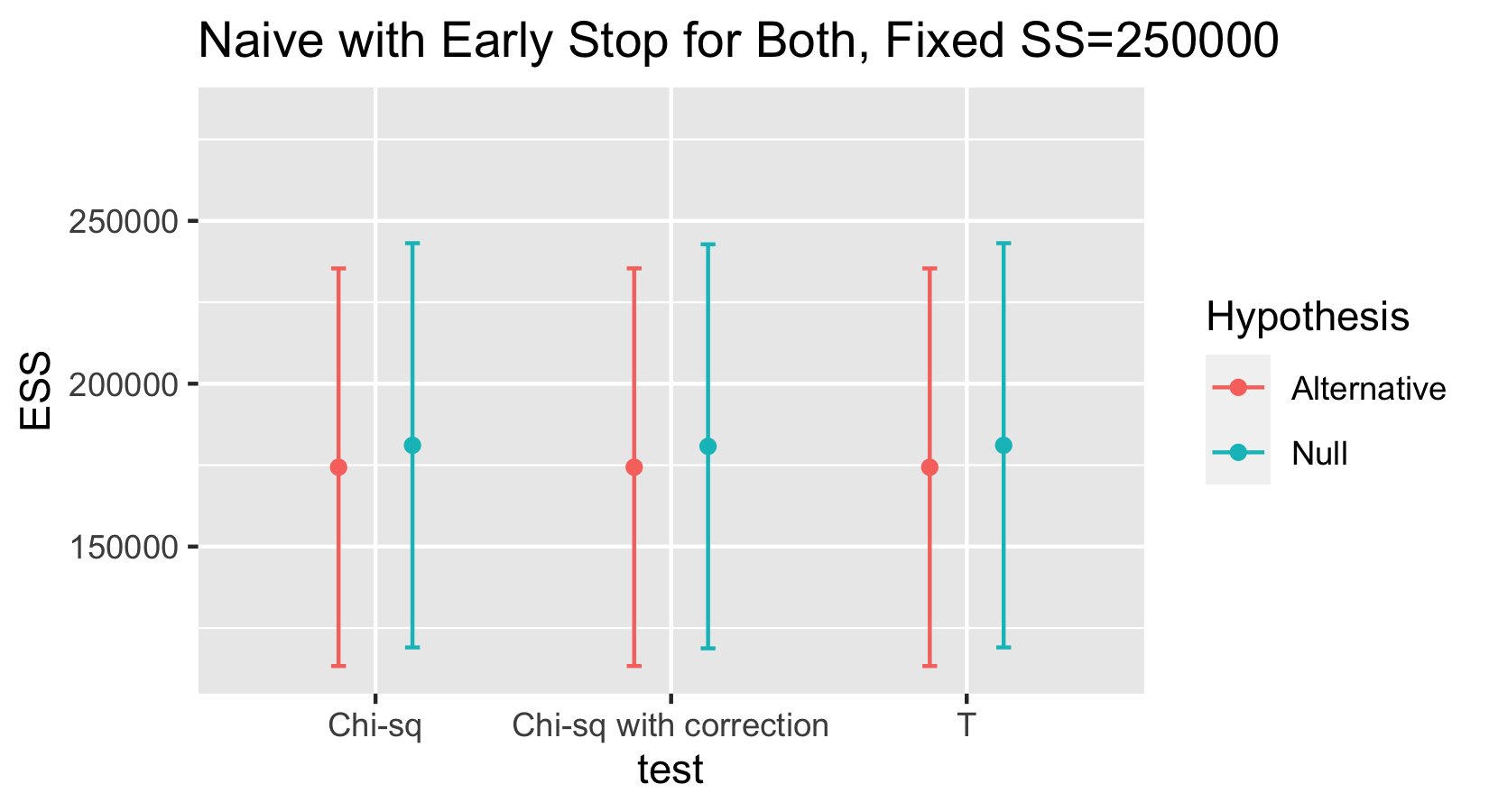

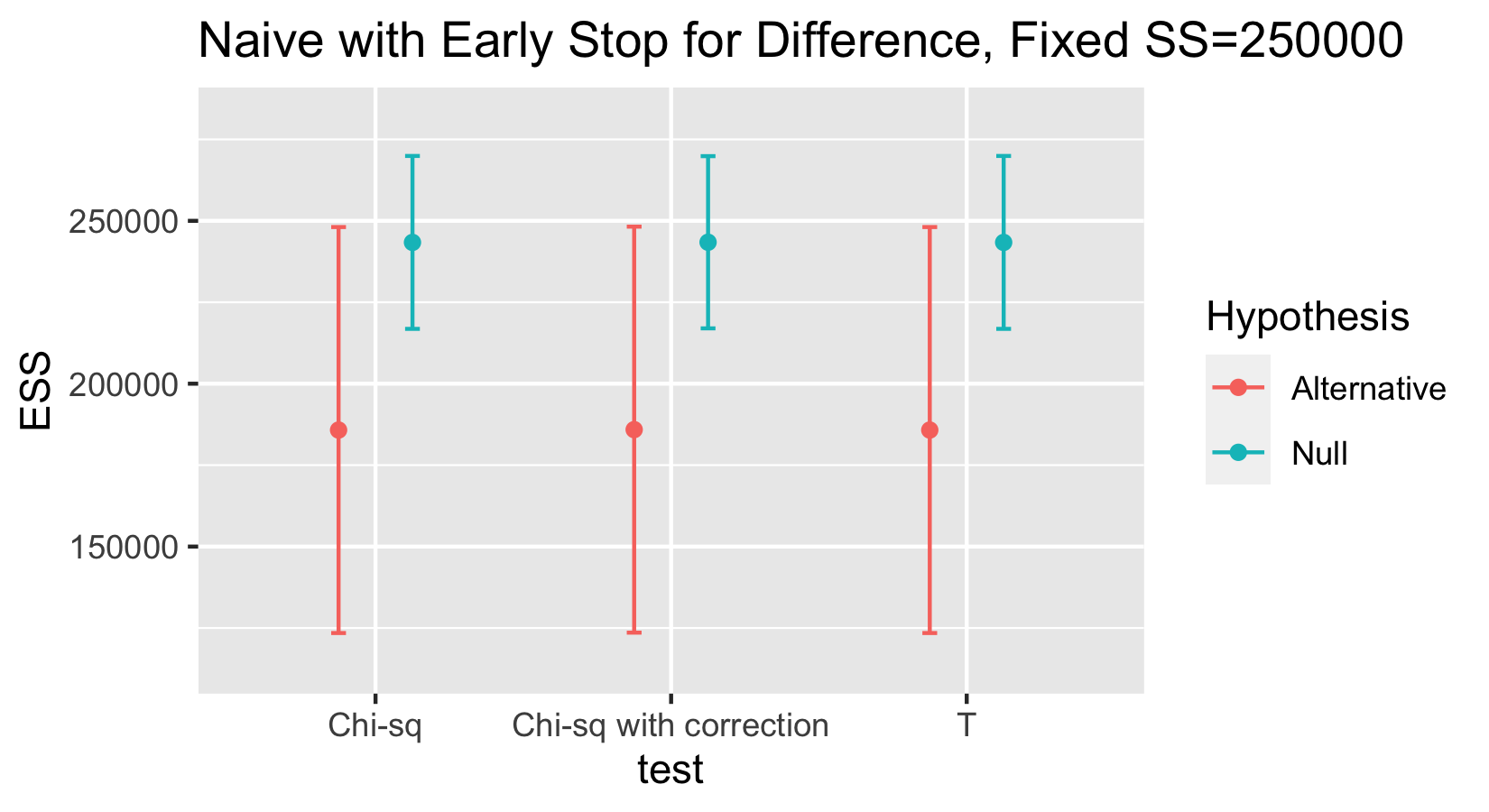


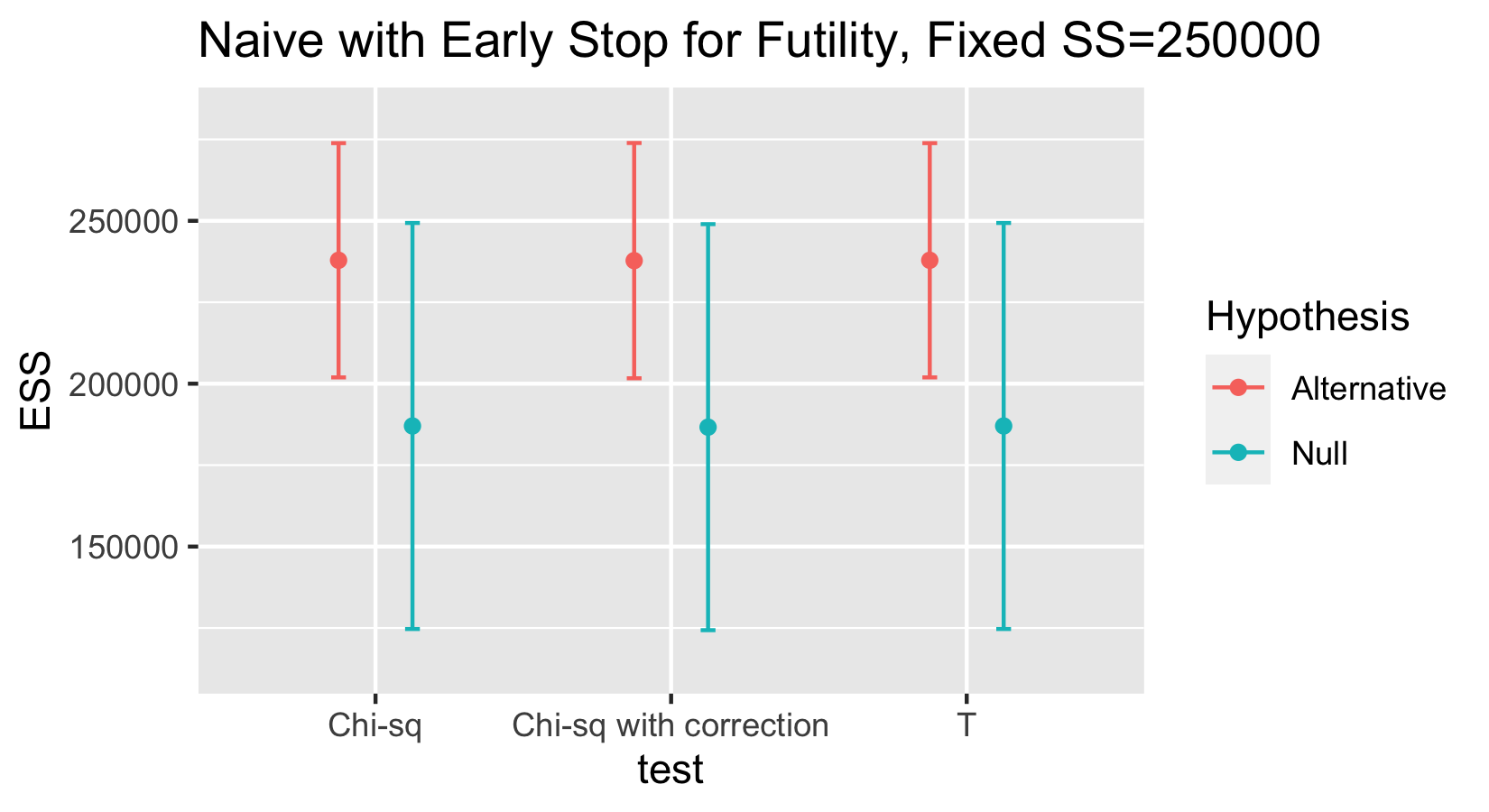

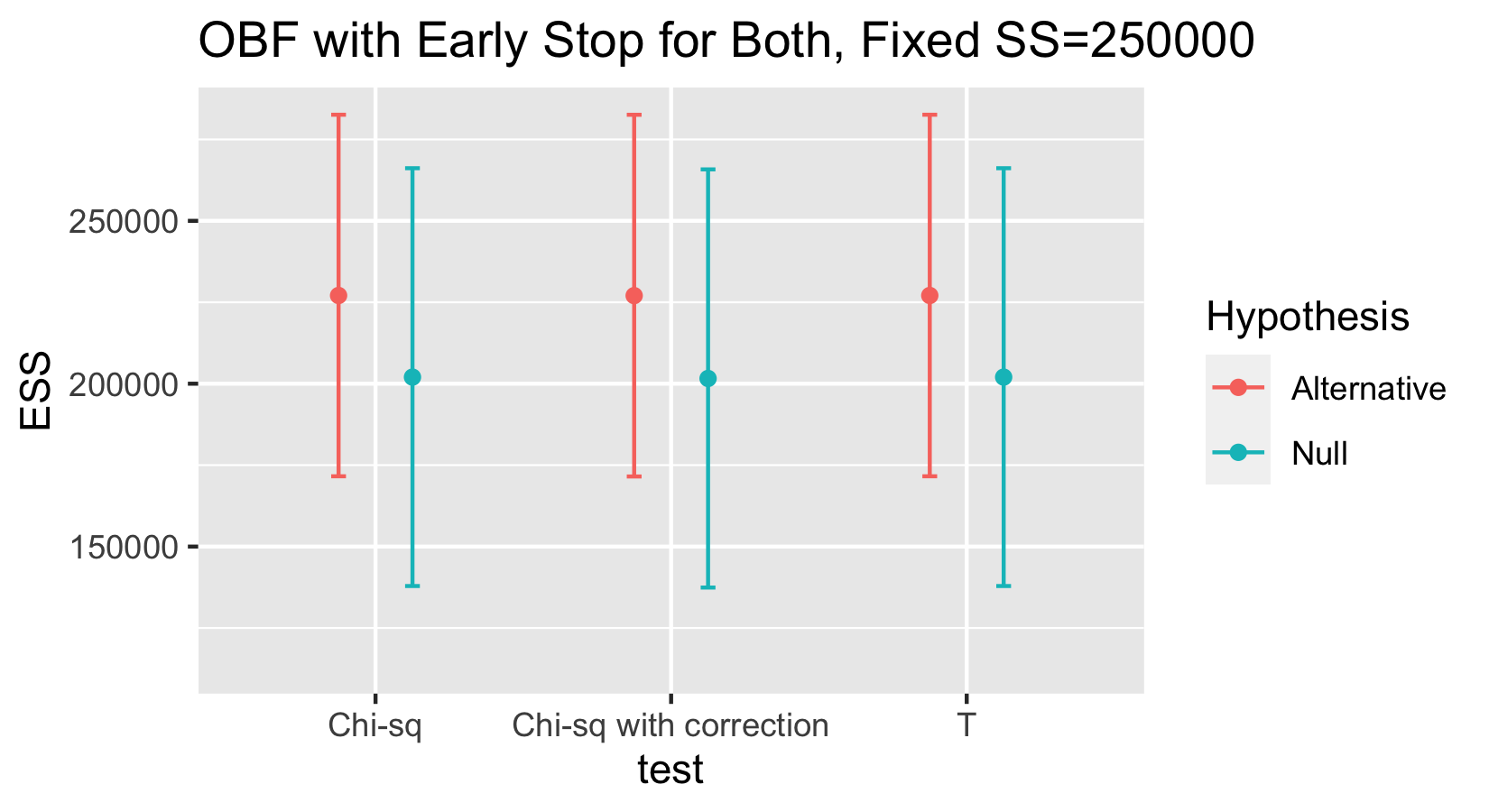

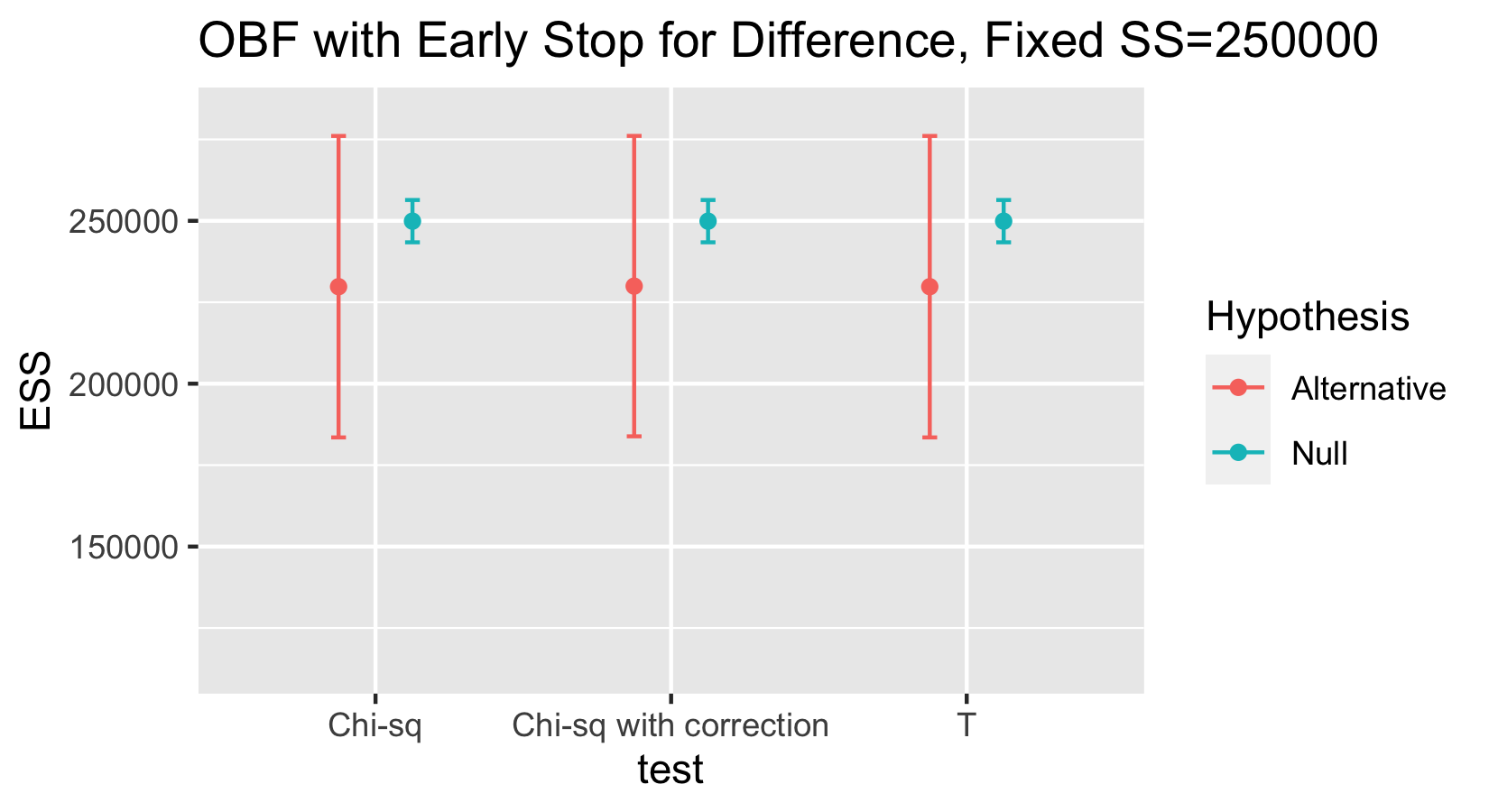


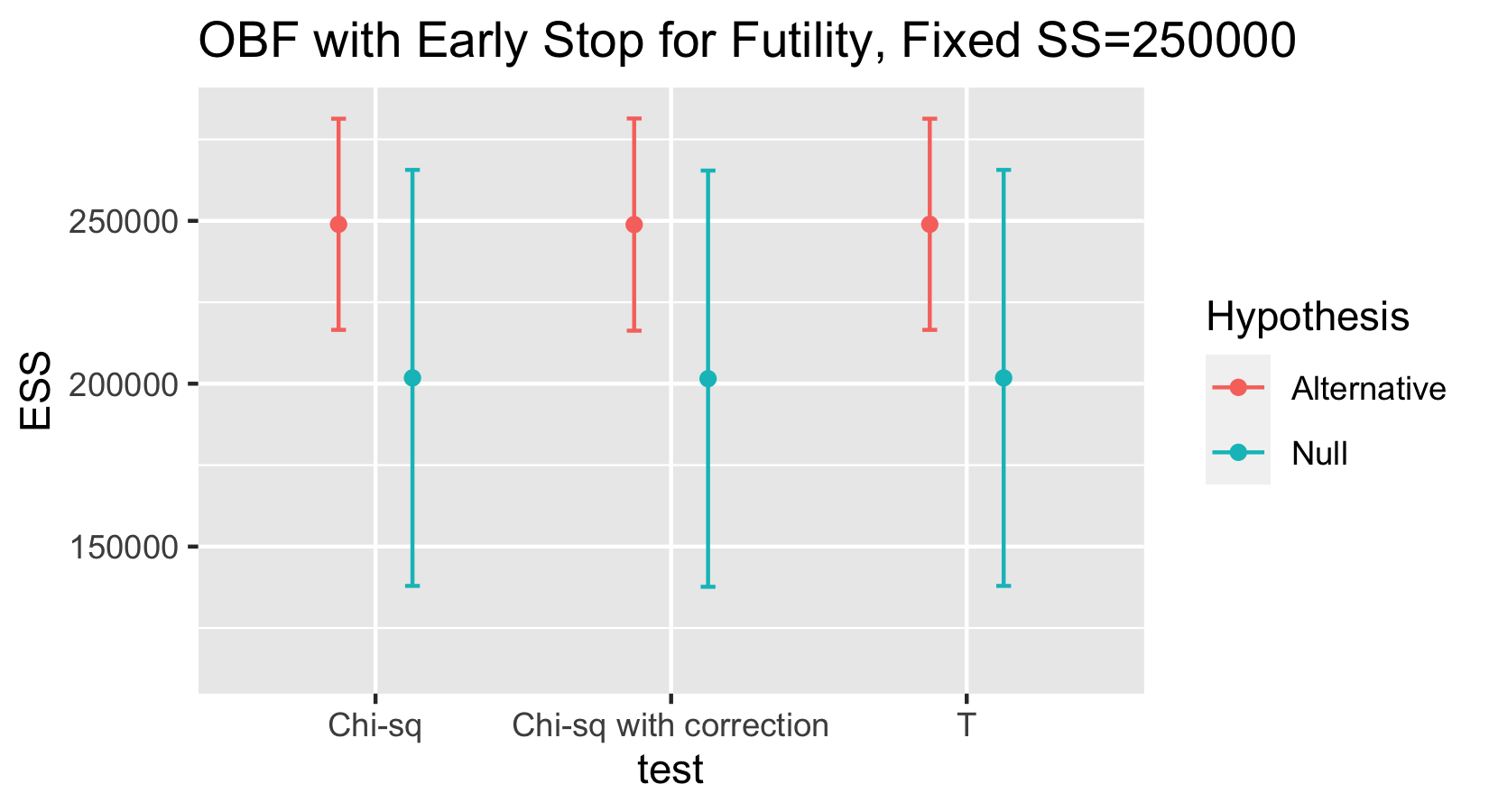


## 4-Total Looks

##
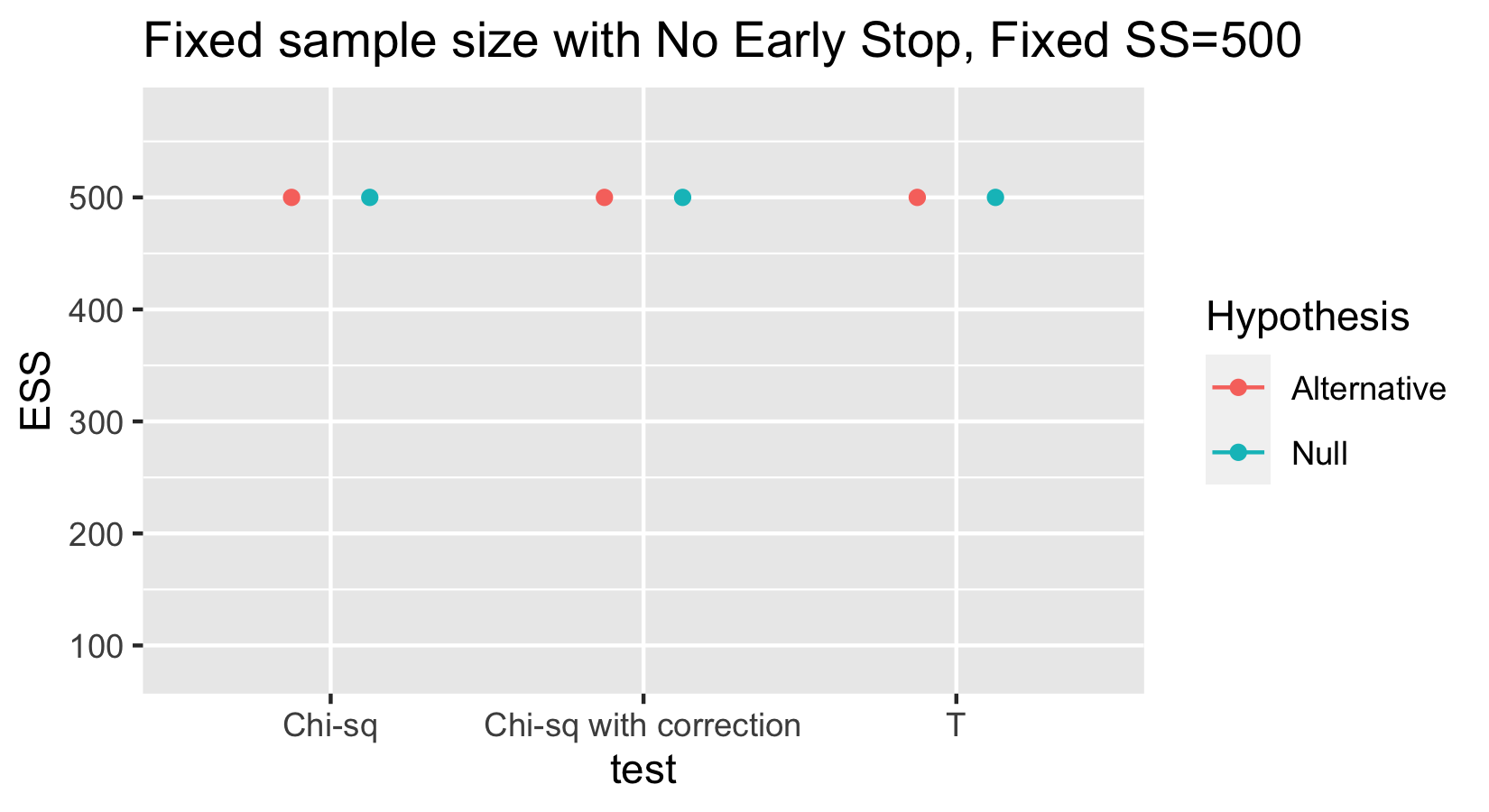

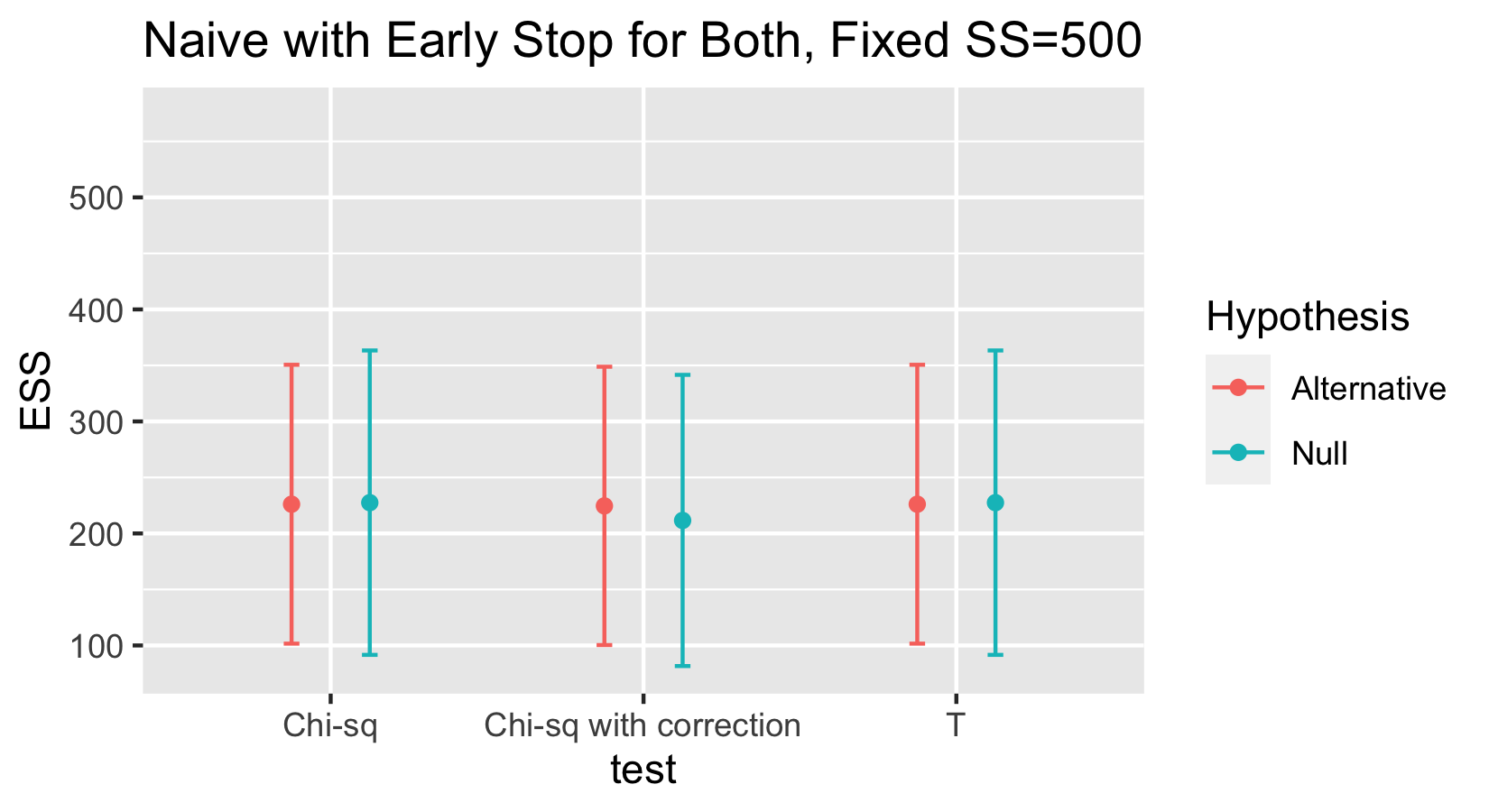

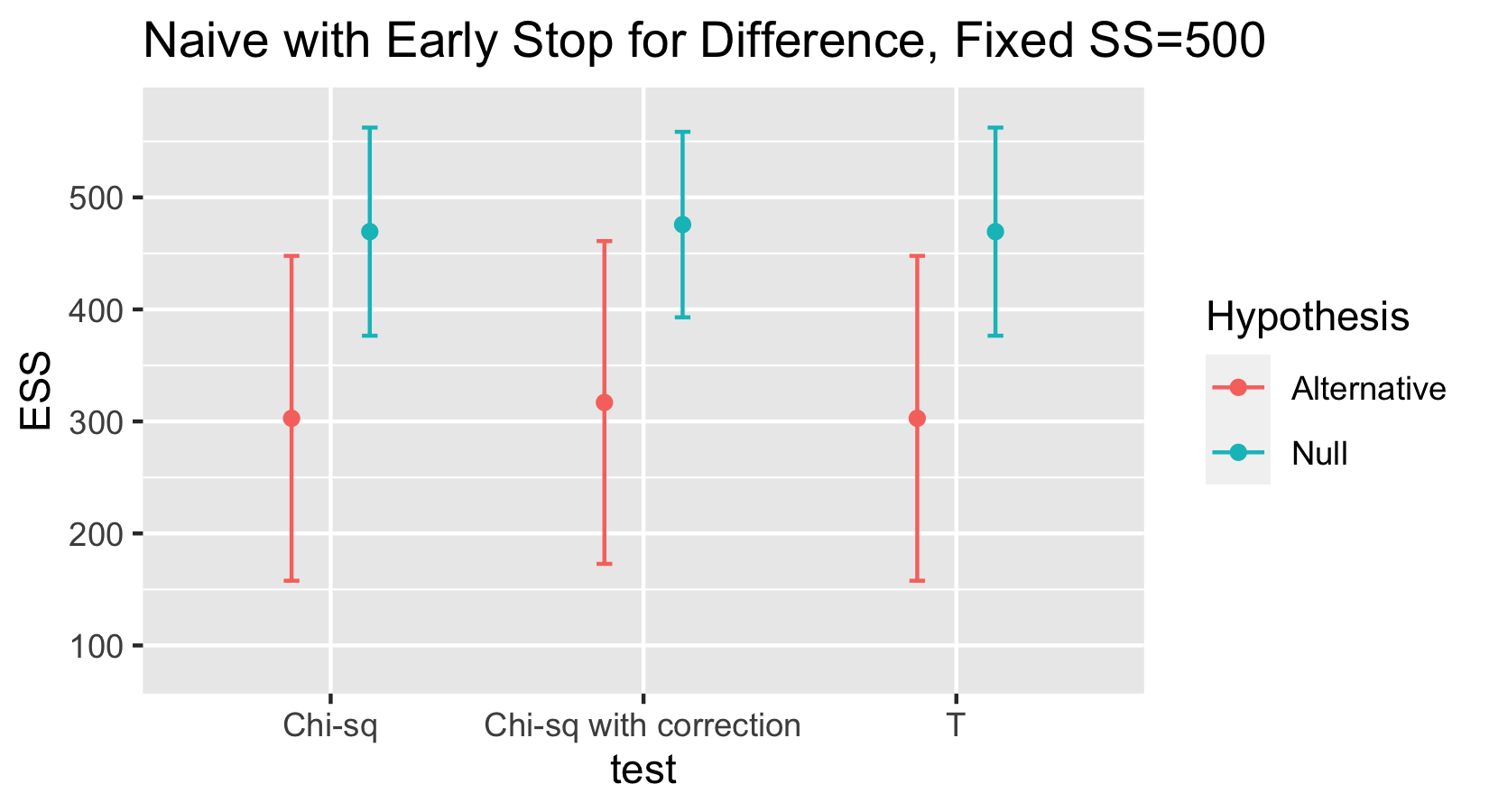


##
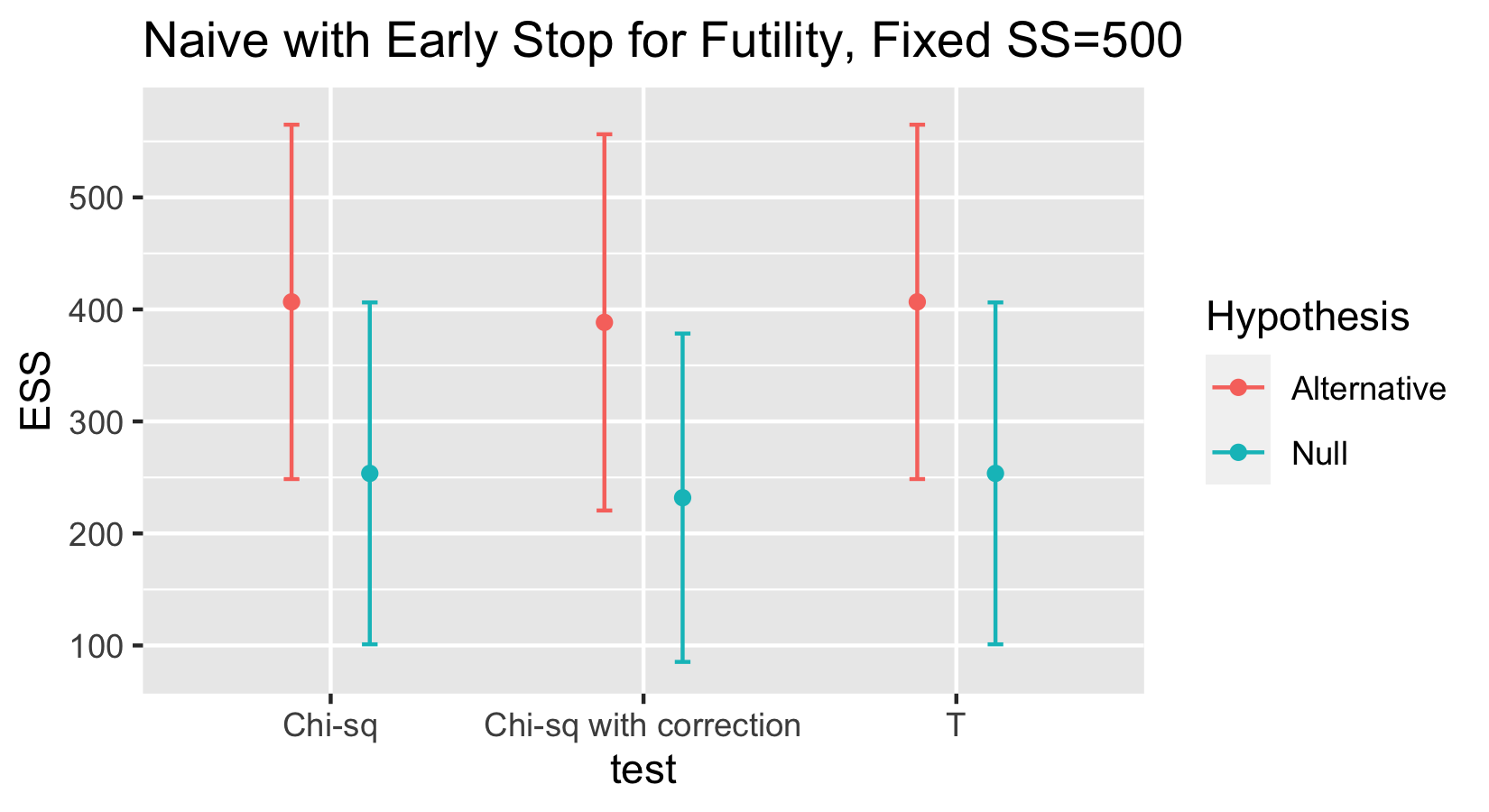

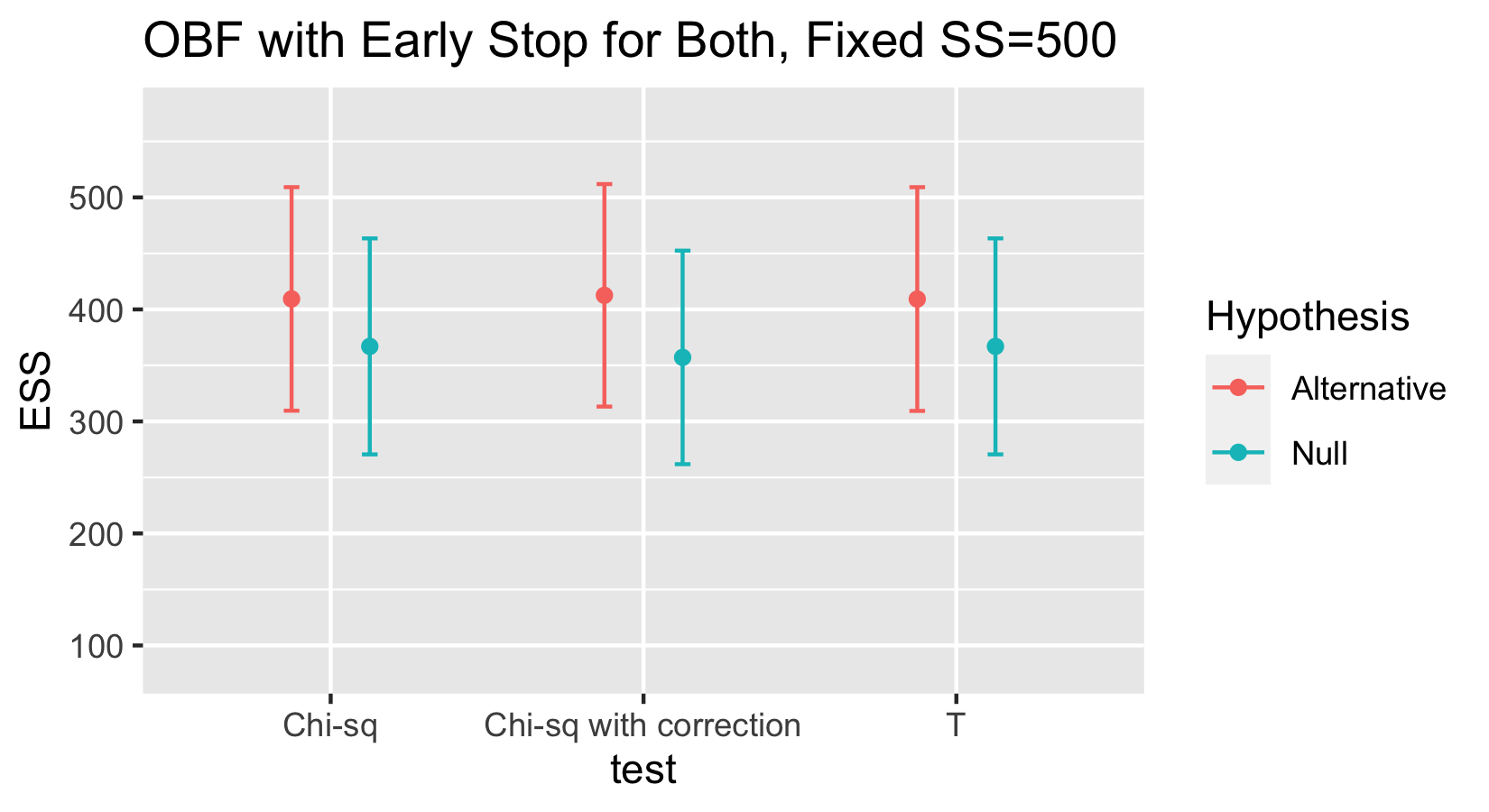

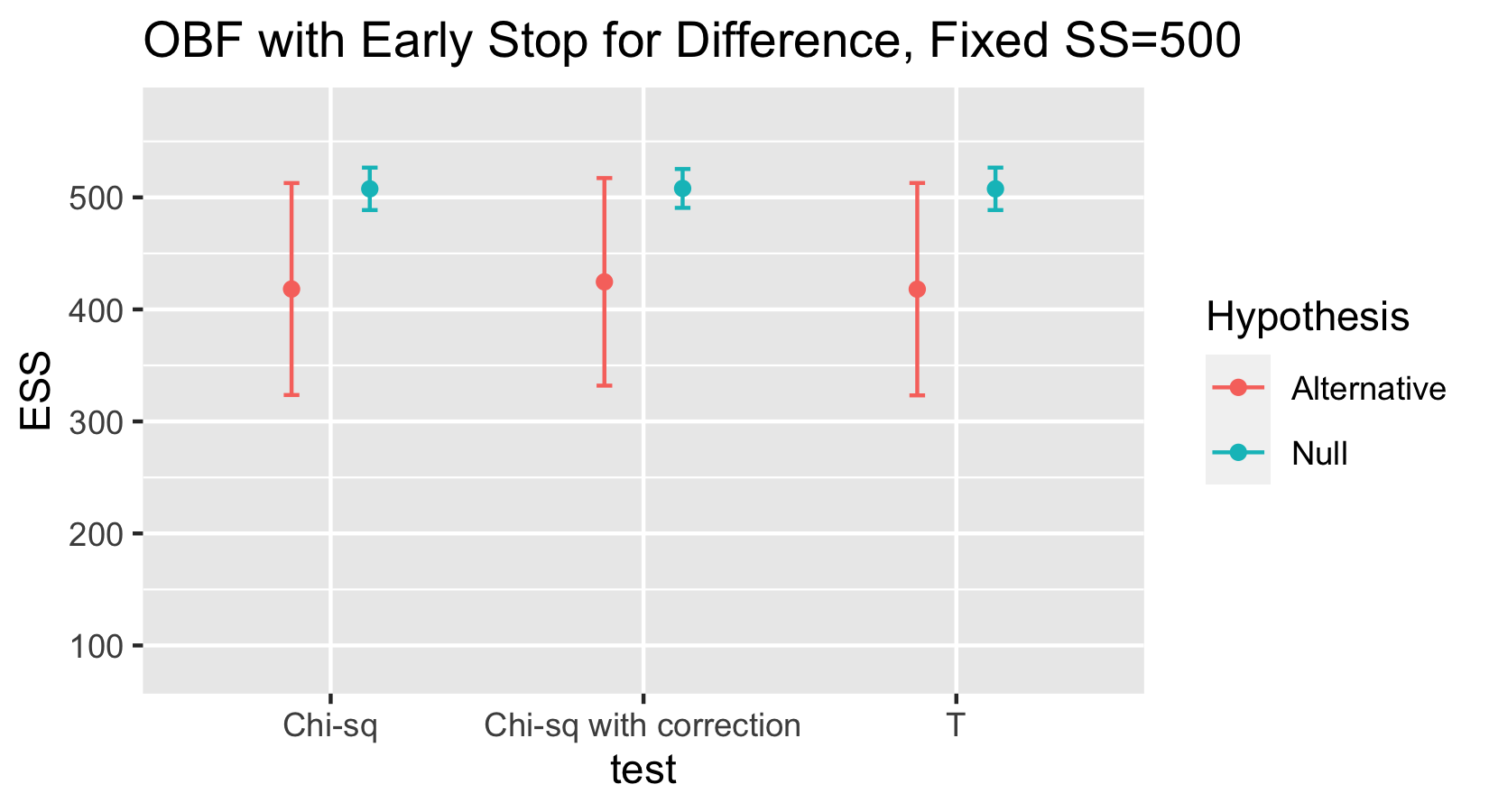


##
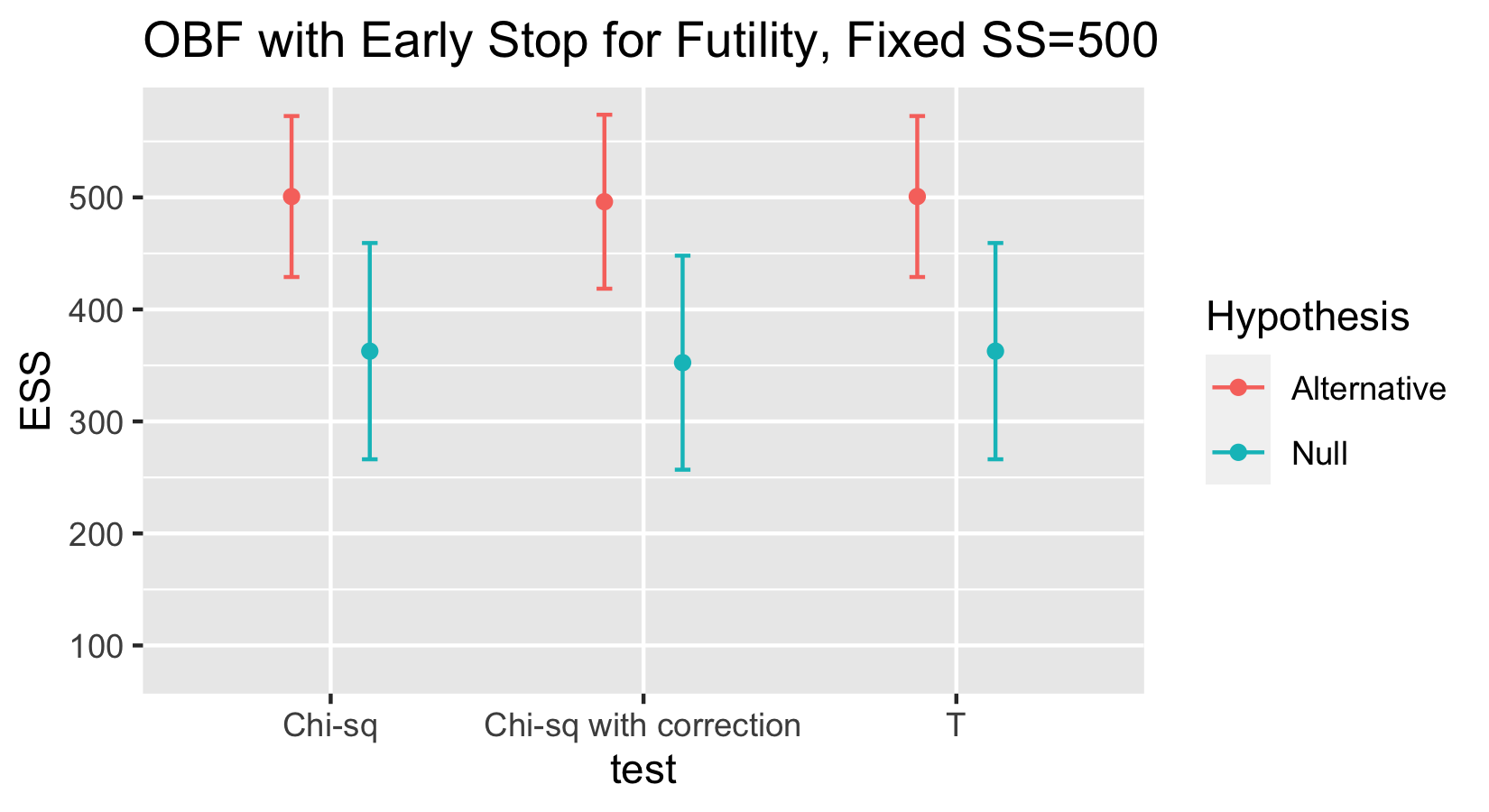


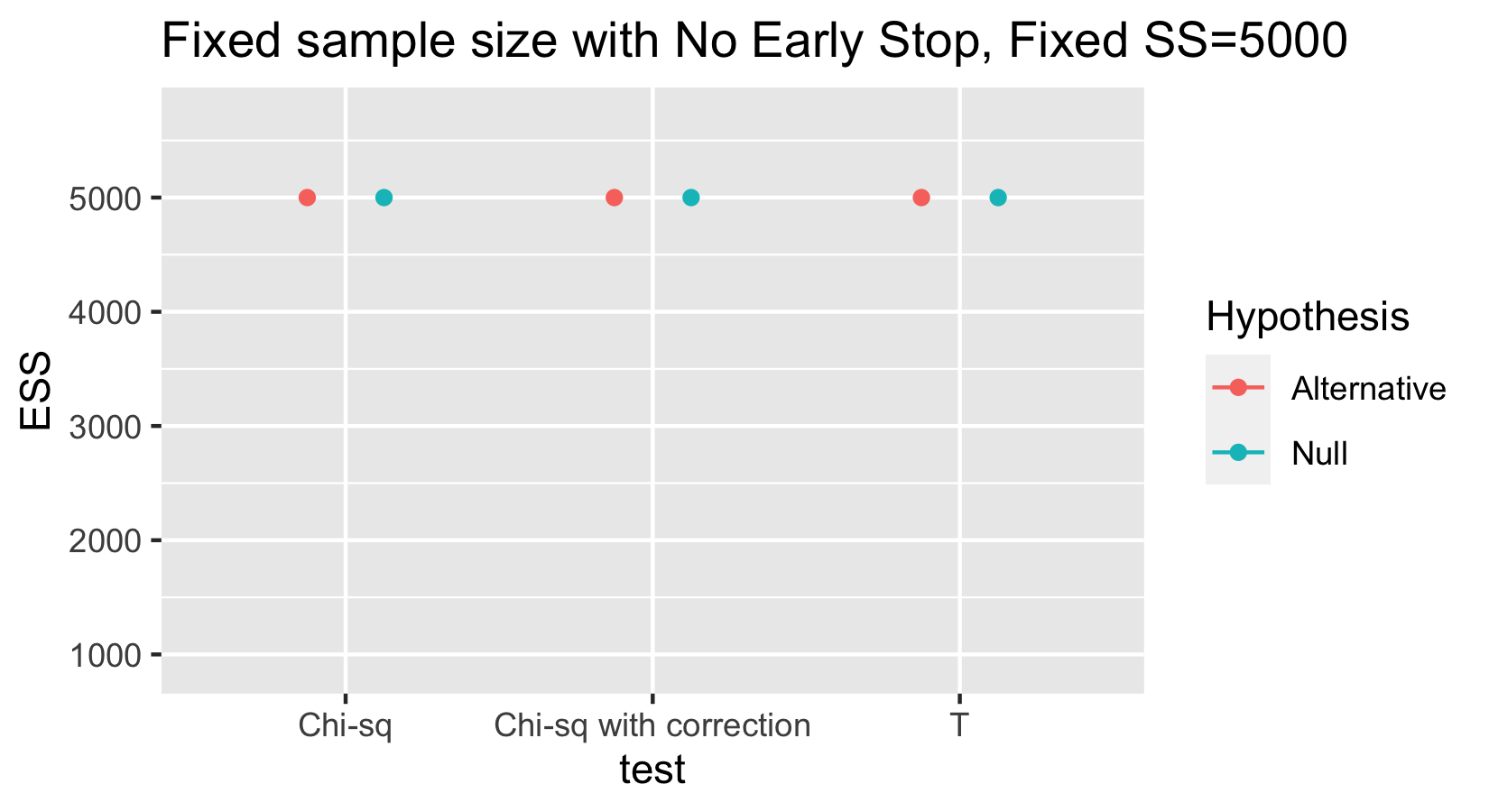

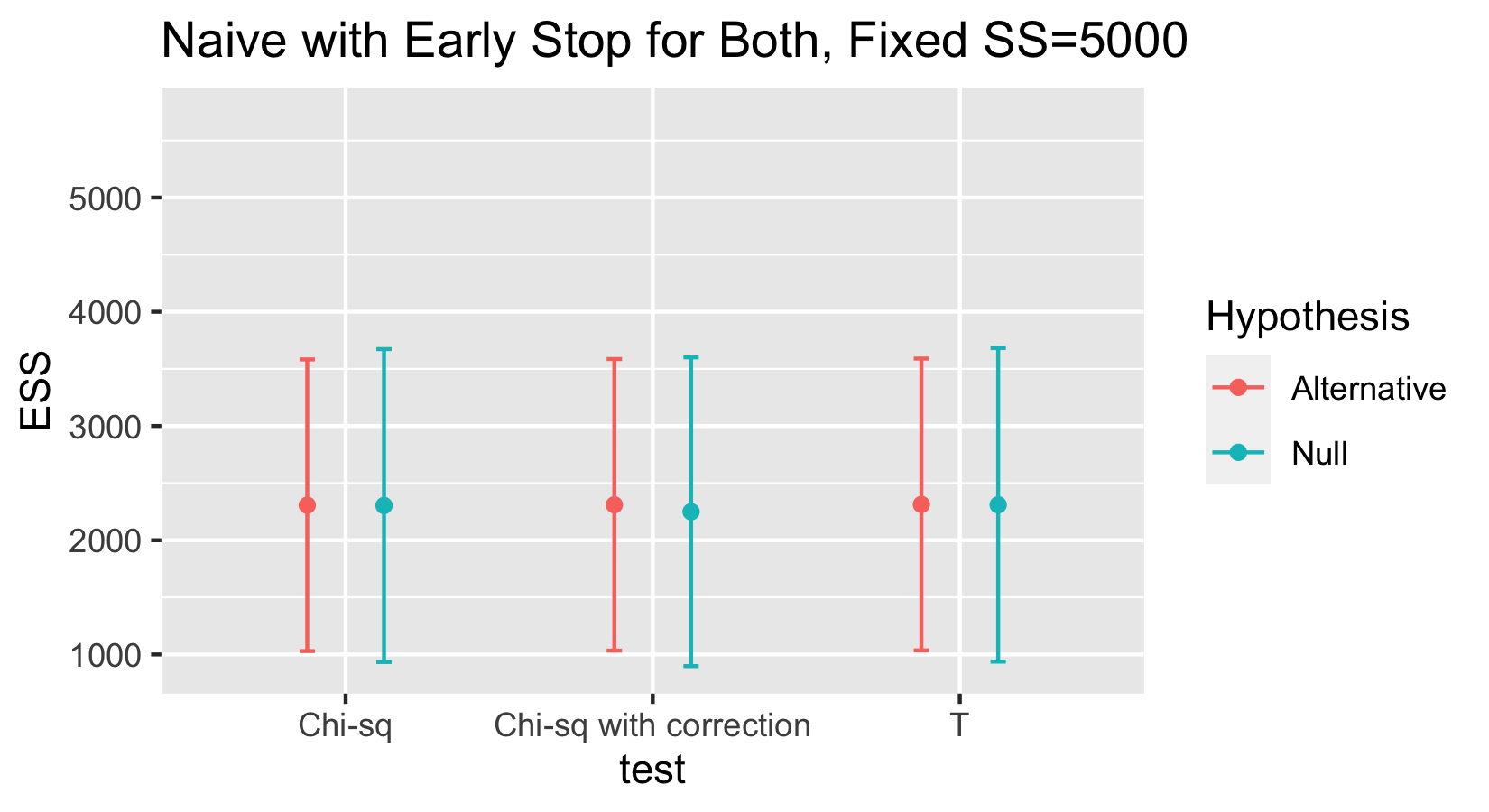


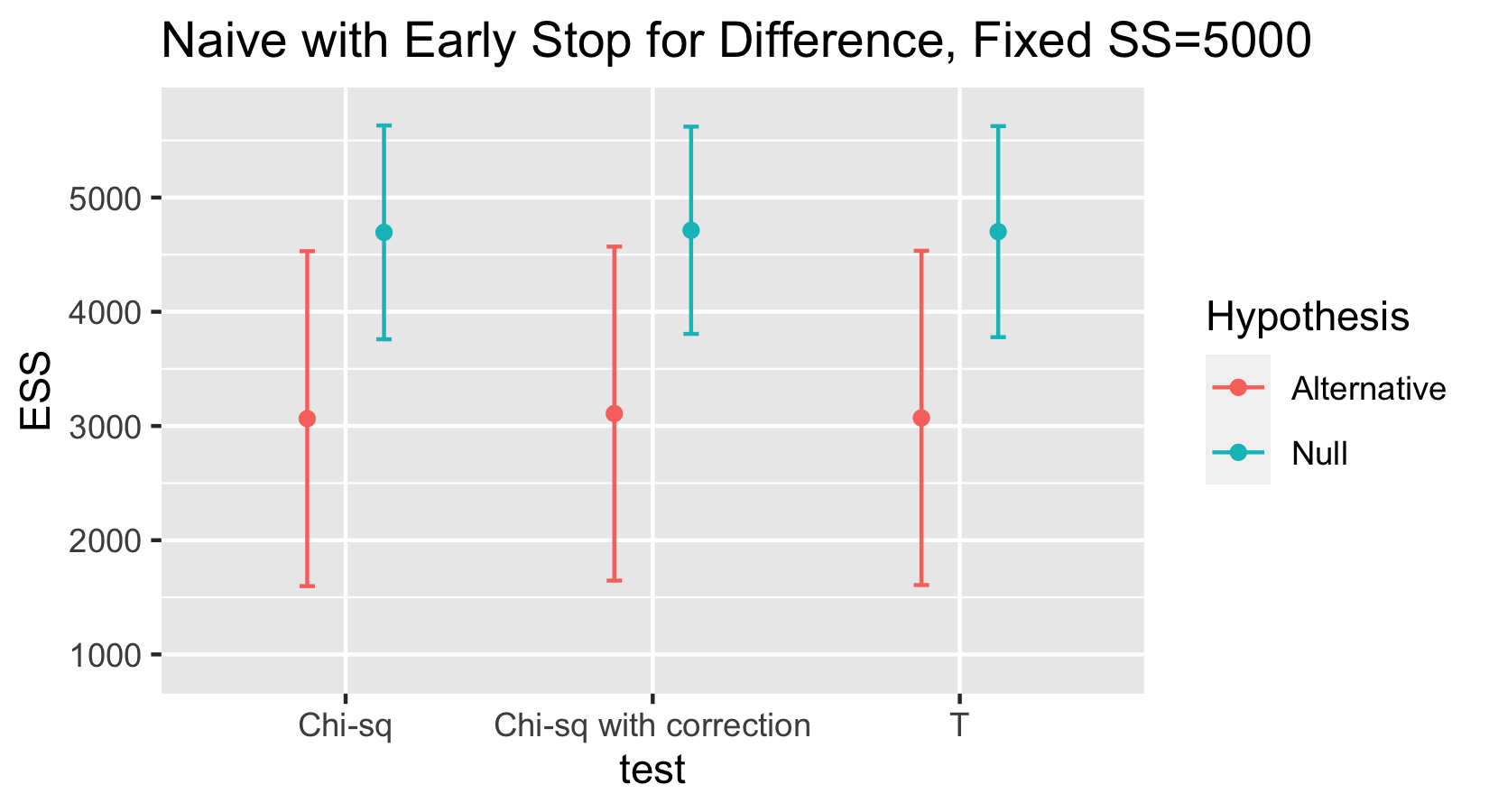

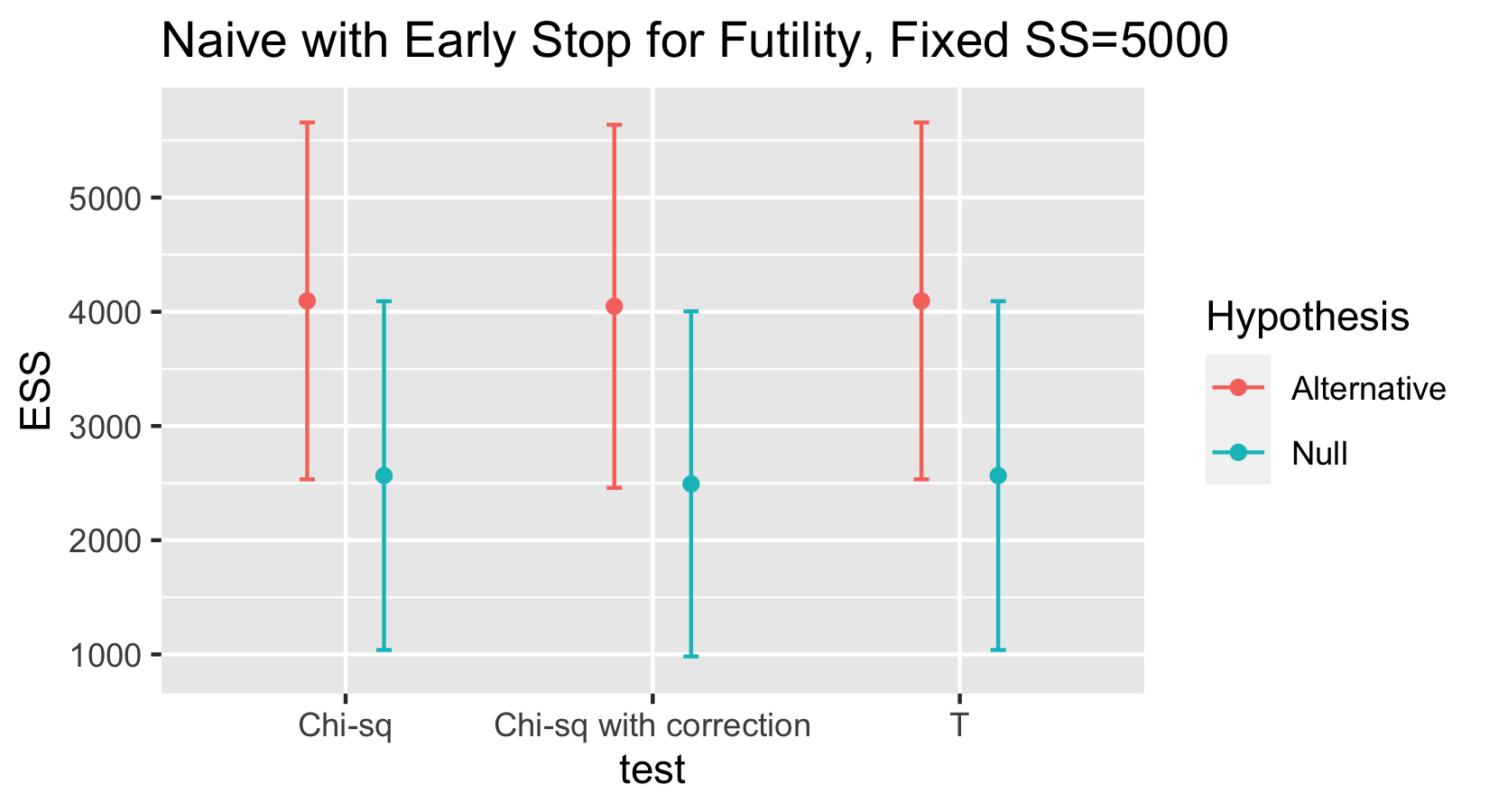

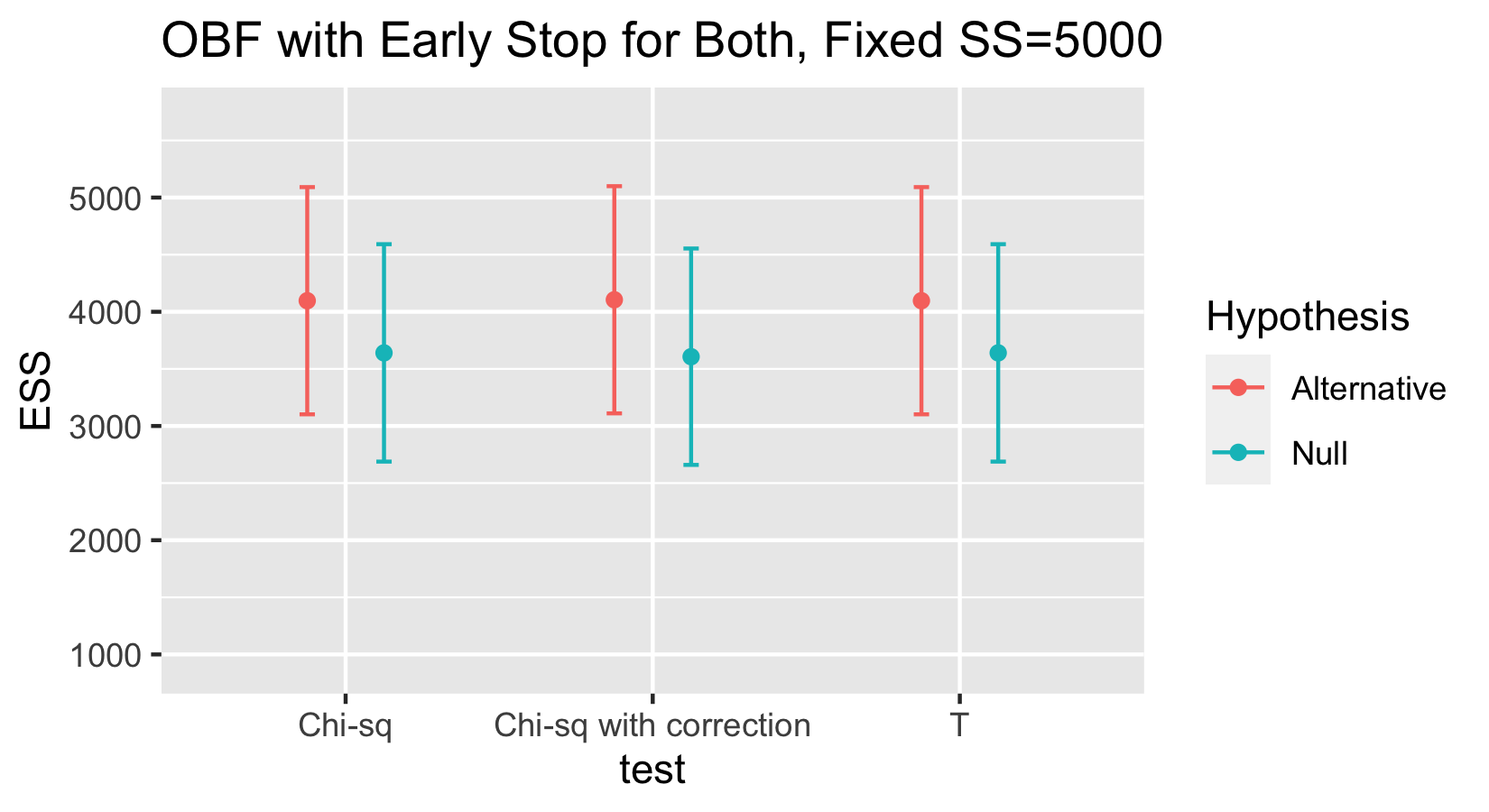


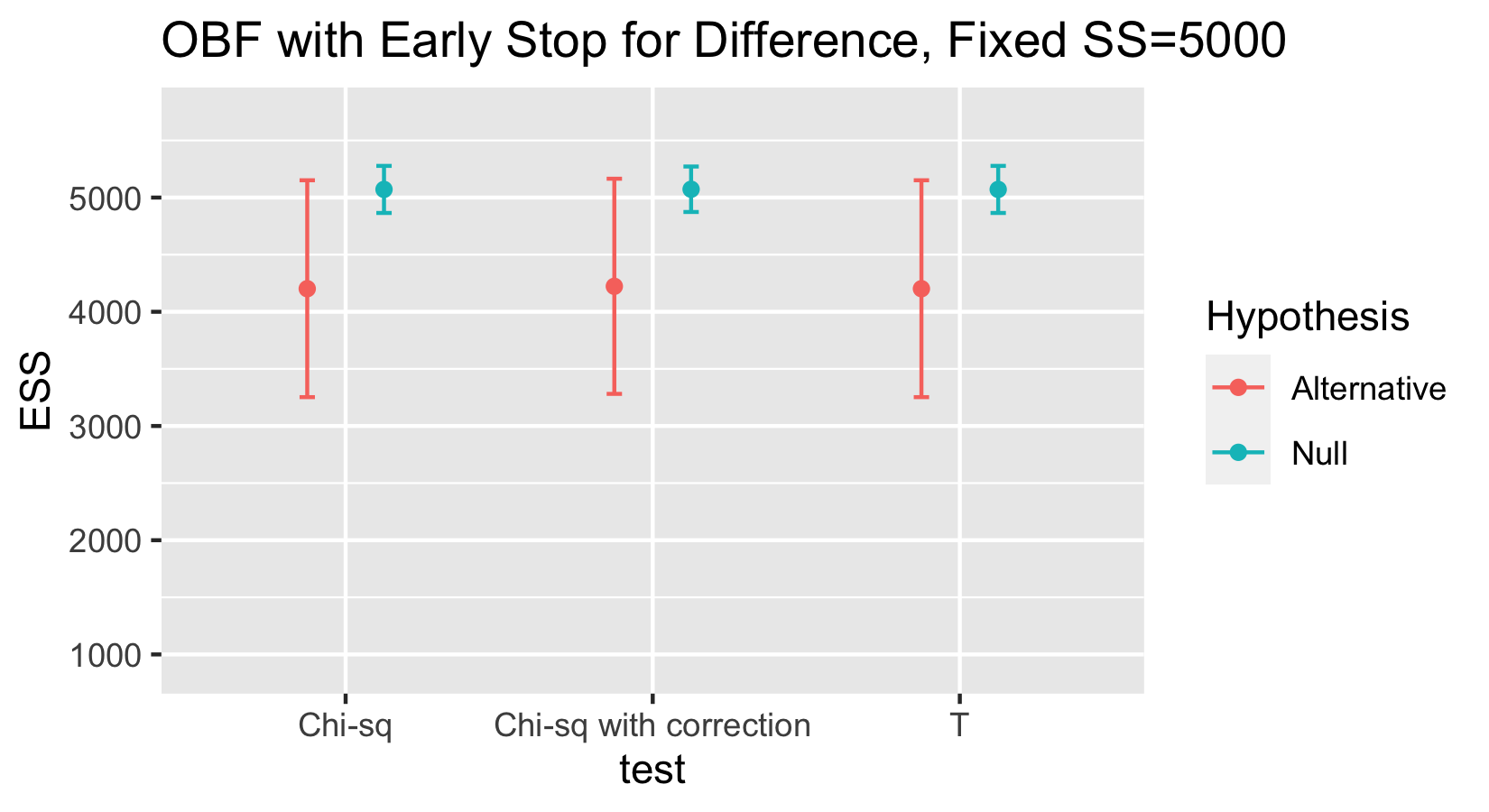

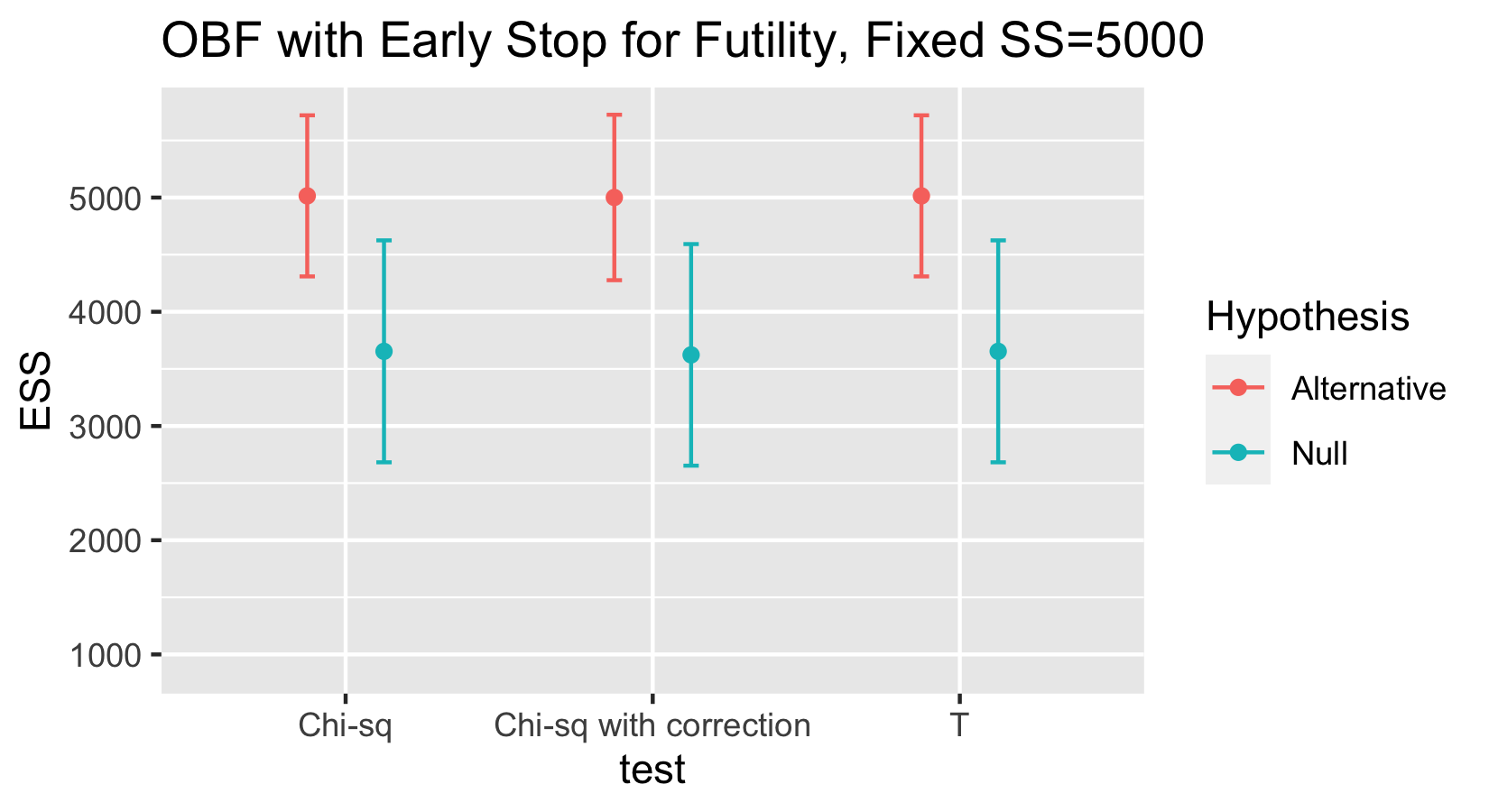


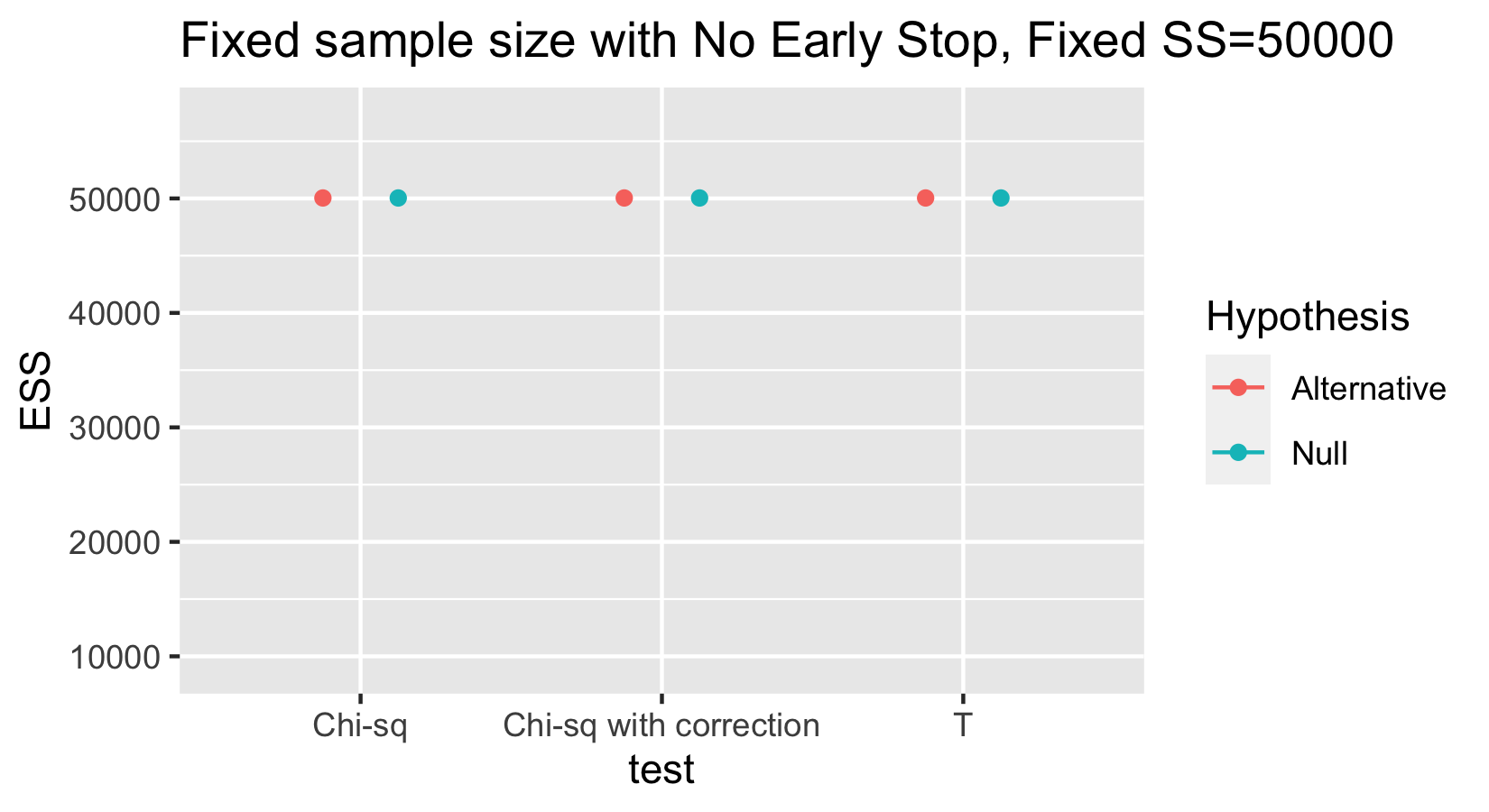


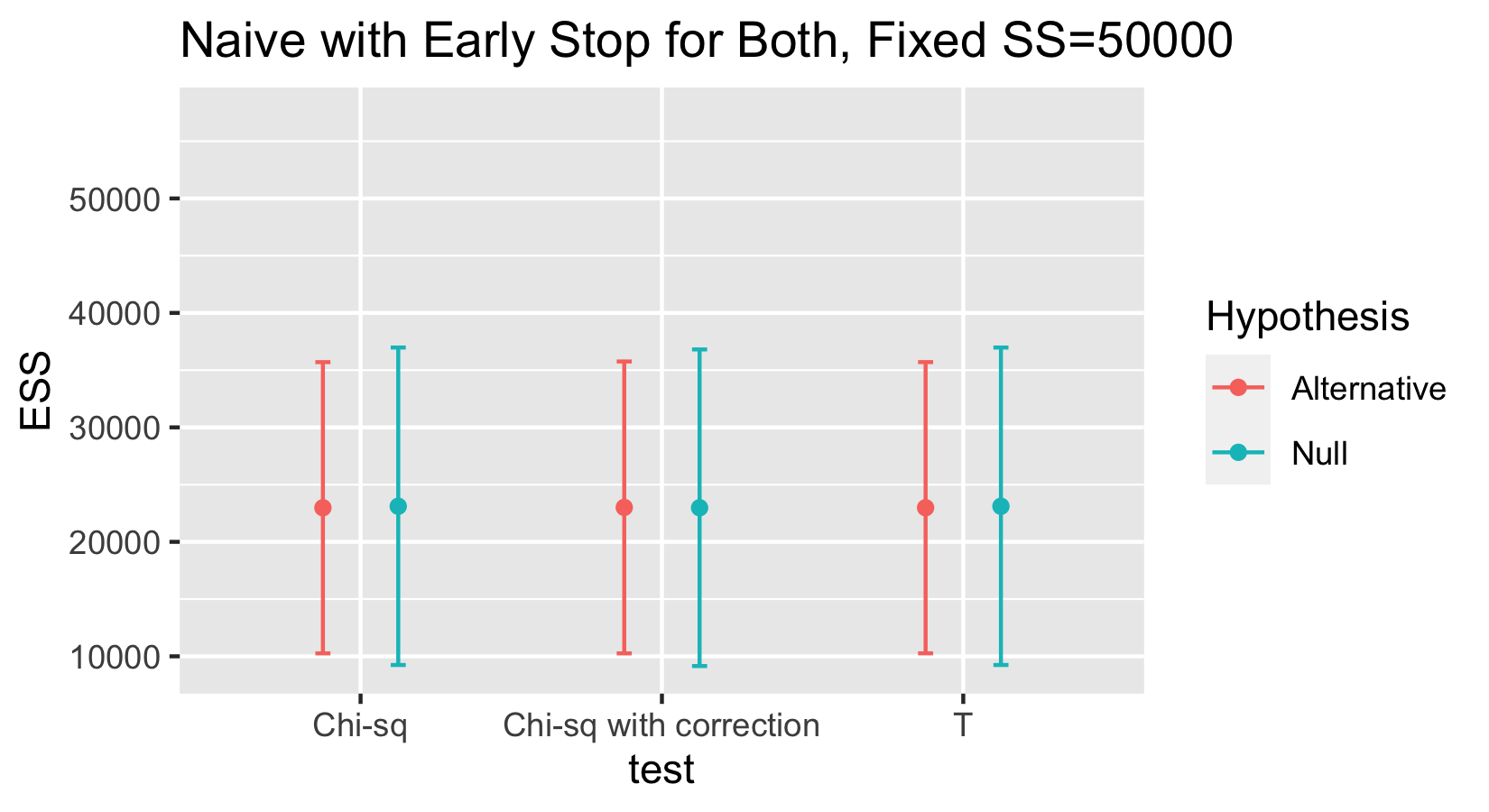

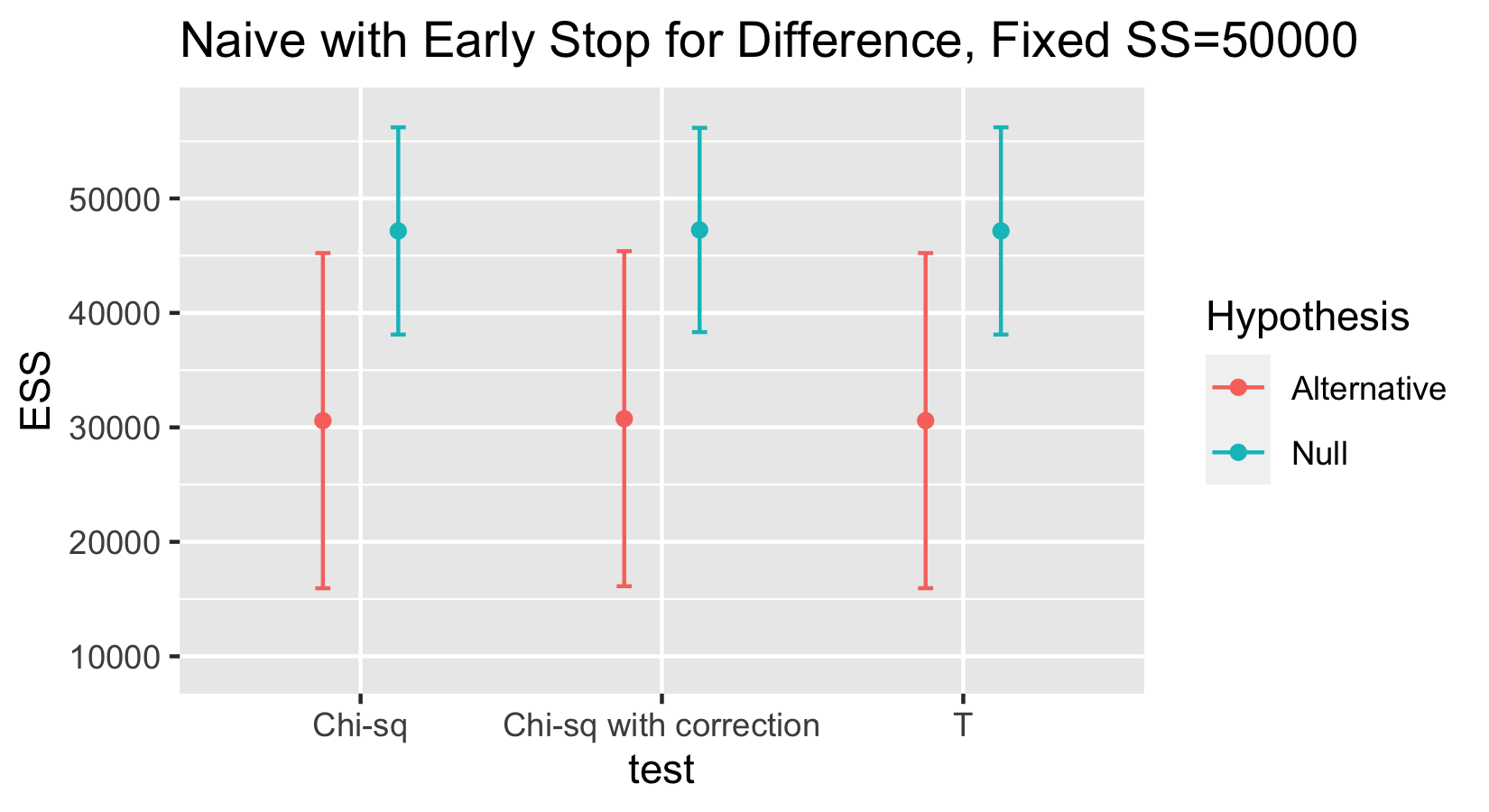

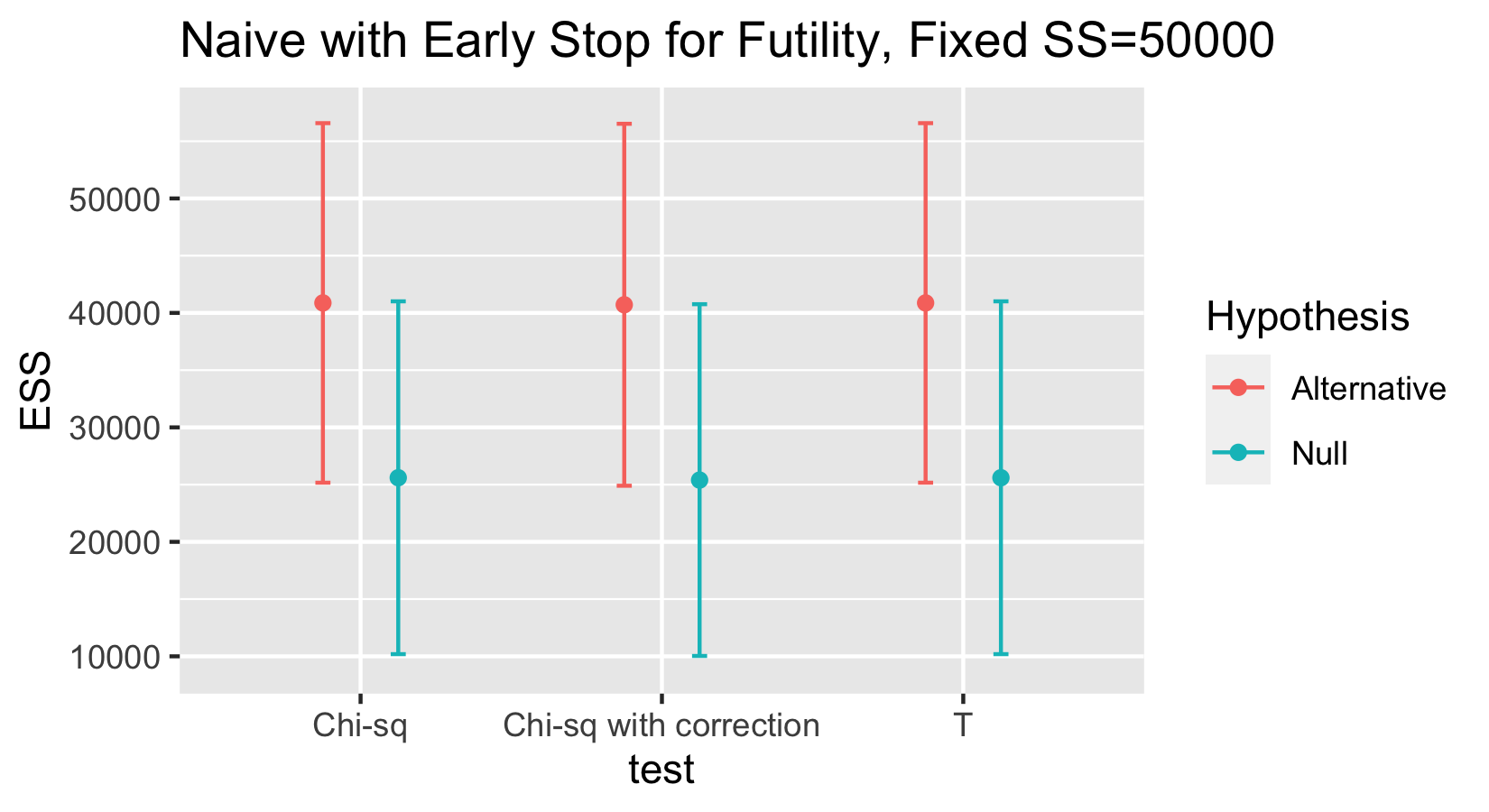


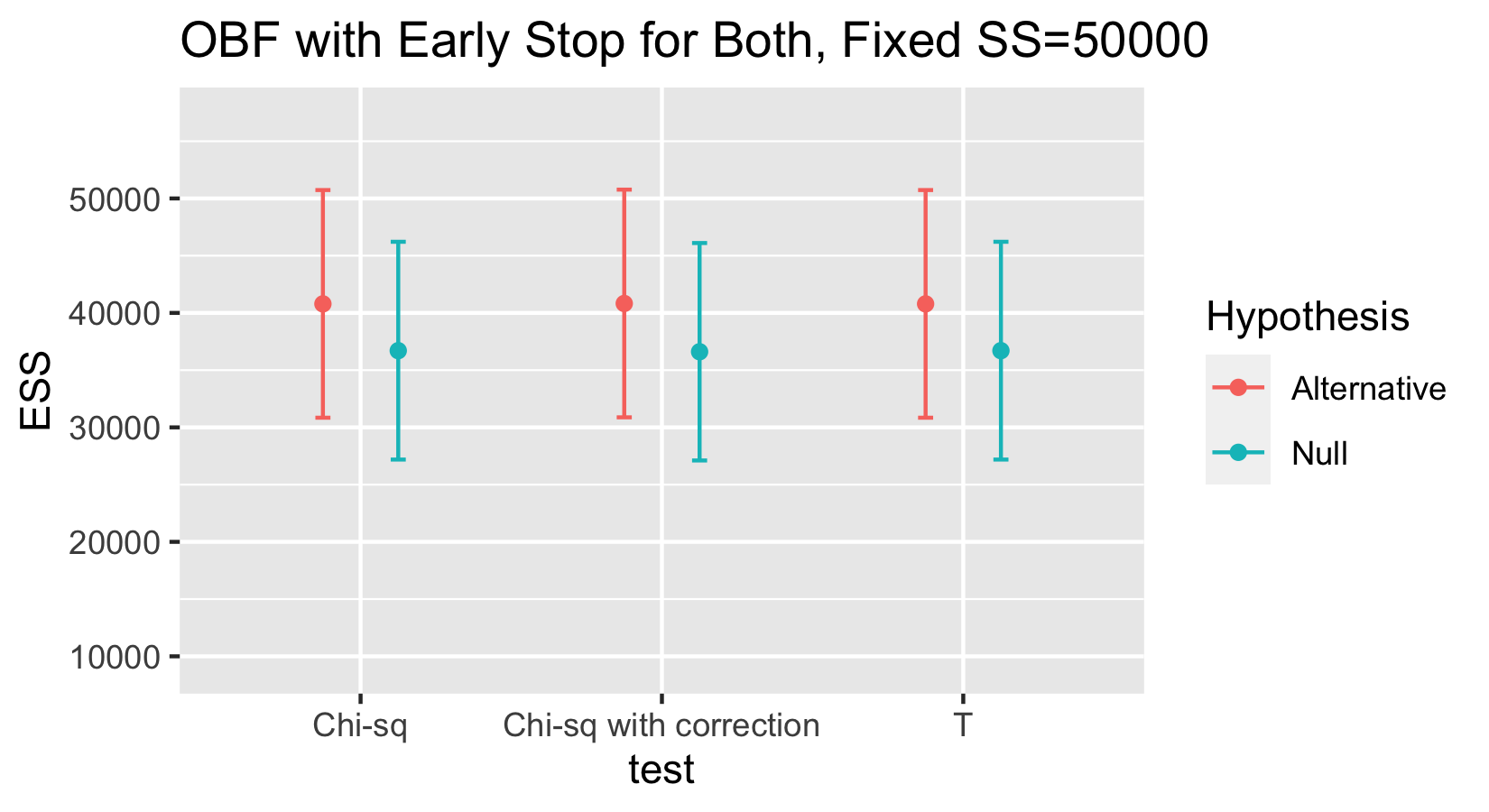

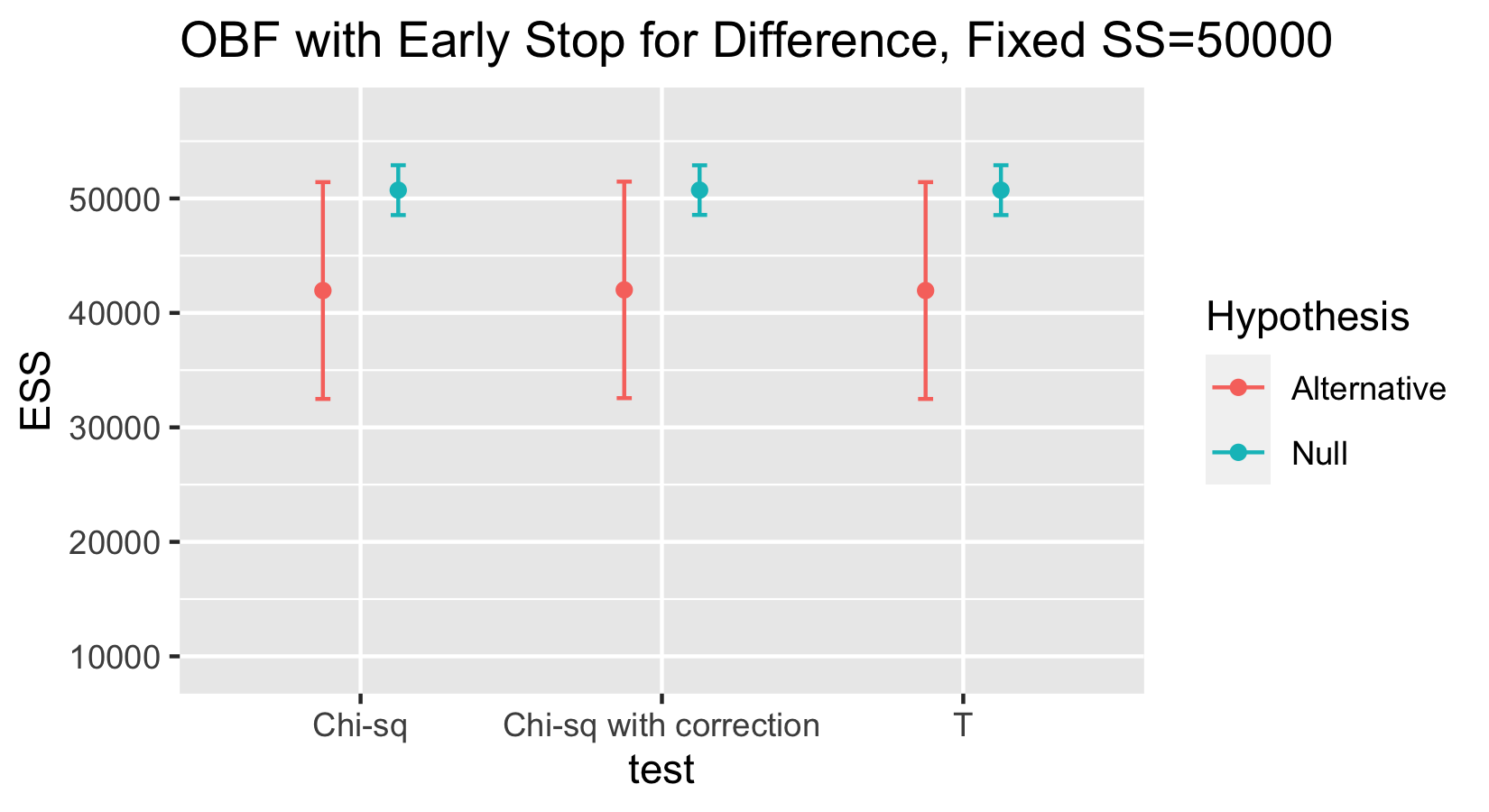

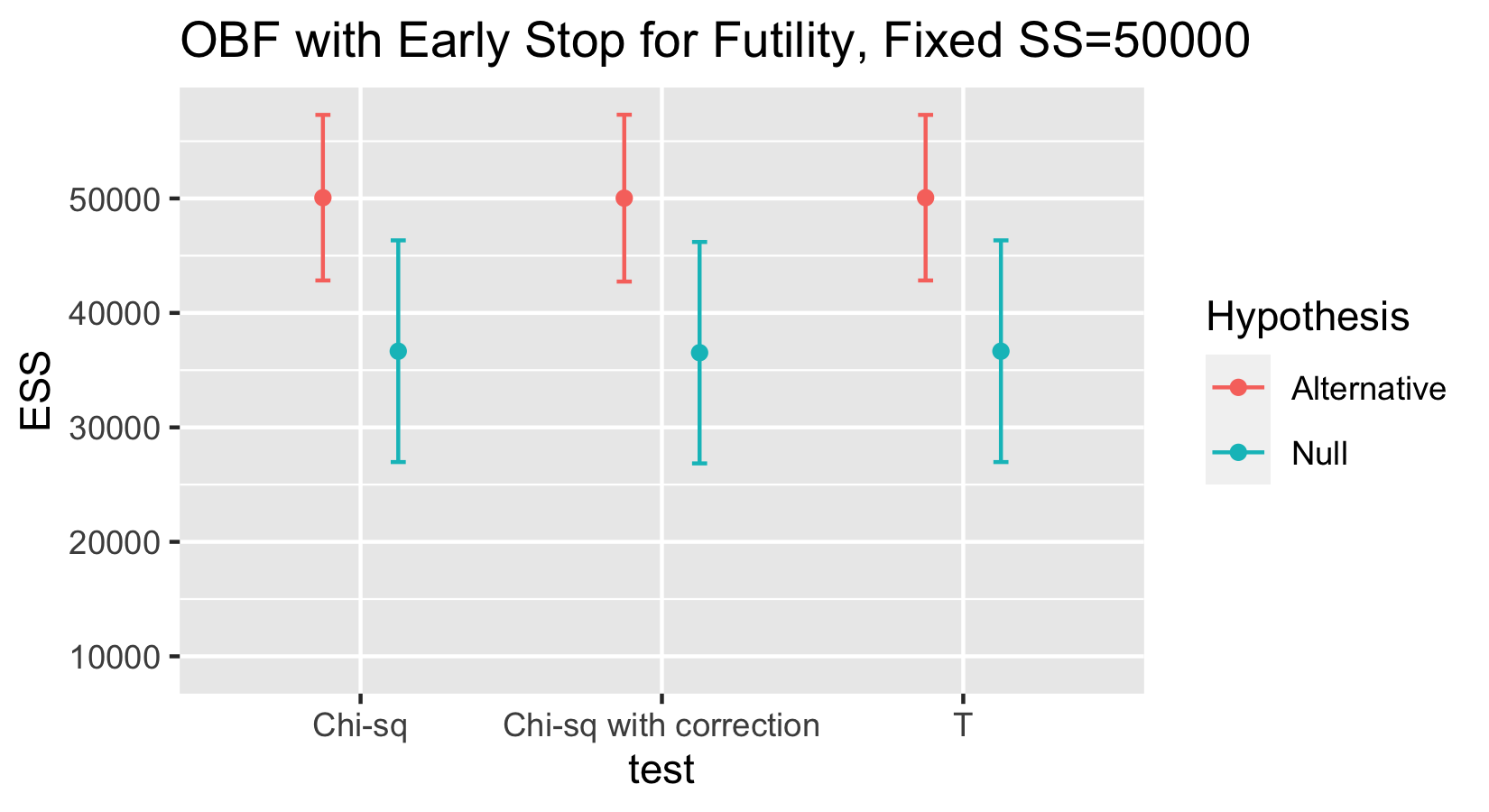


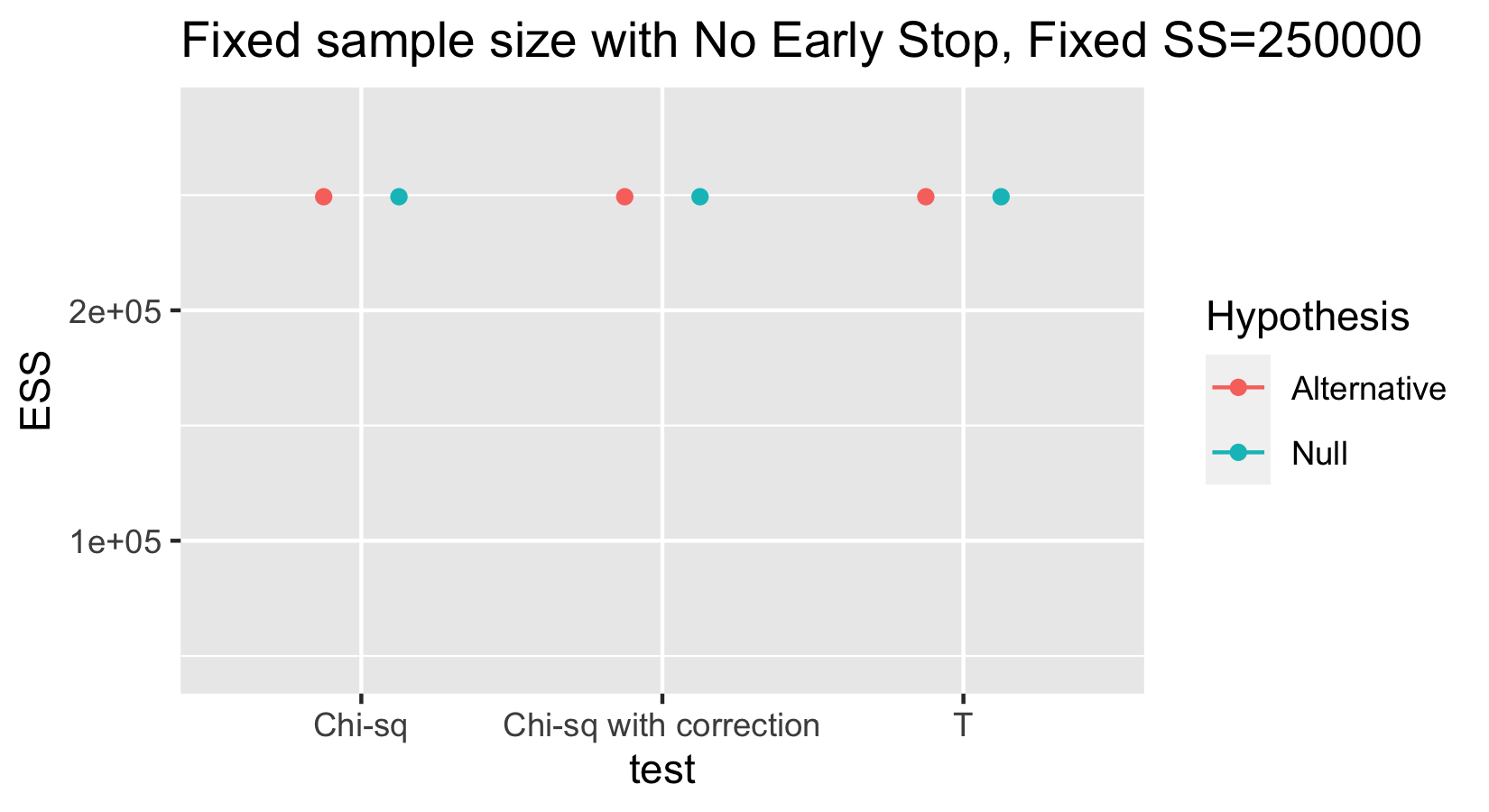

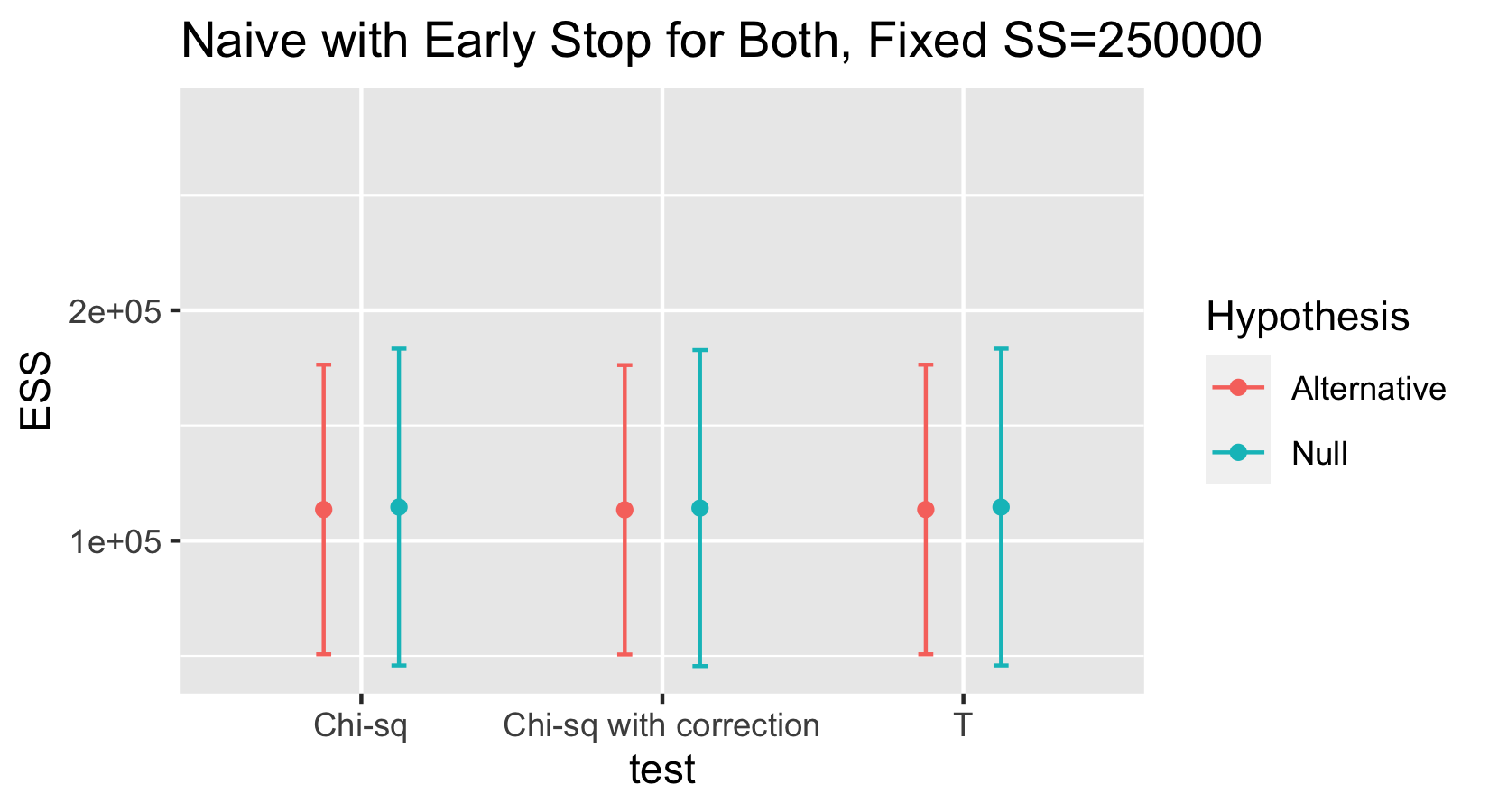

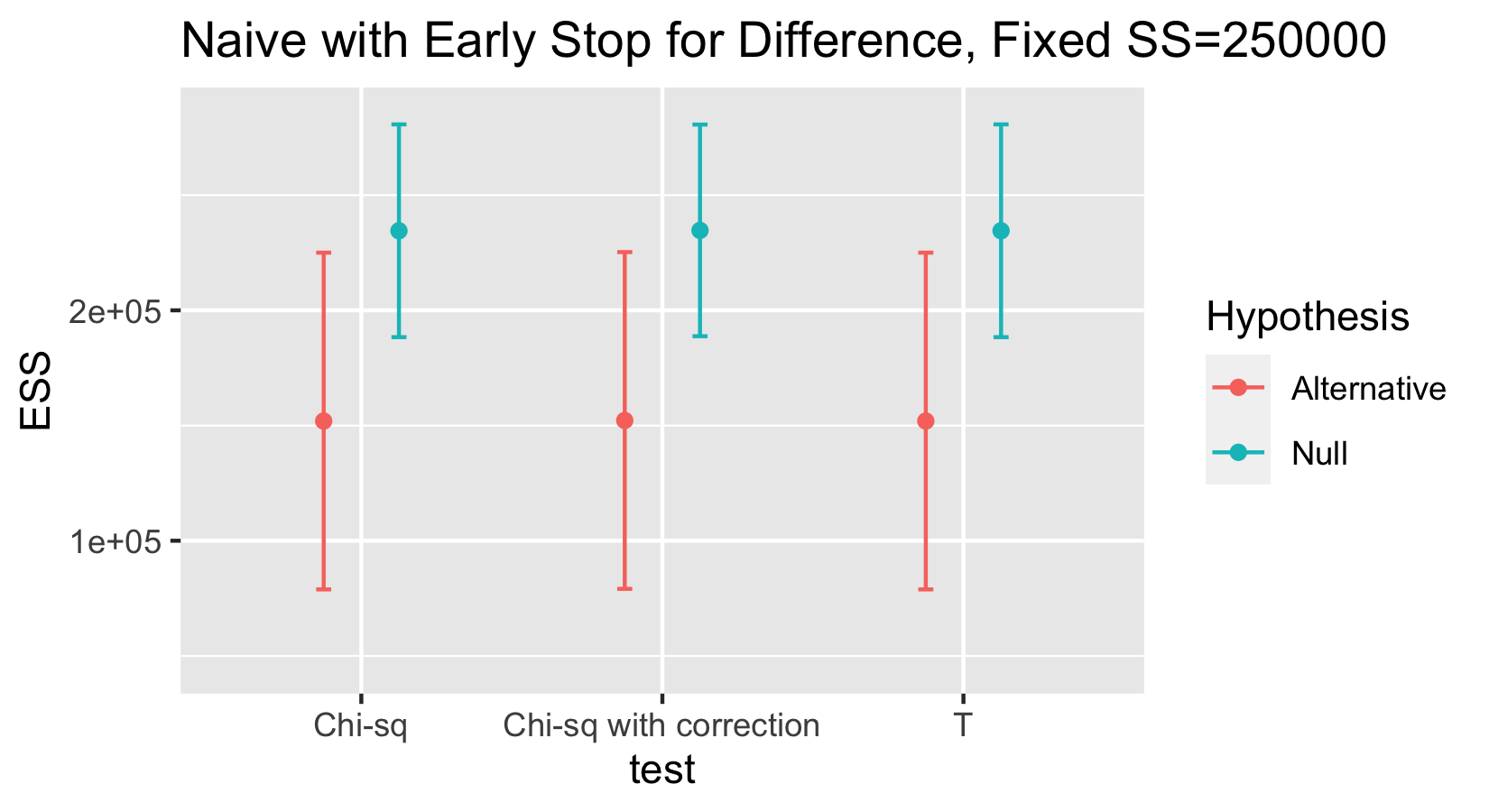


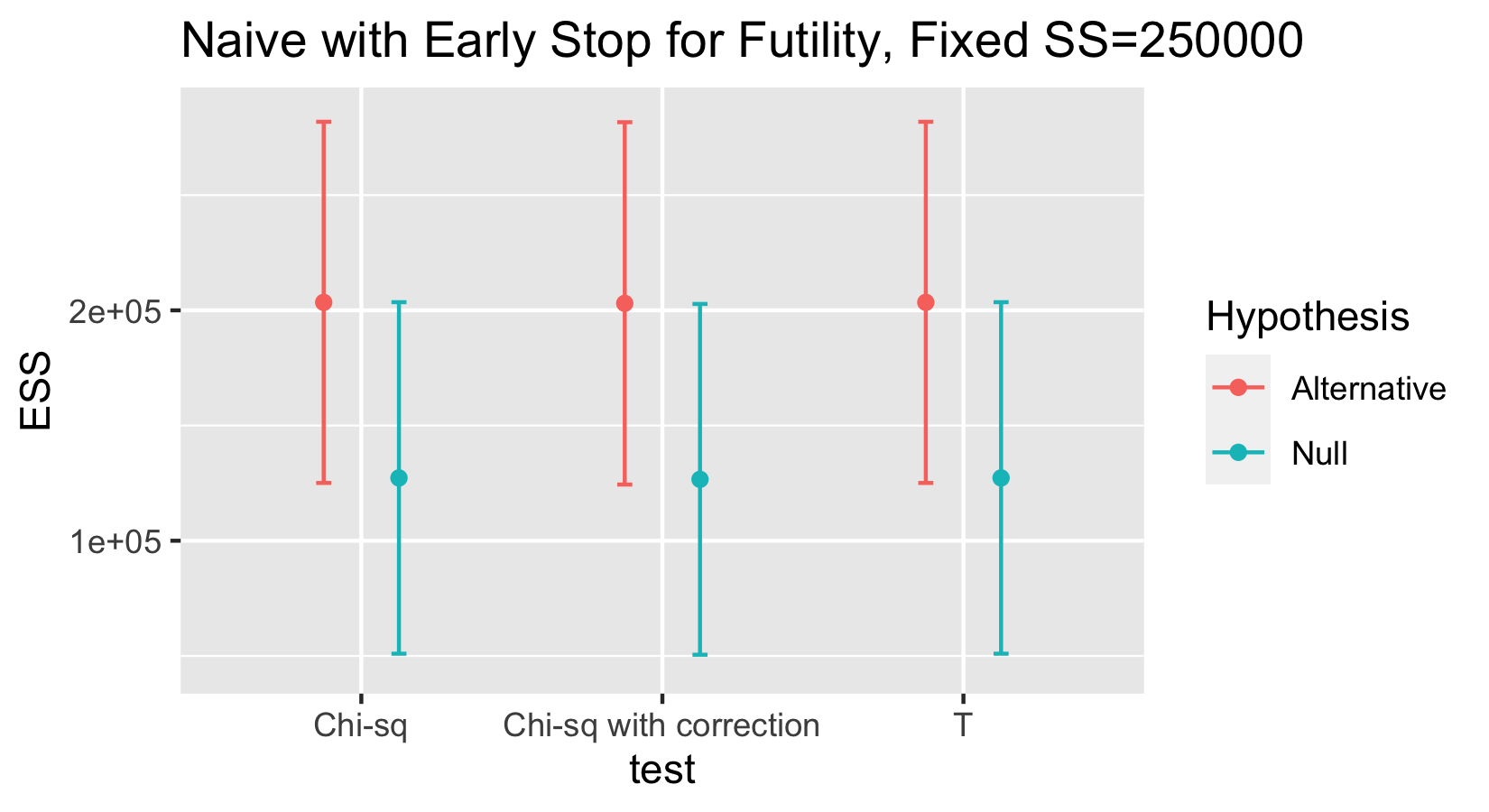

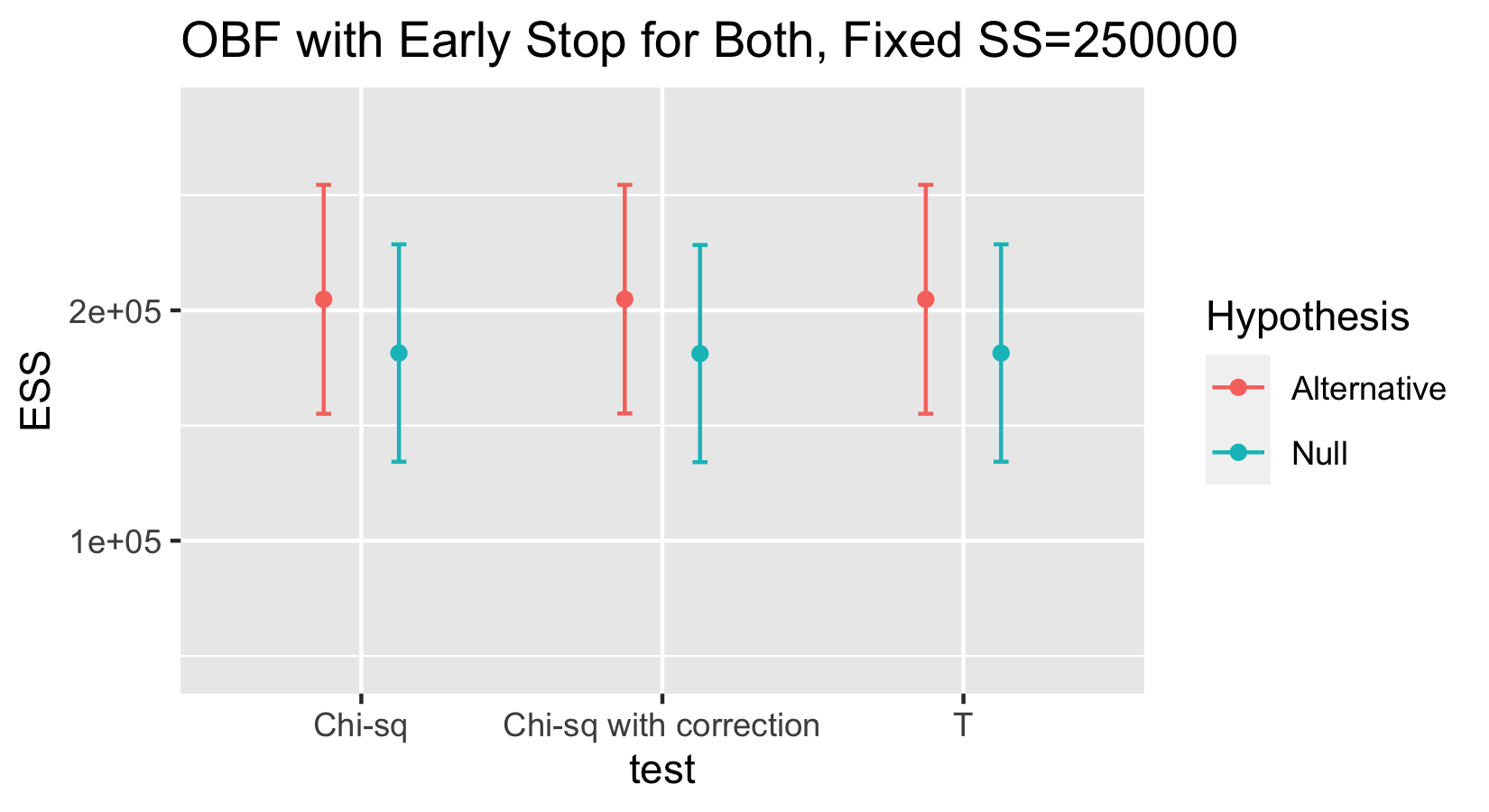

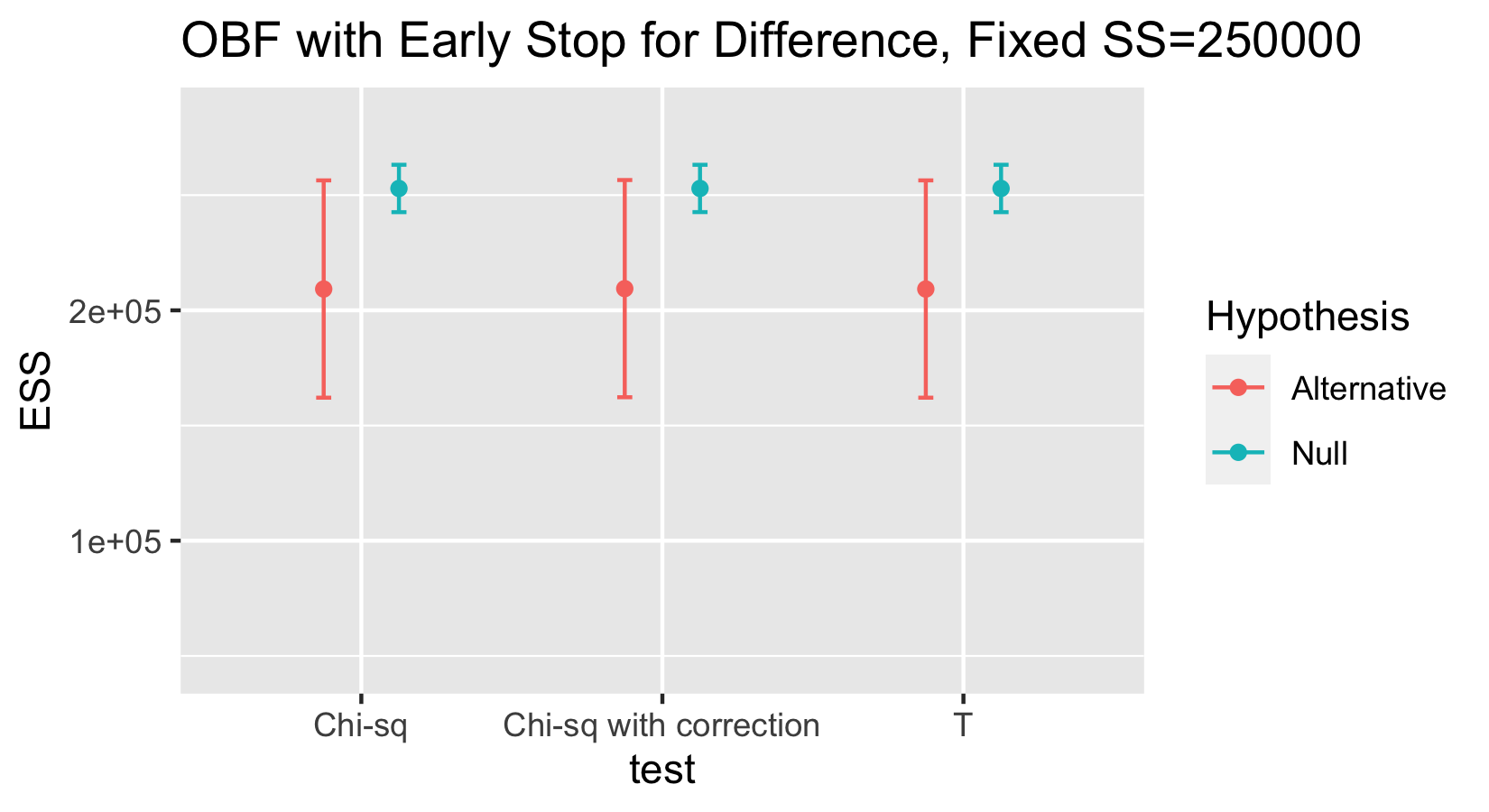


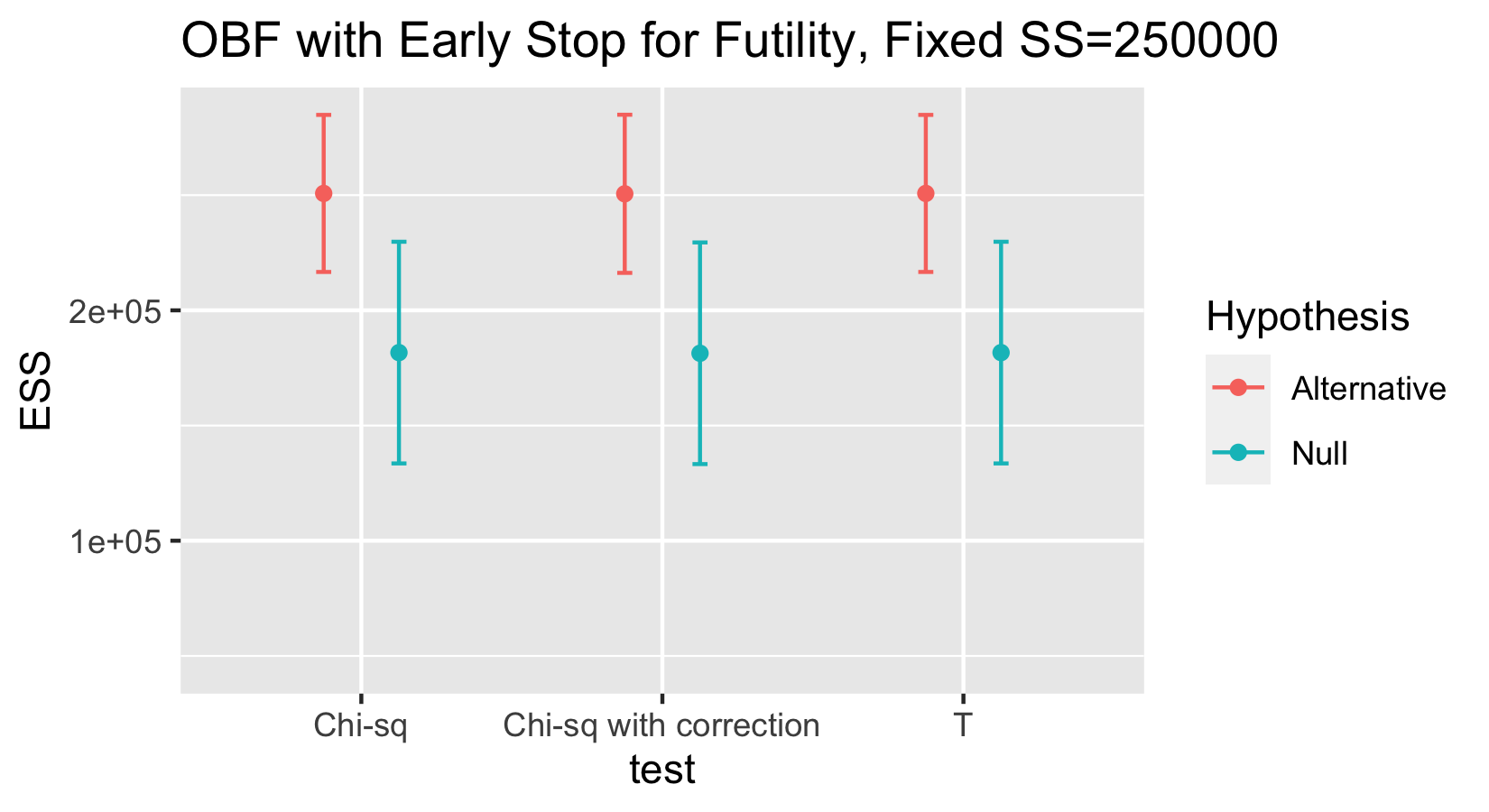


## 20-Total Looks

##
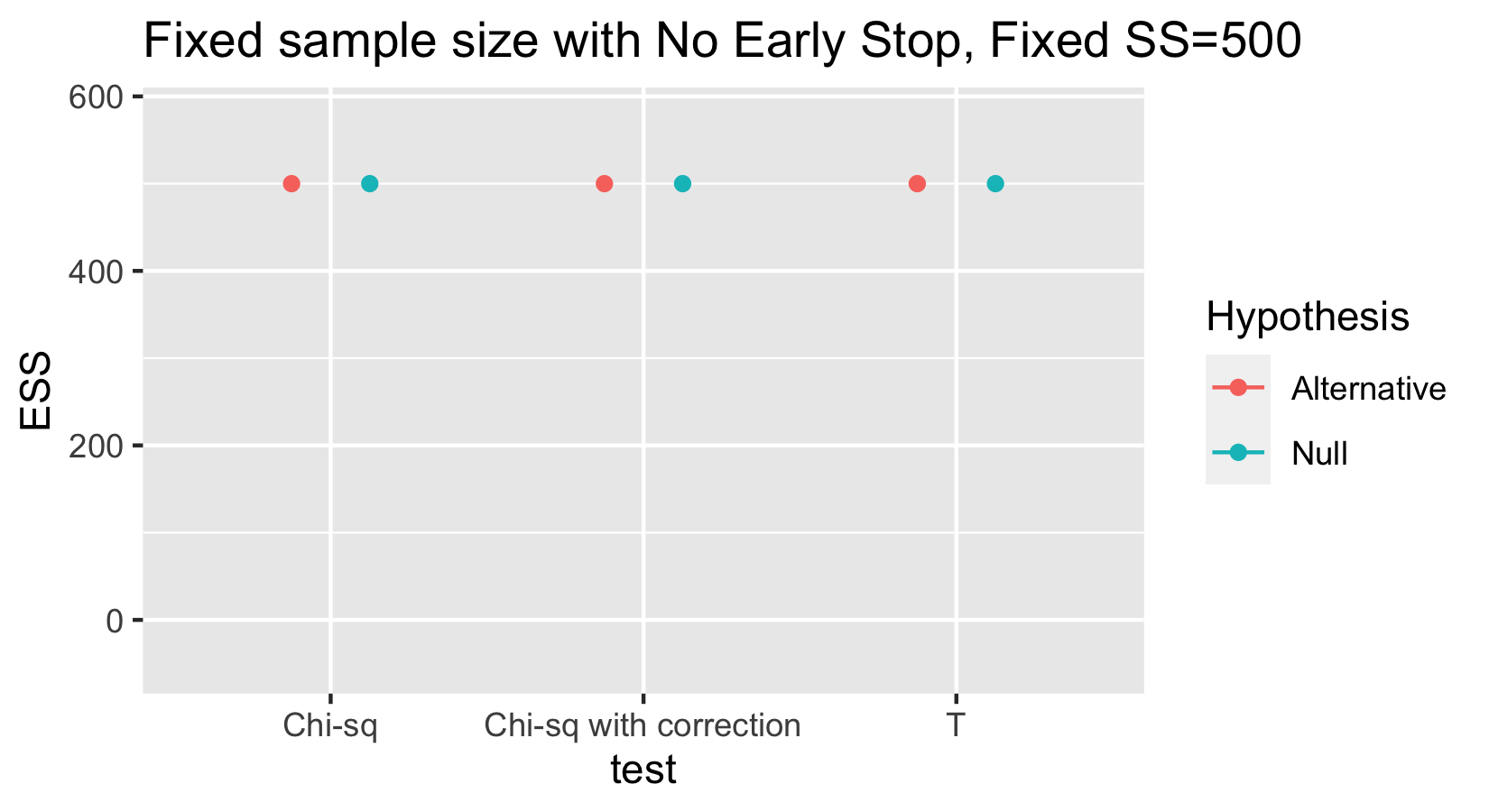

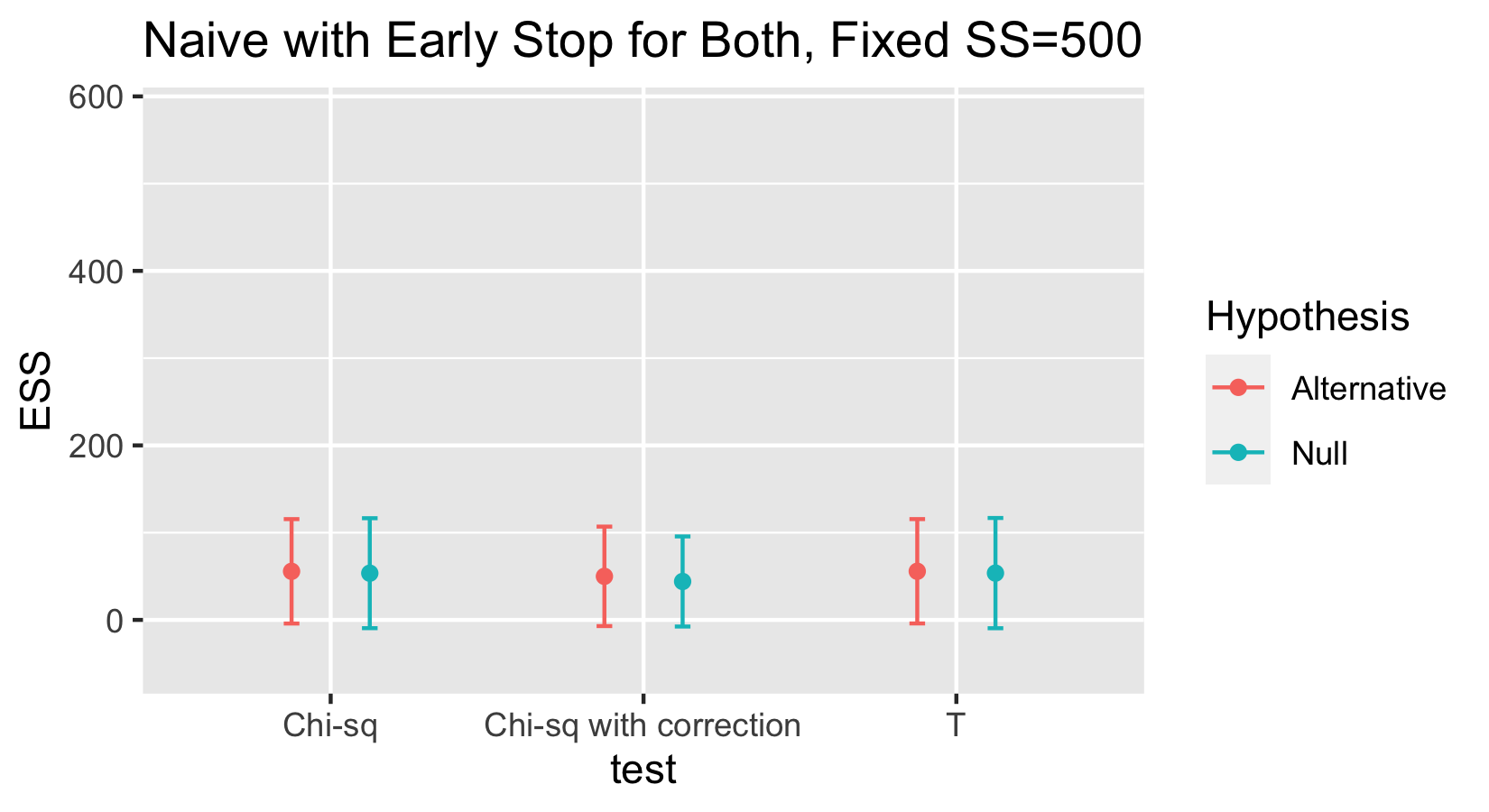

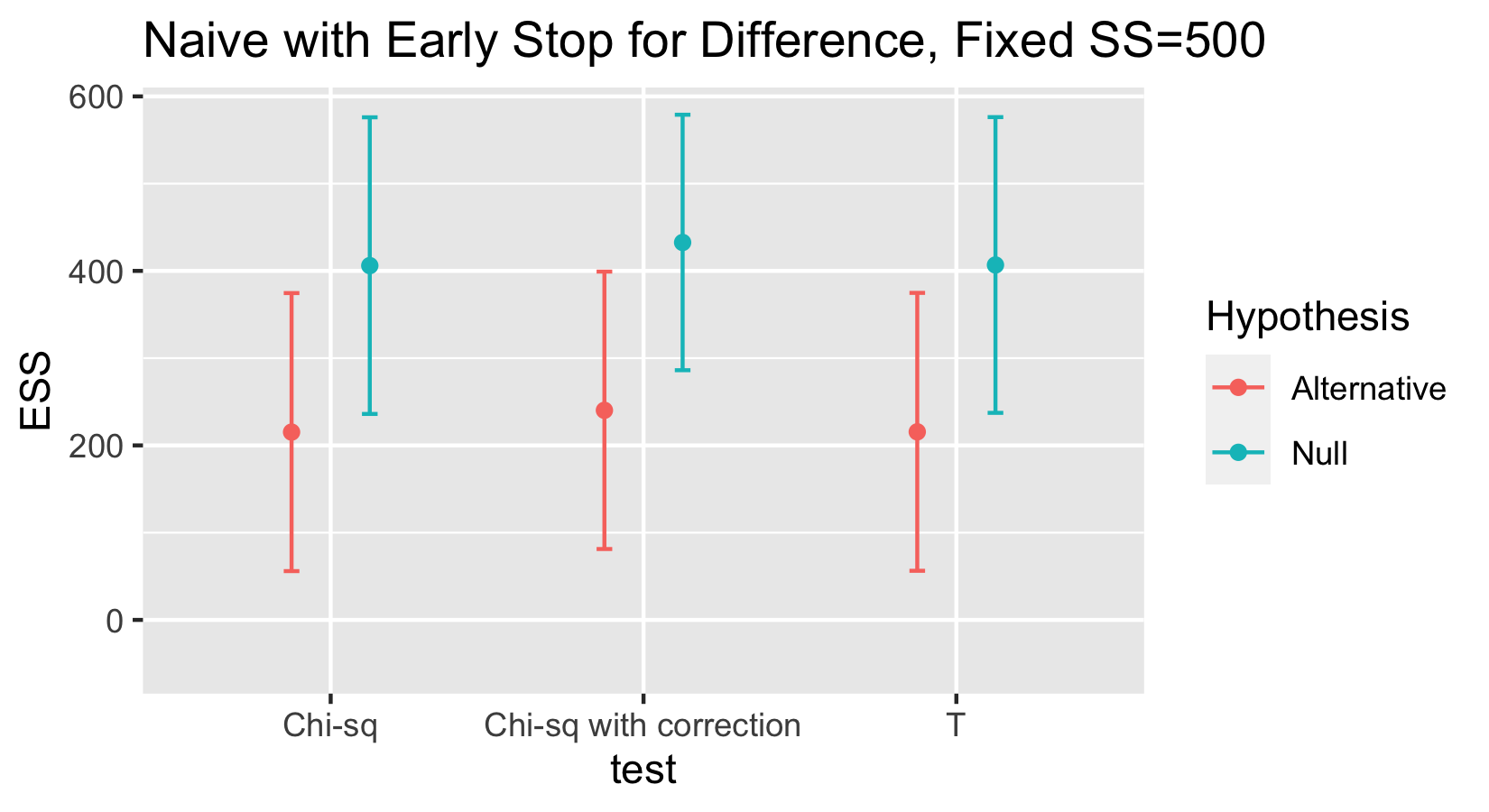


##
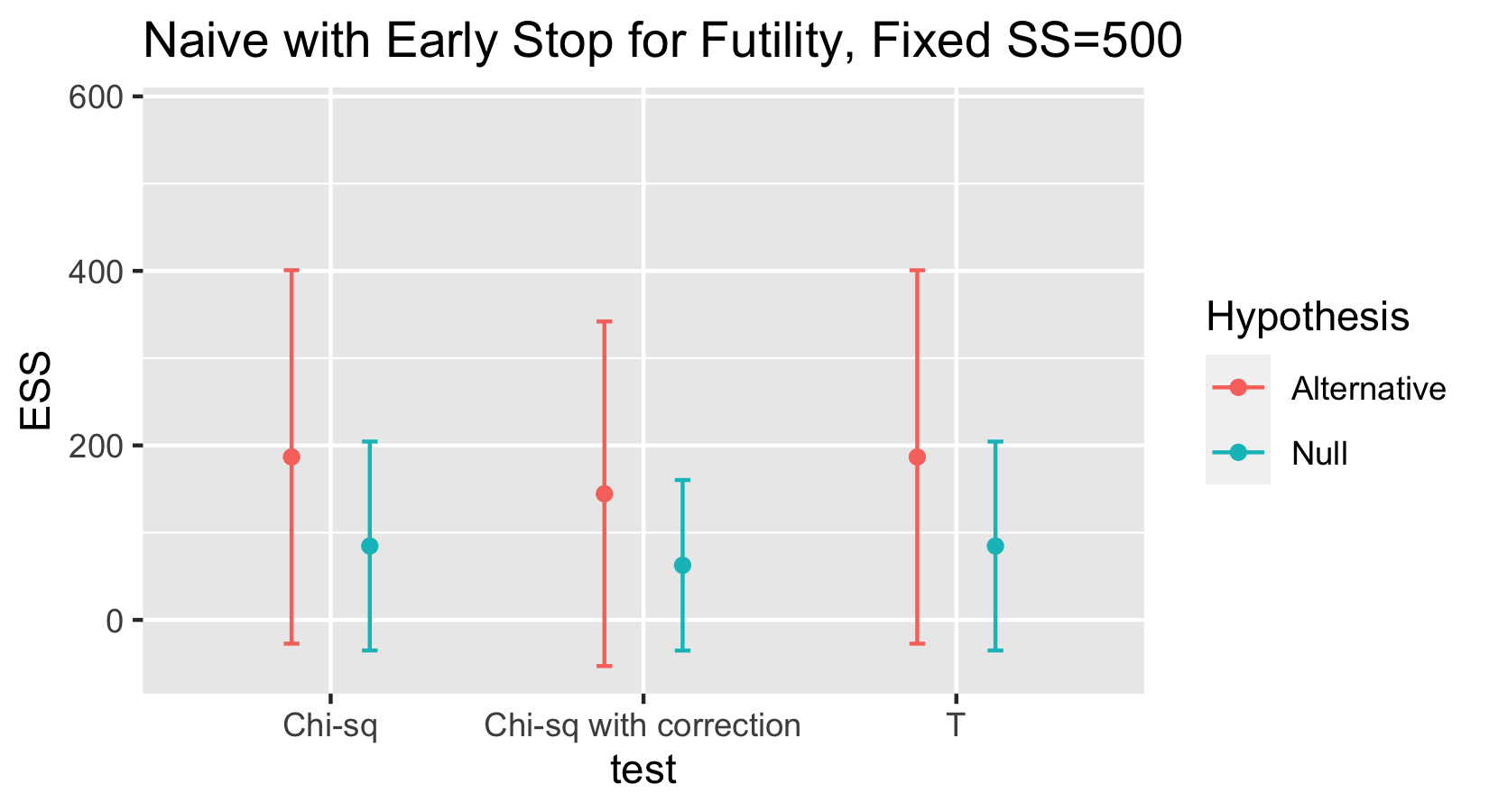

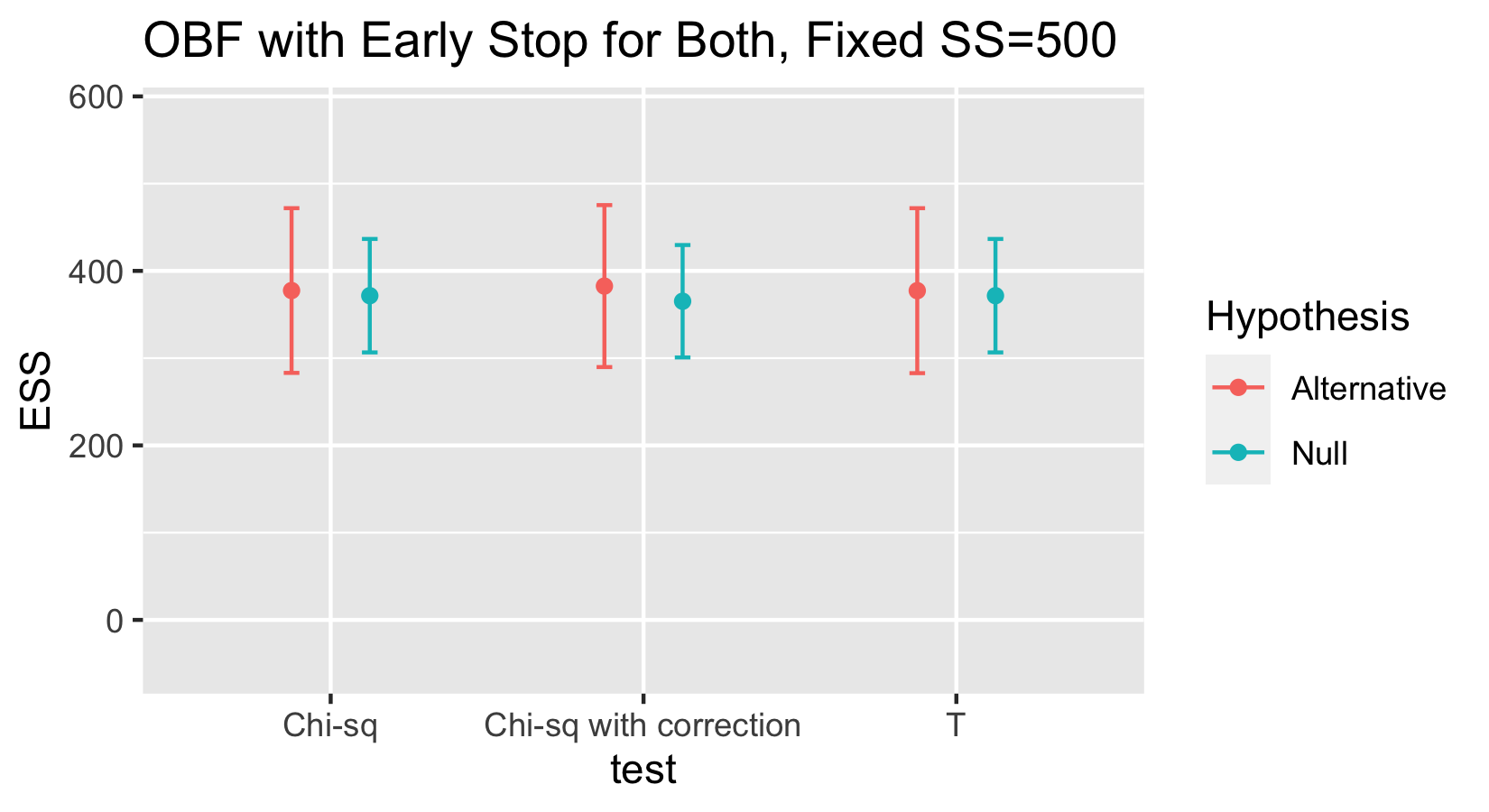

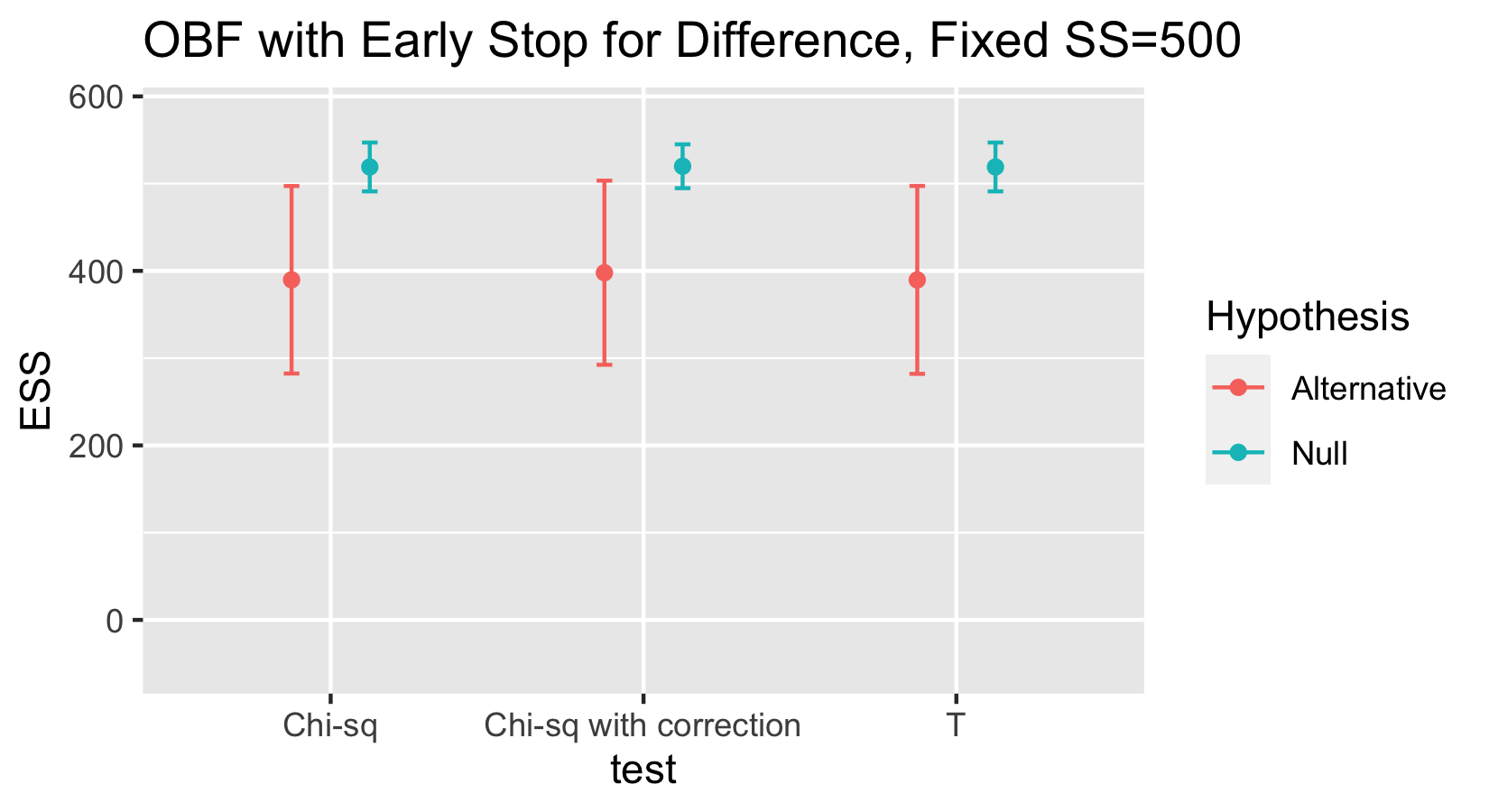


##
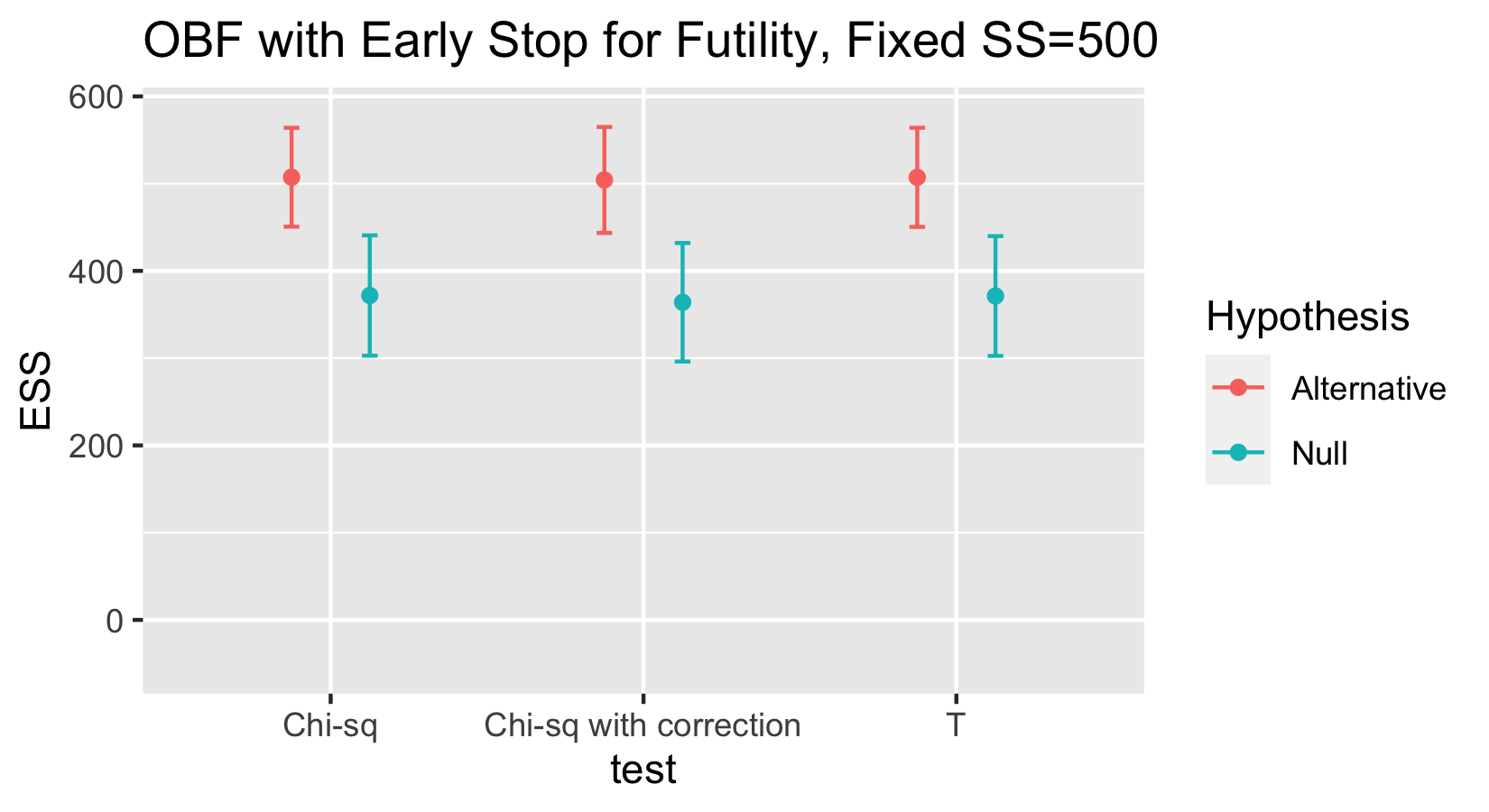


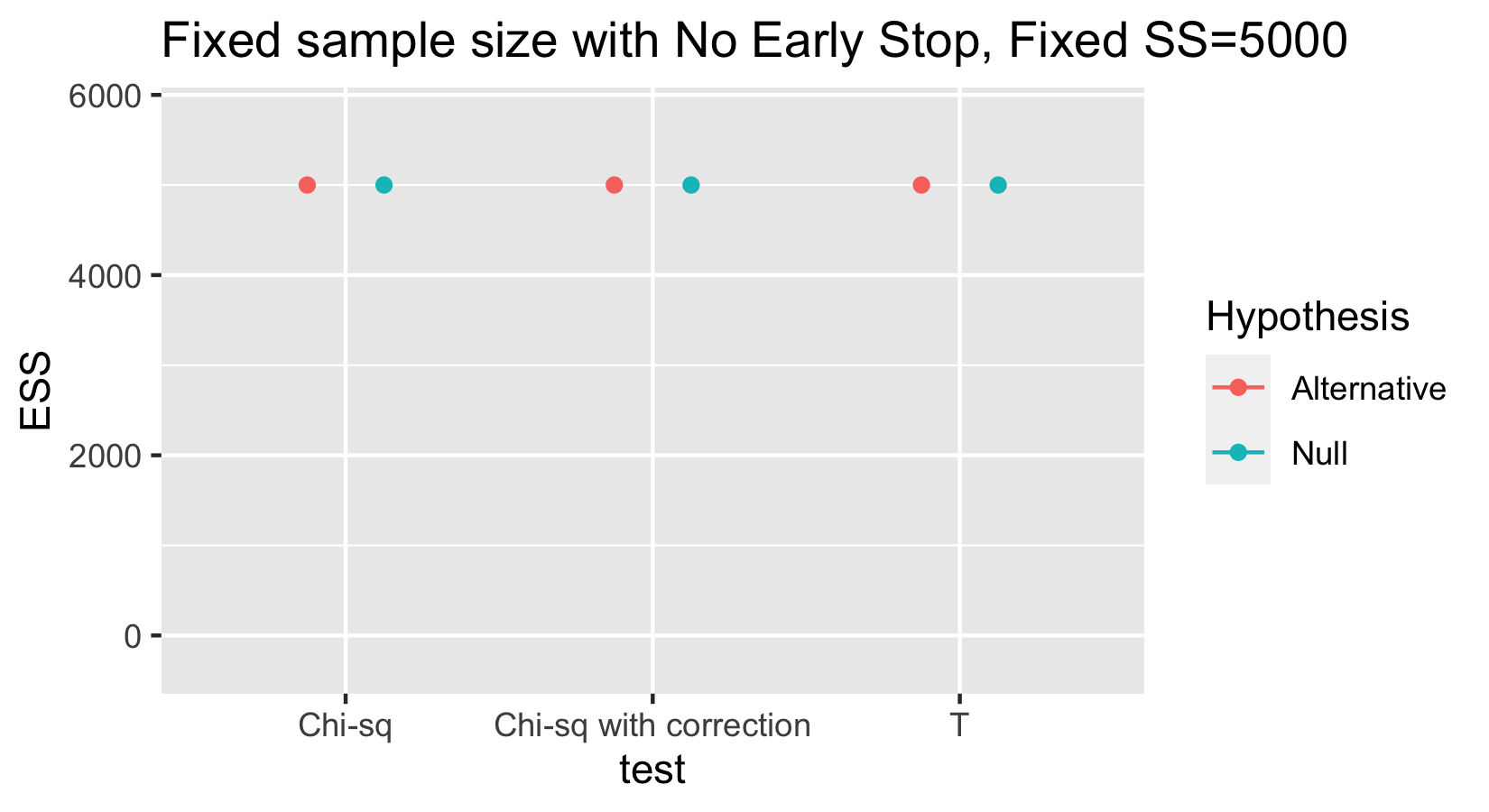

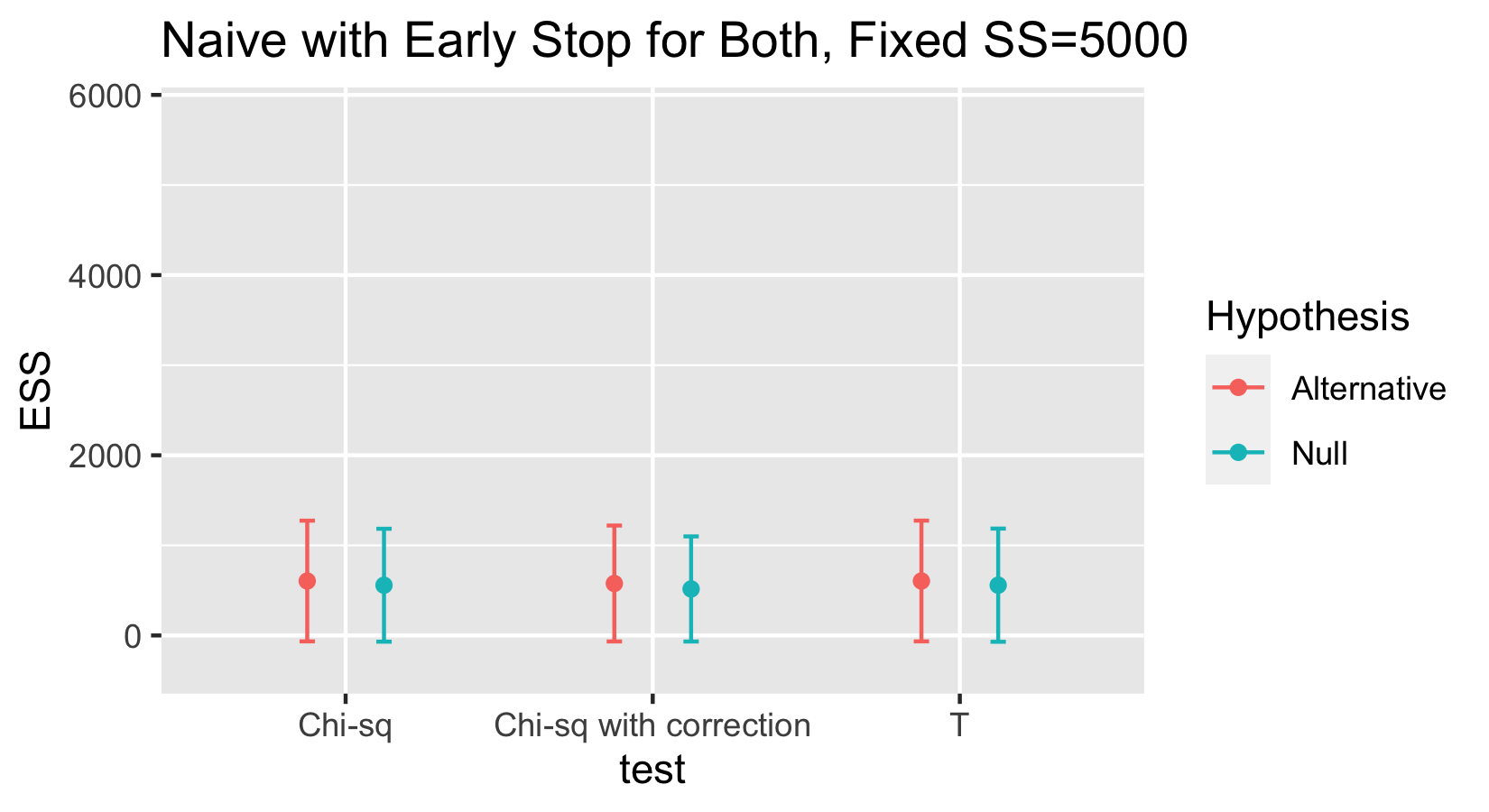


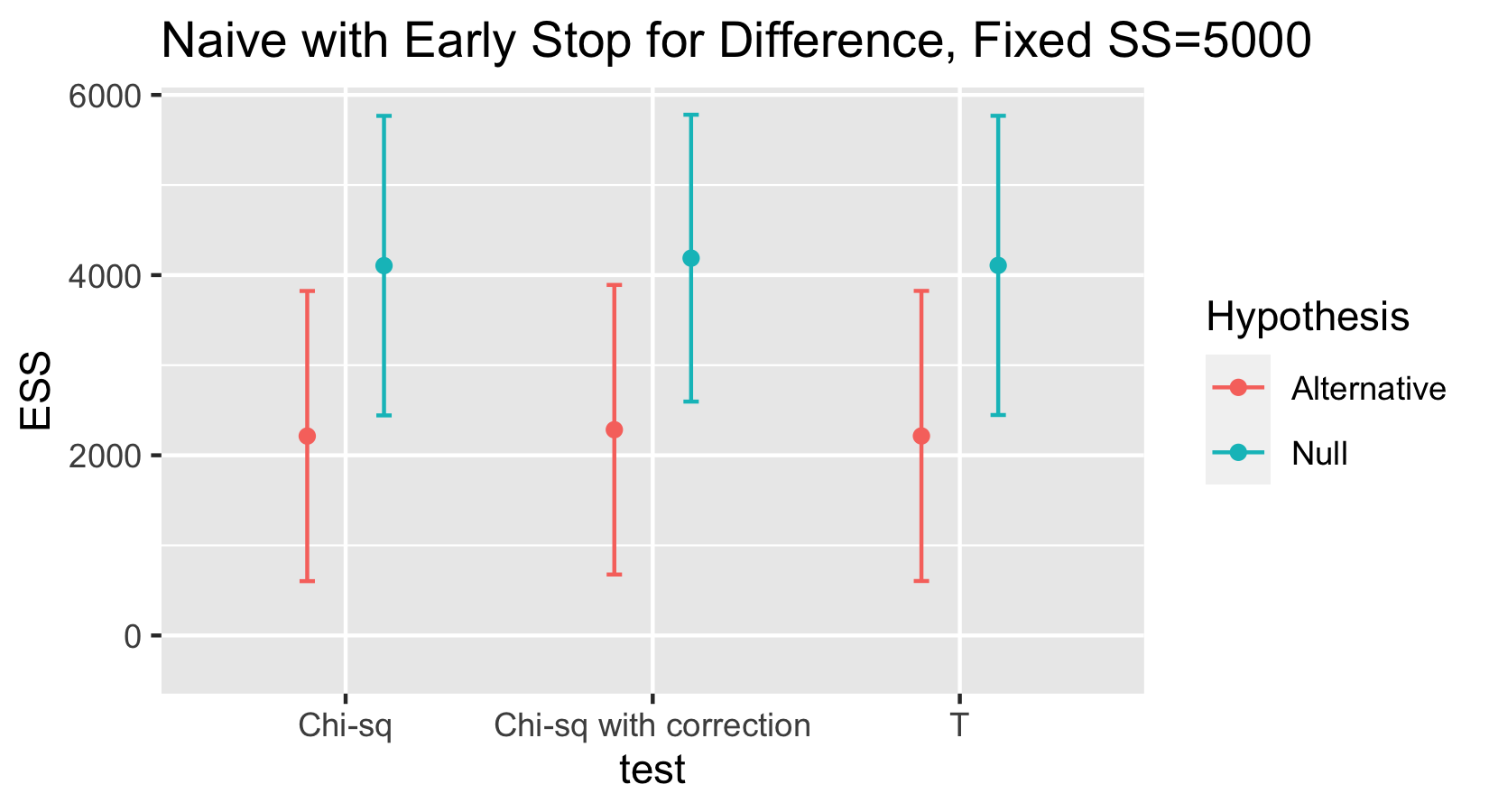

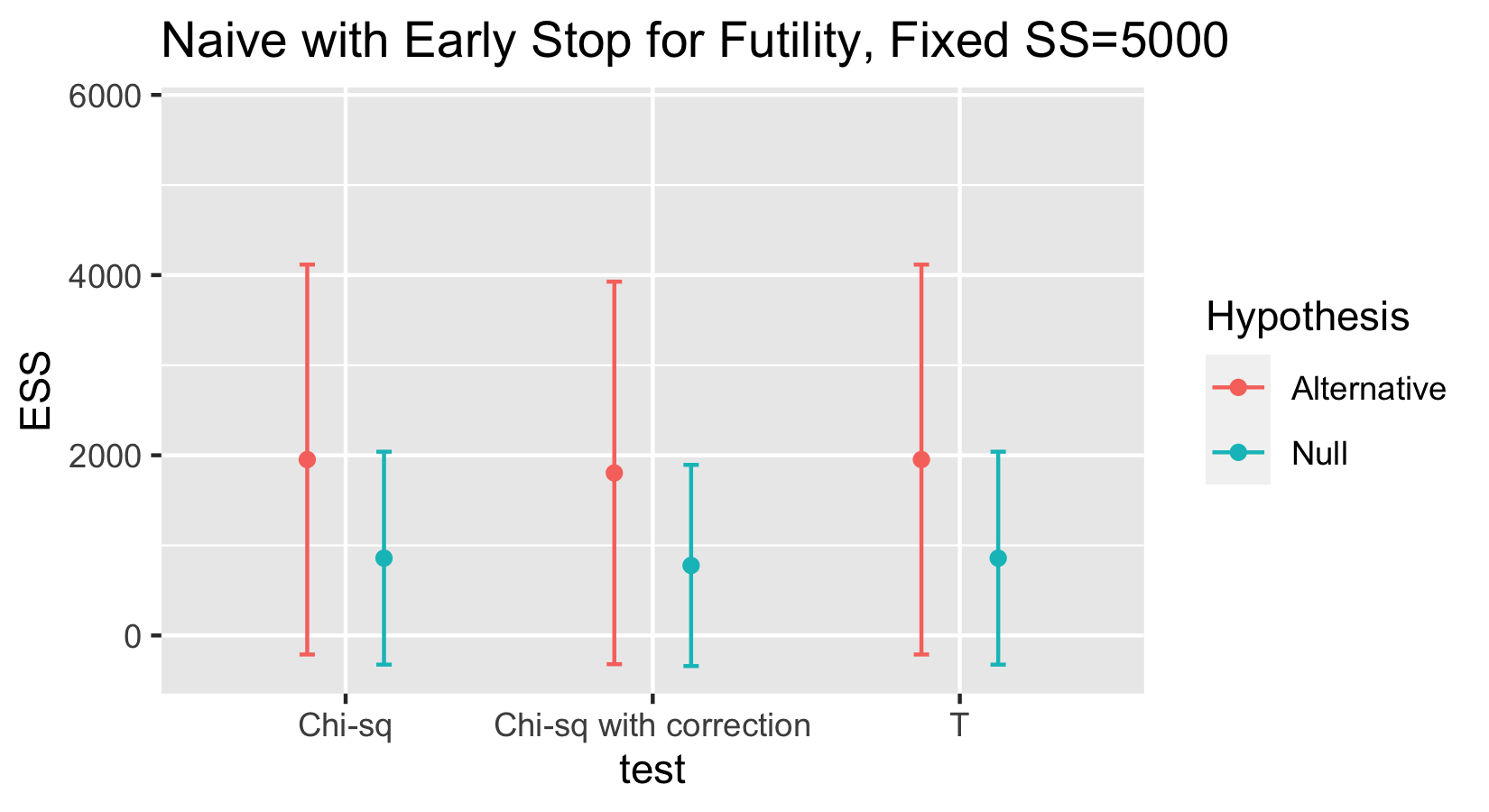

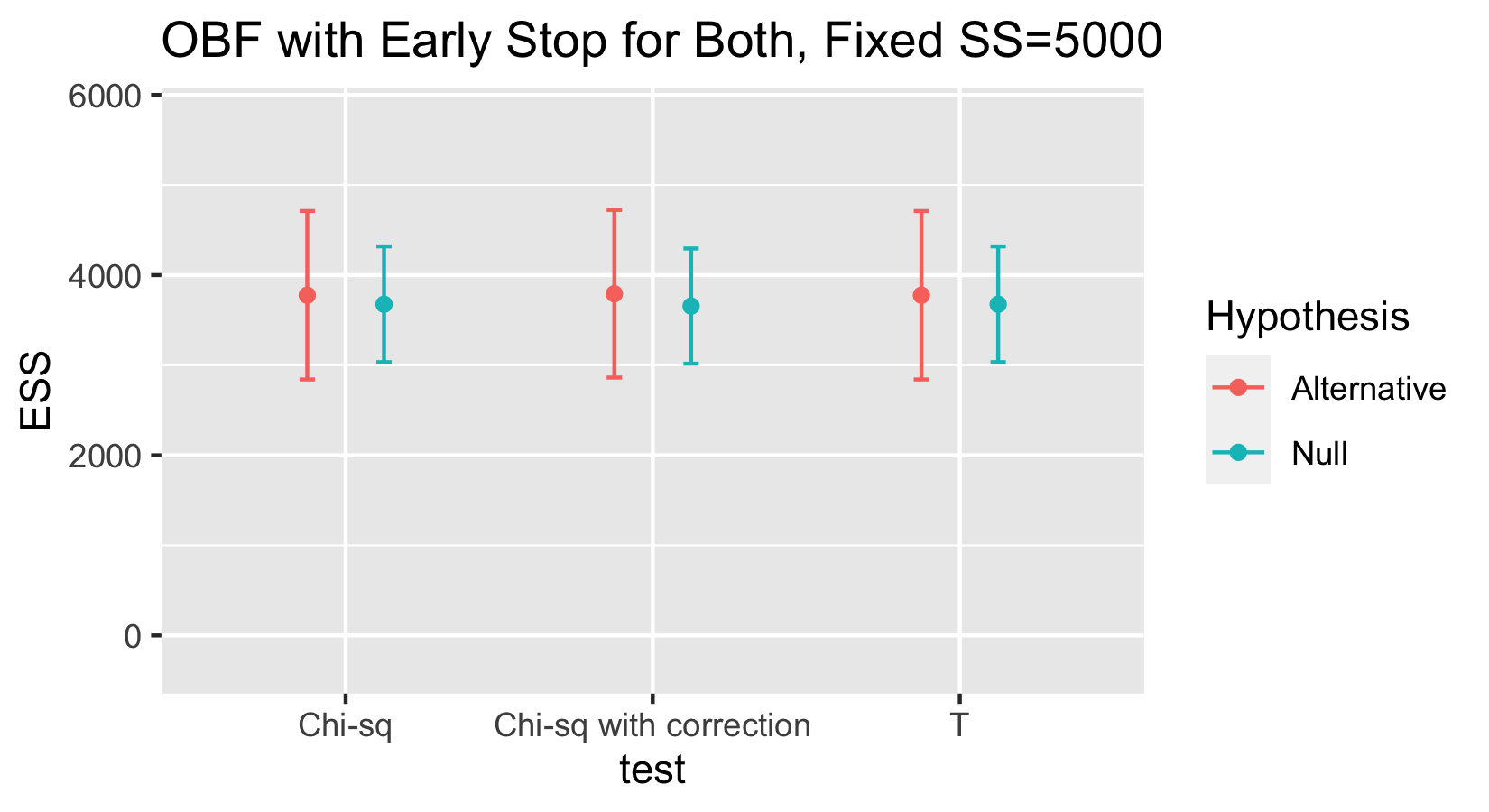


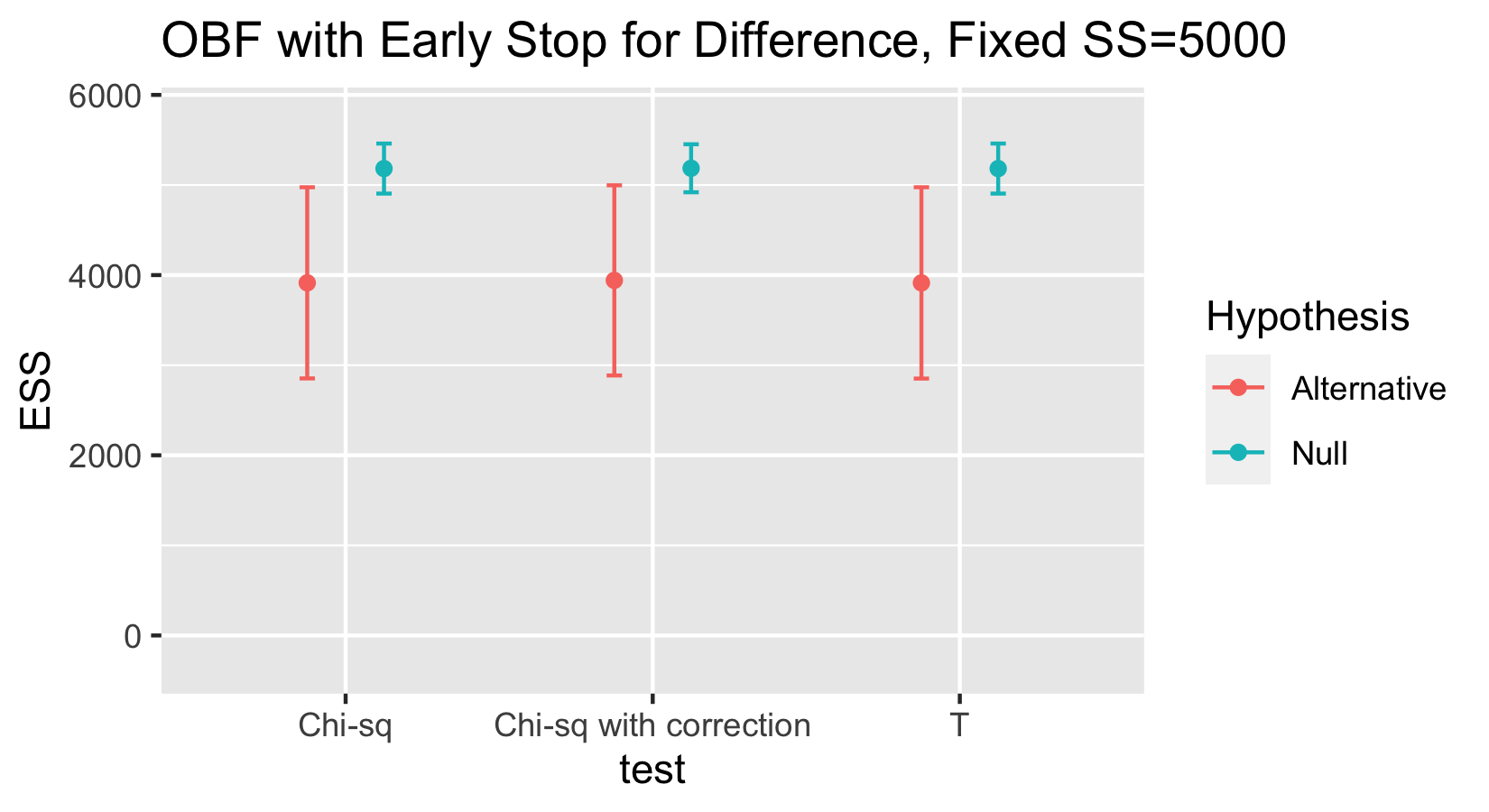

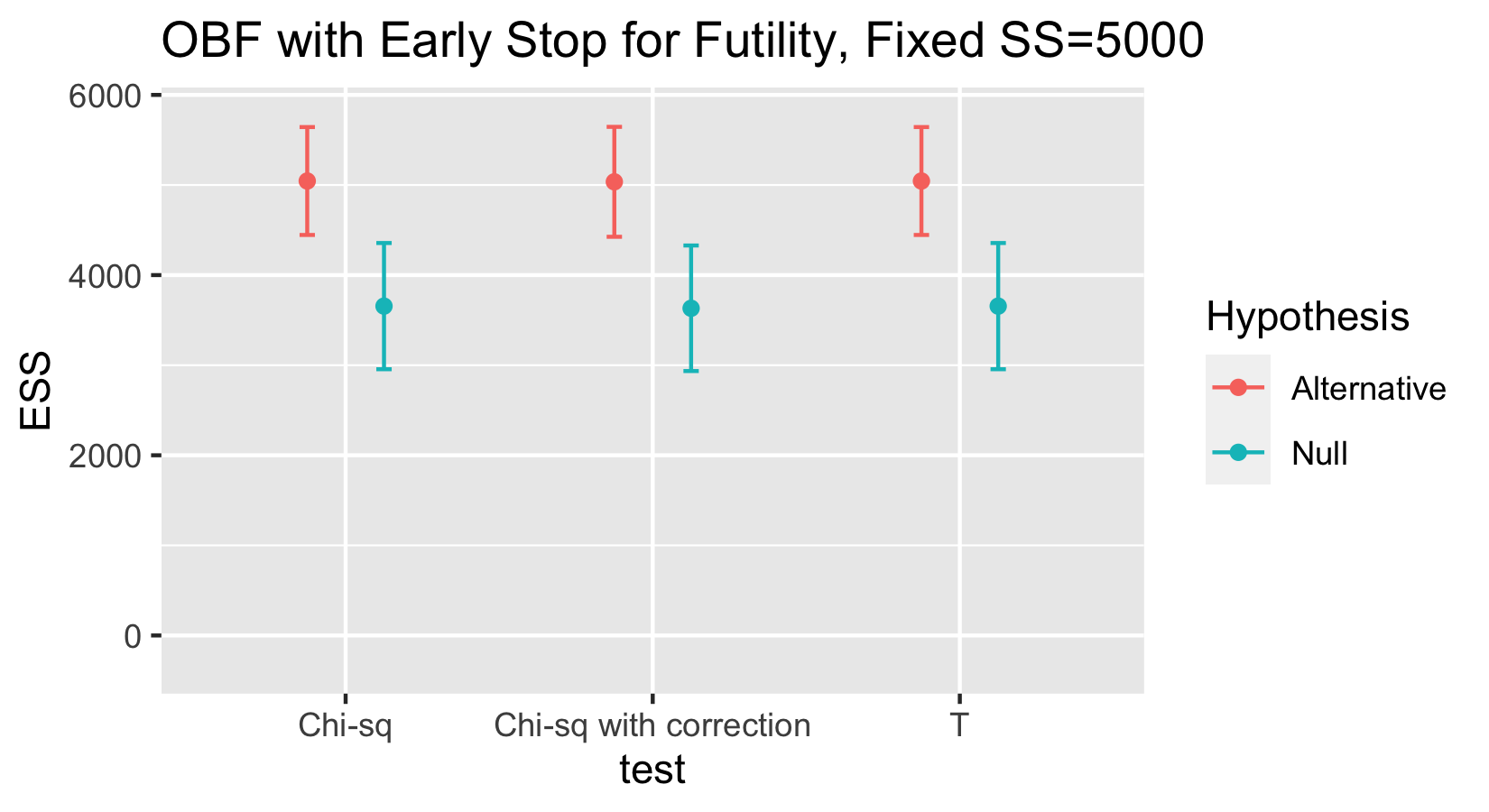


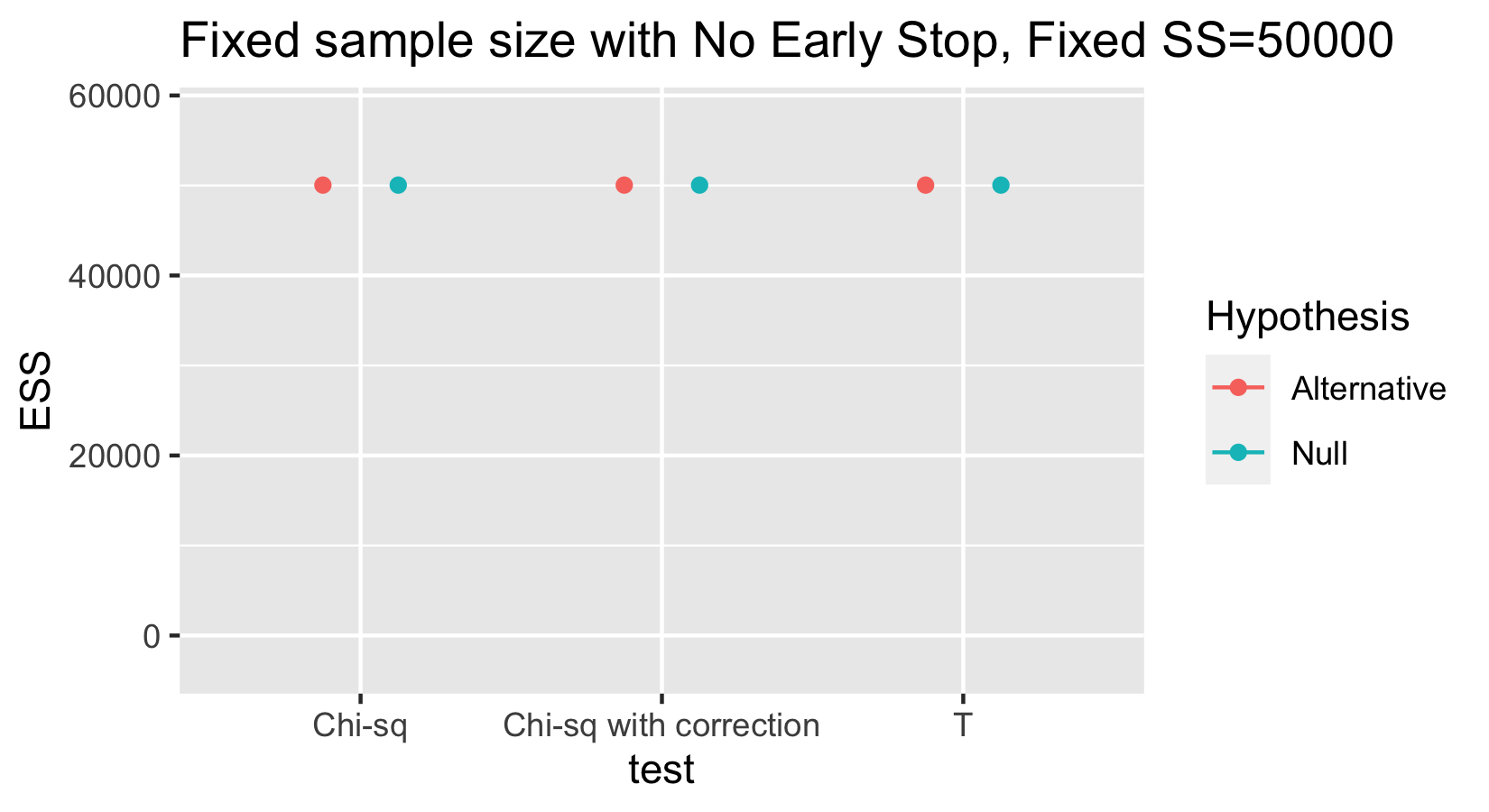


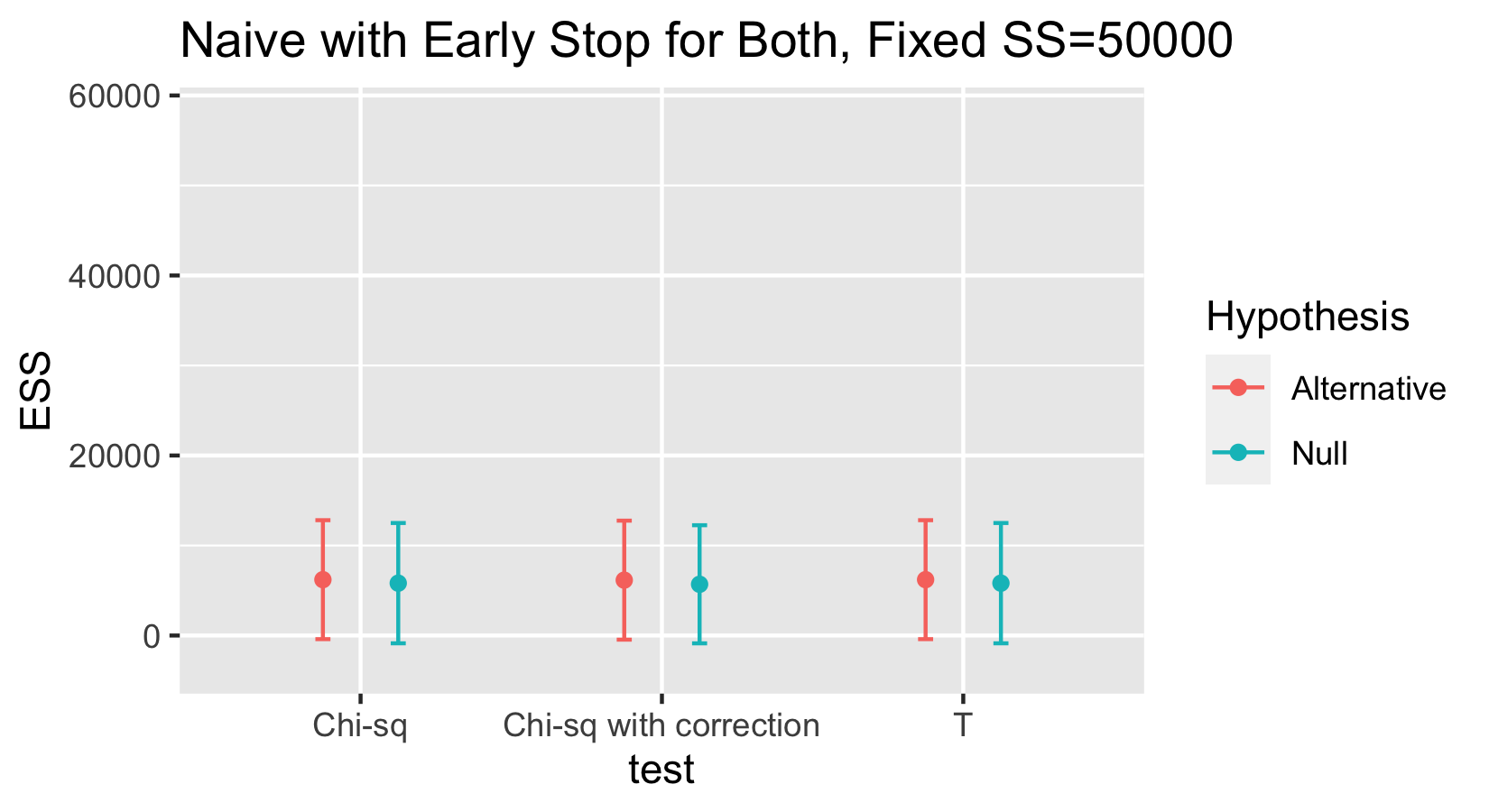

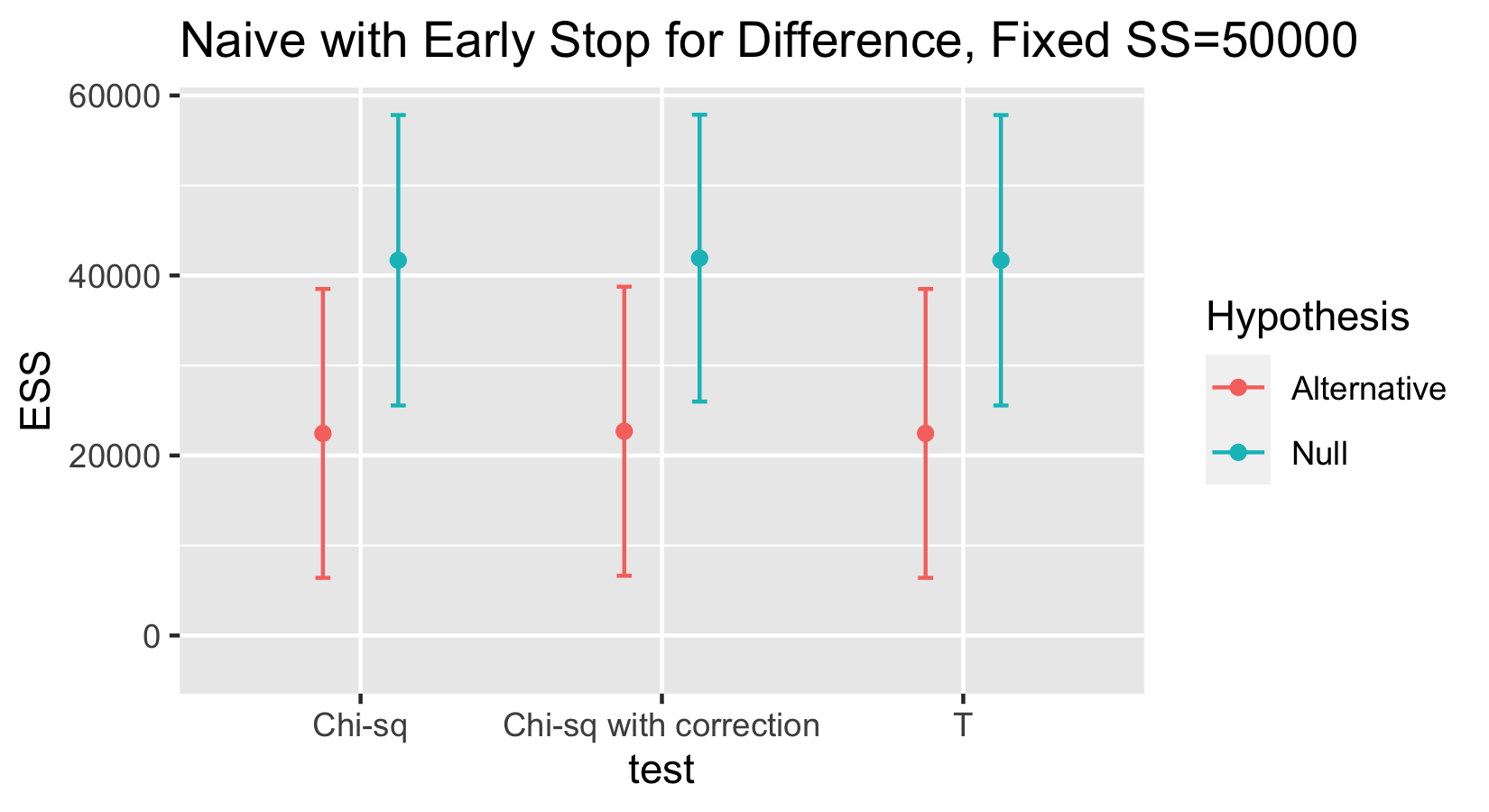

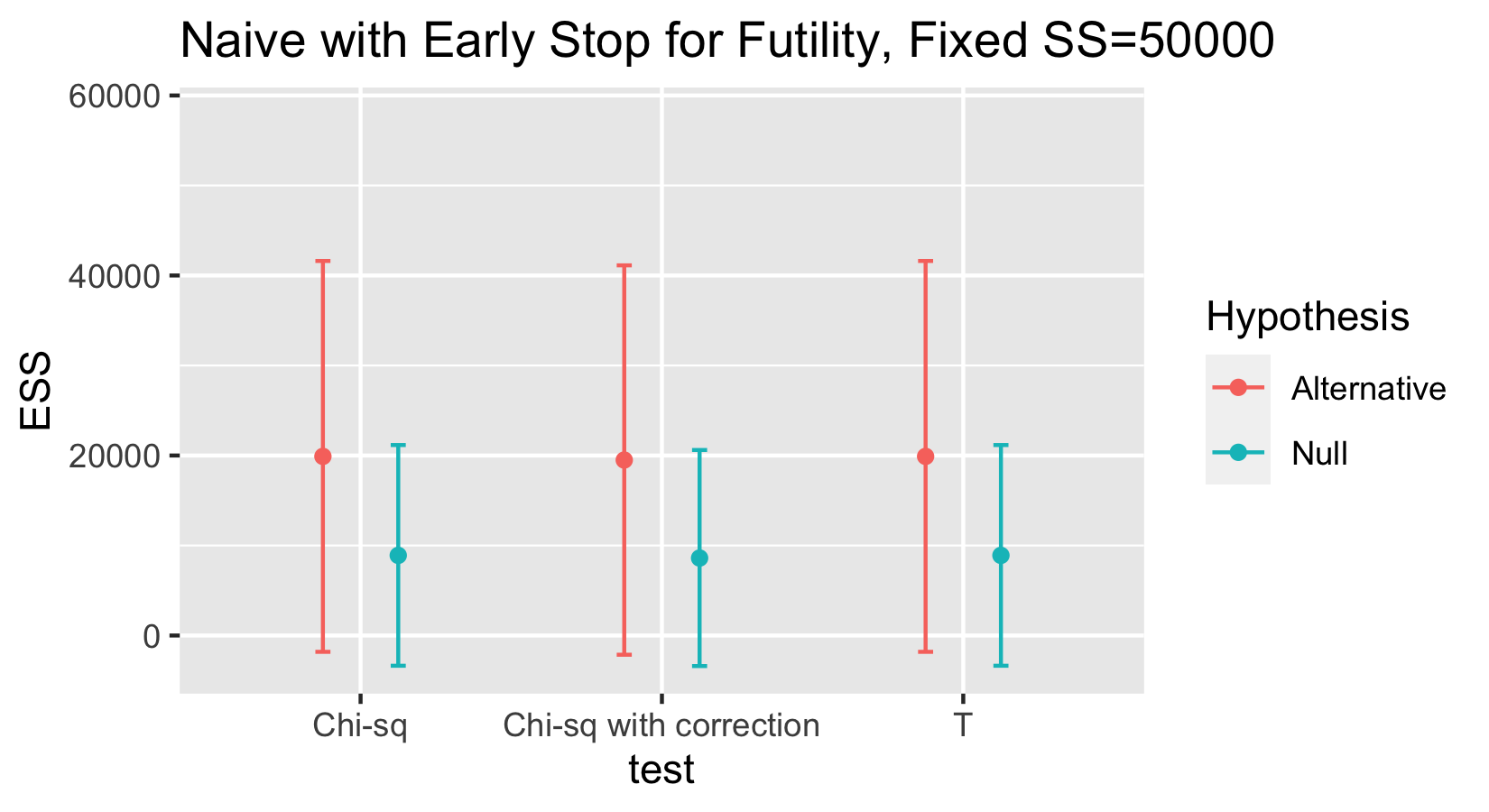


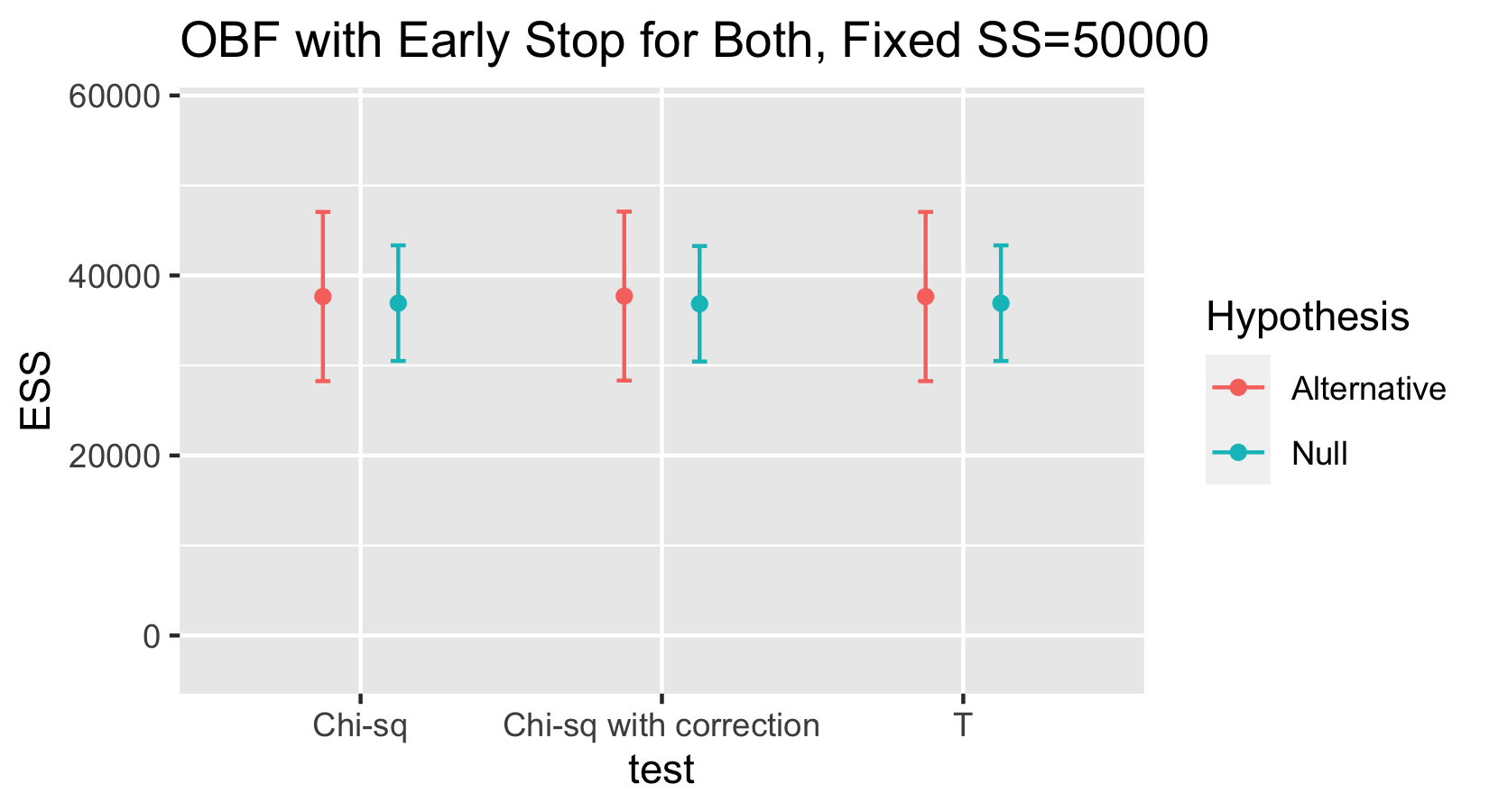

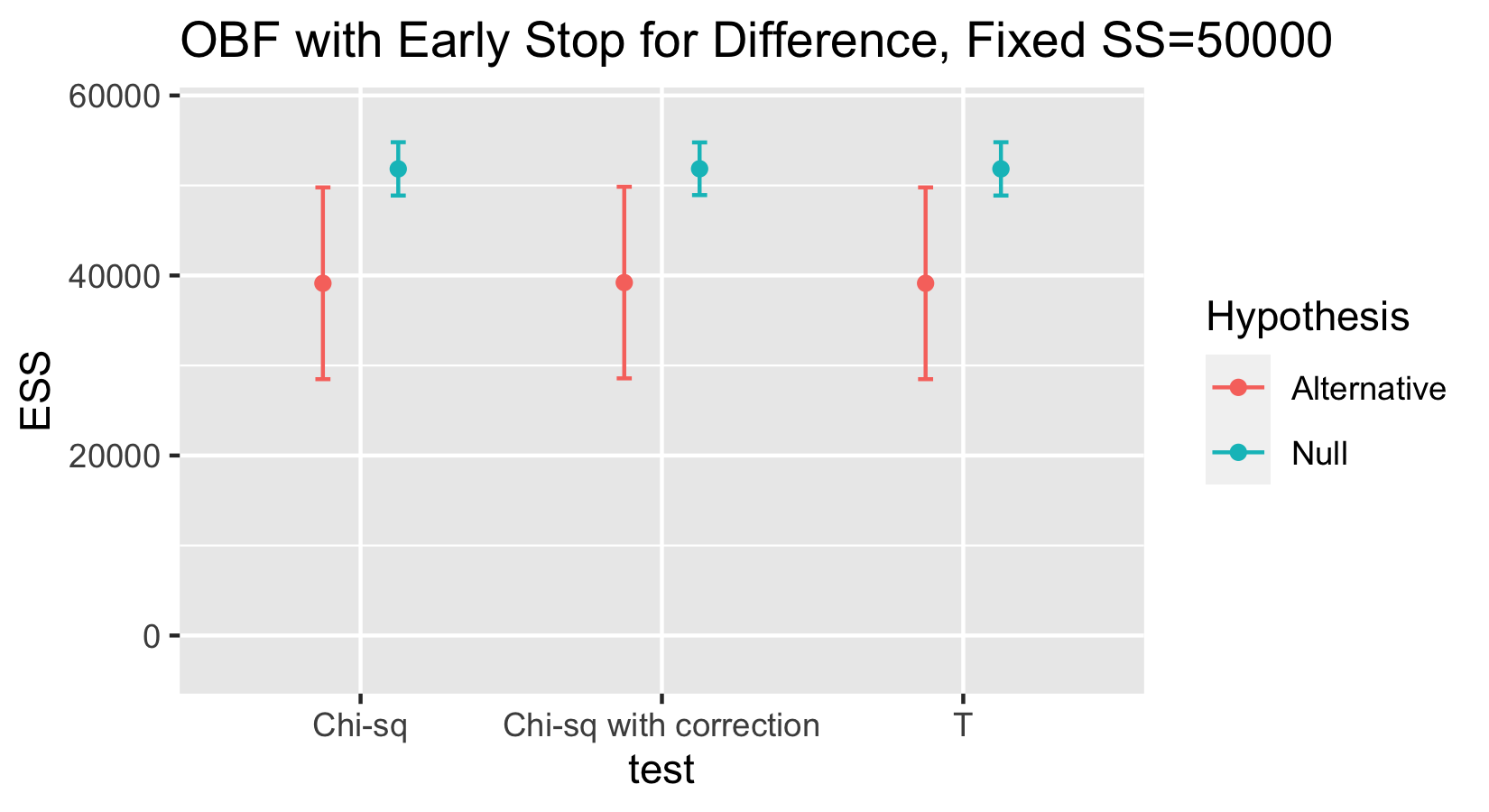

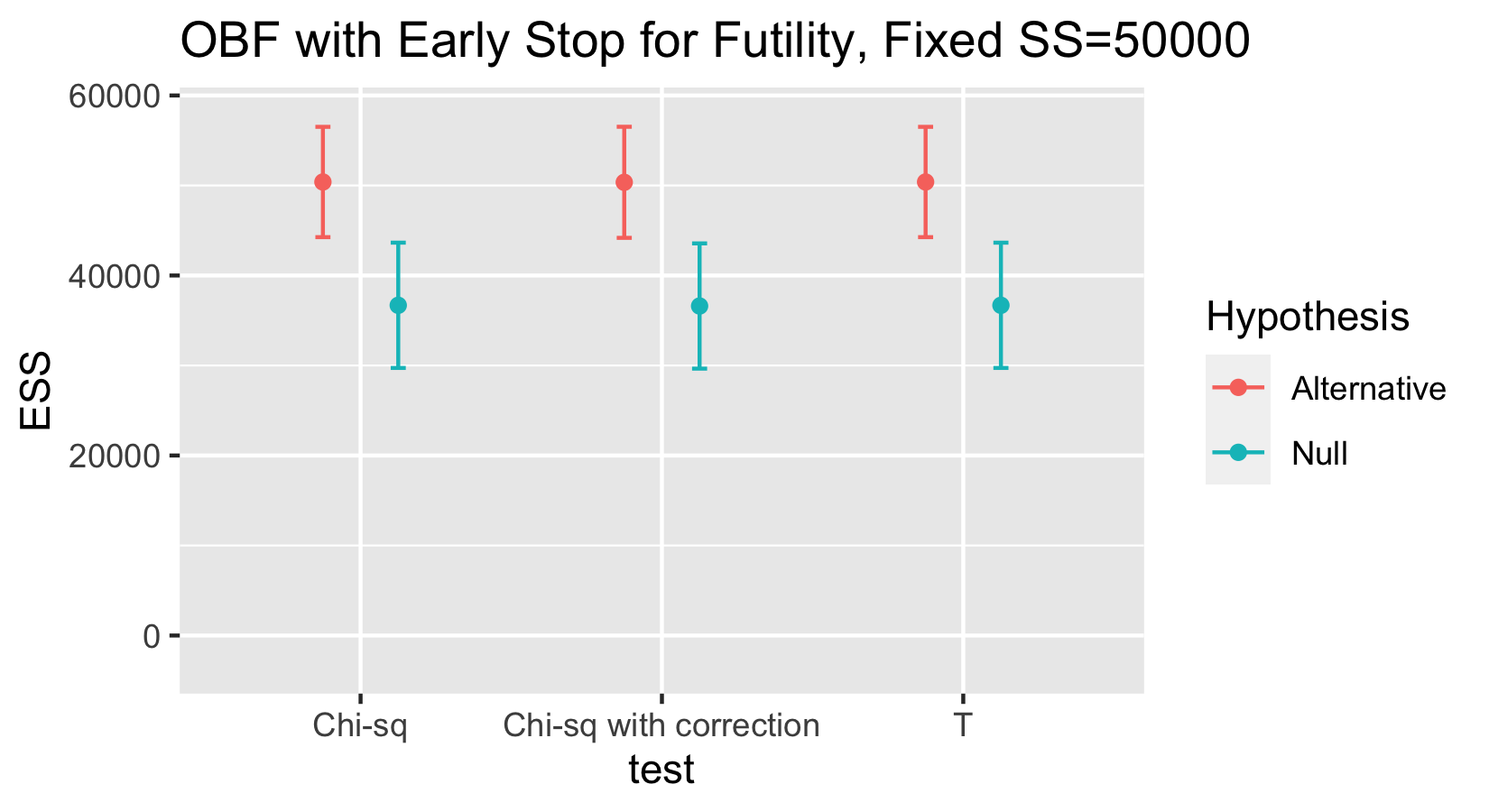


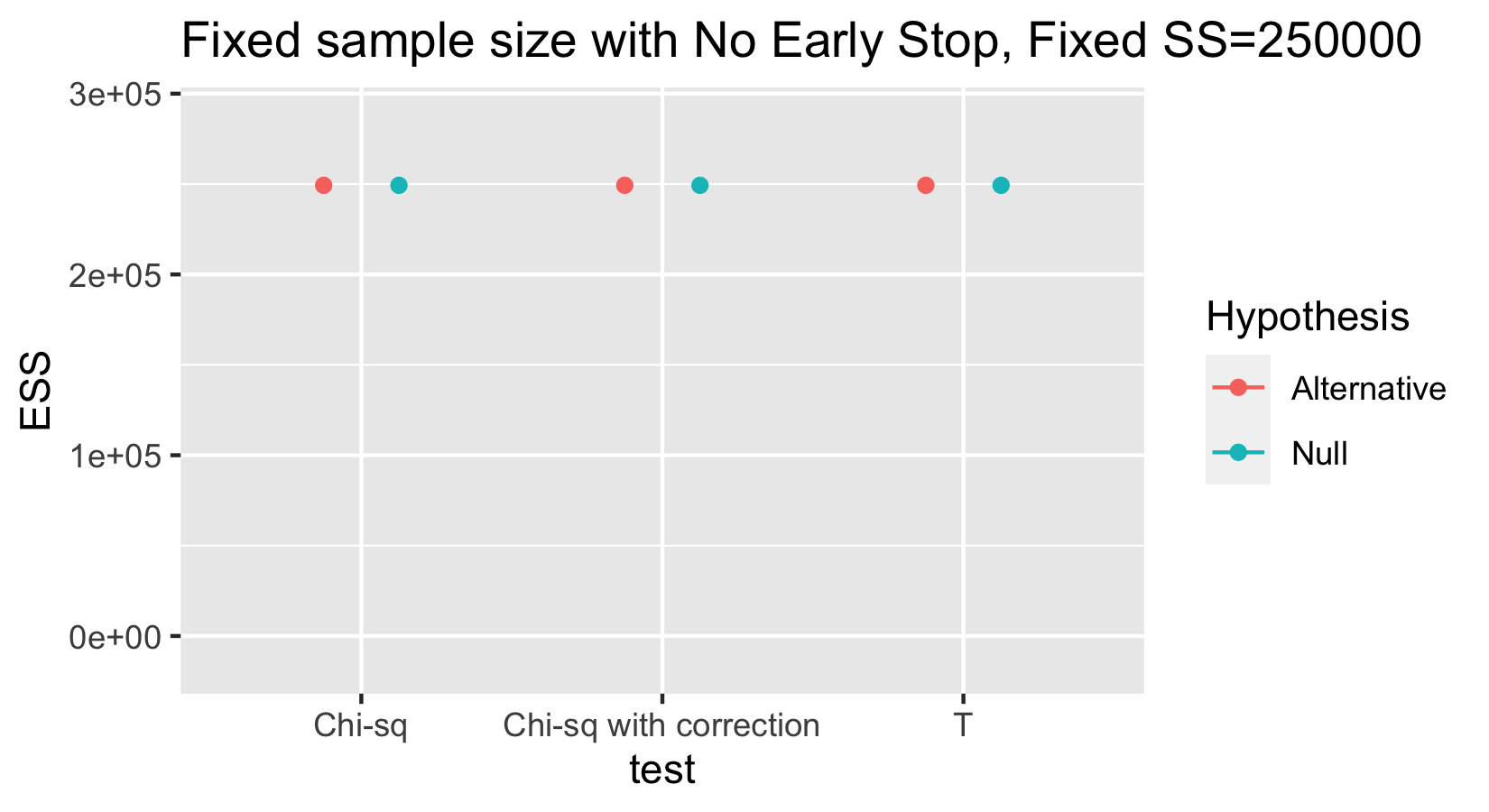

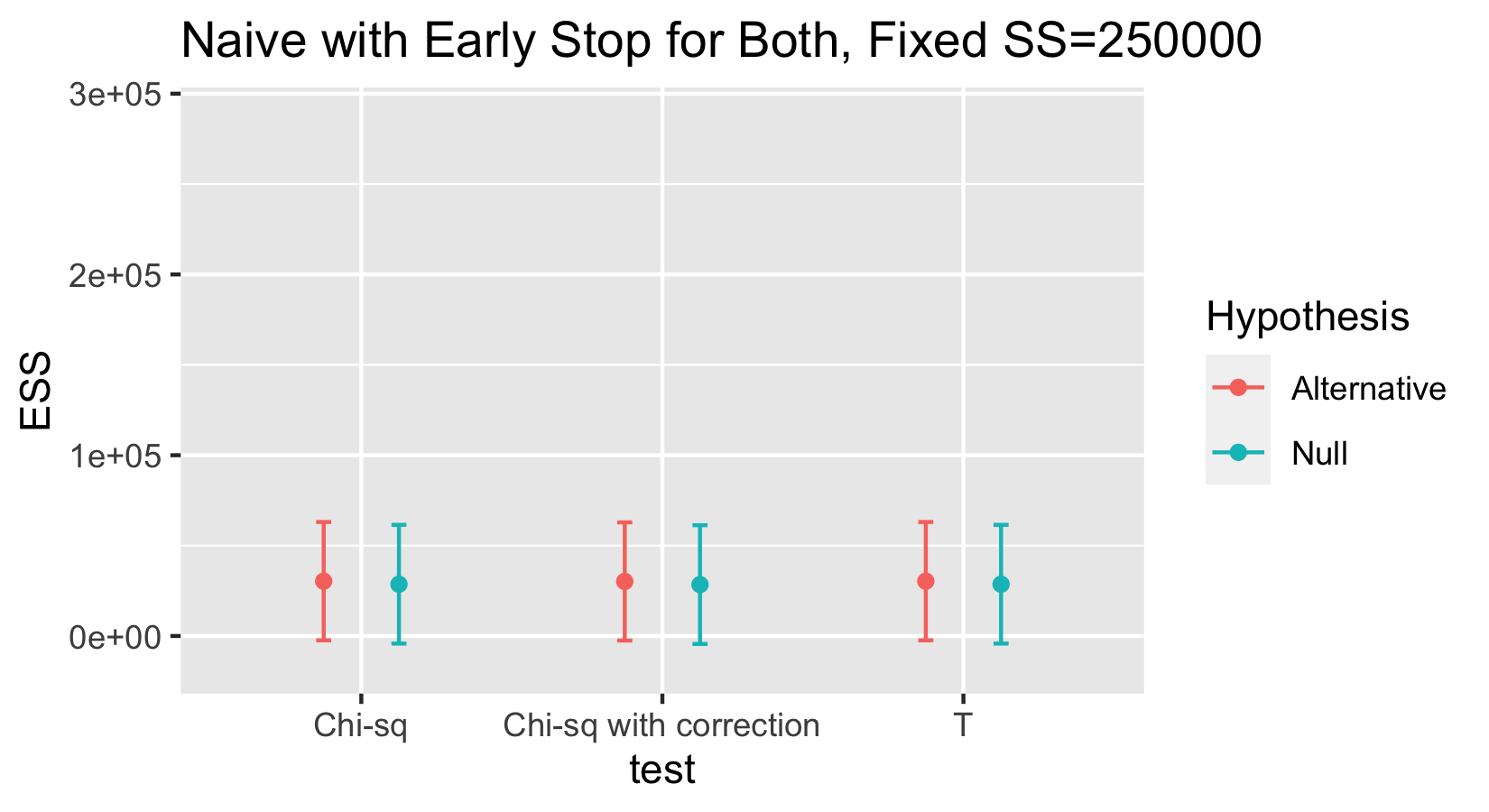

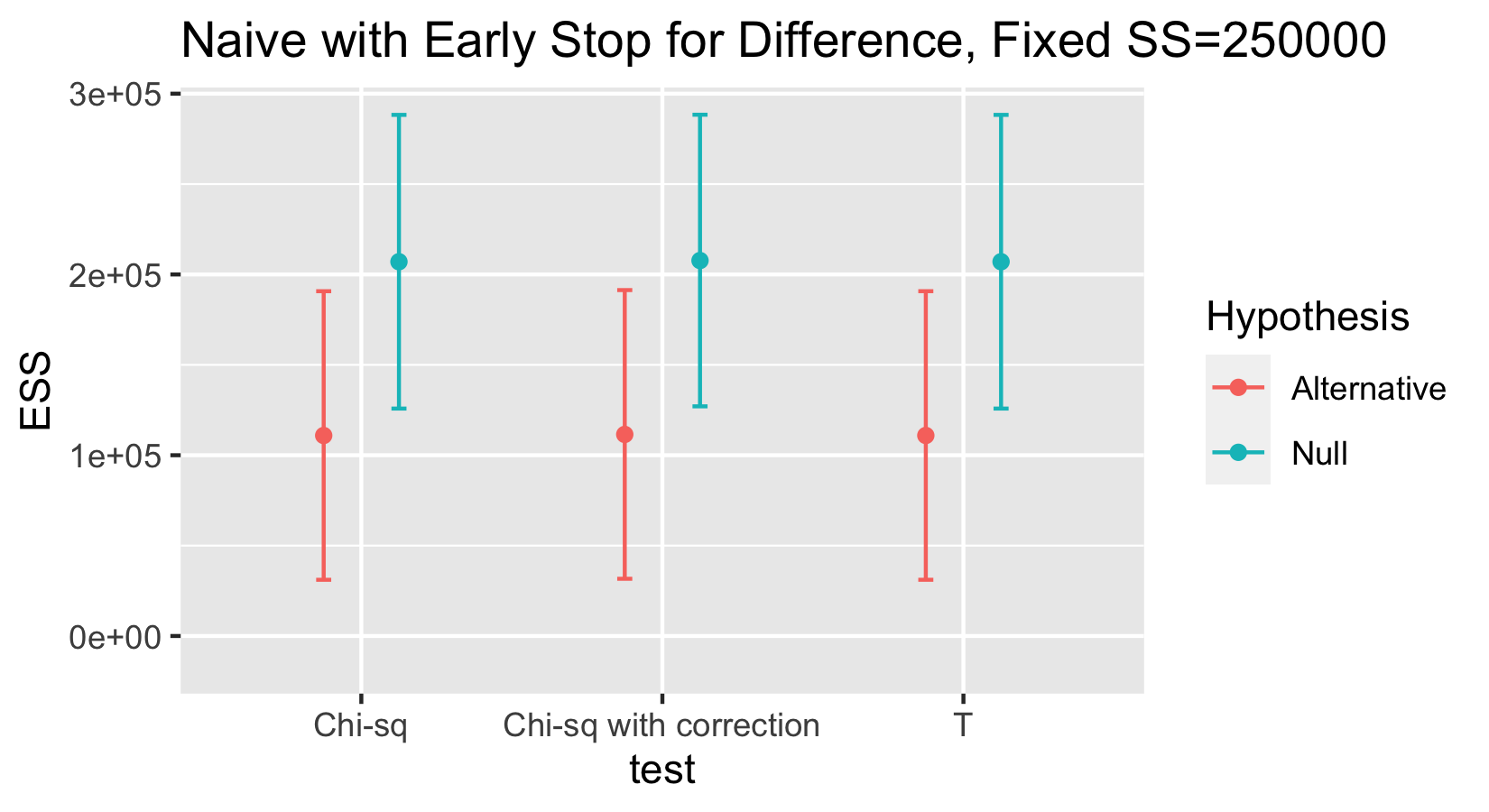


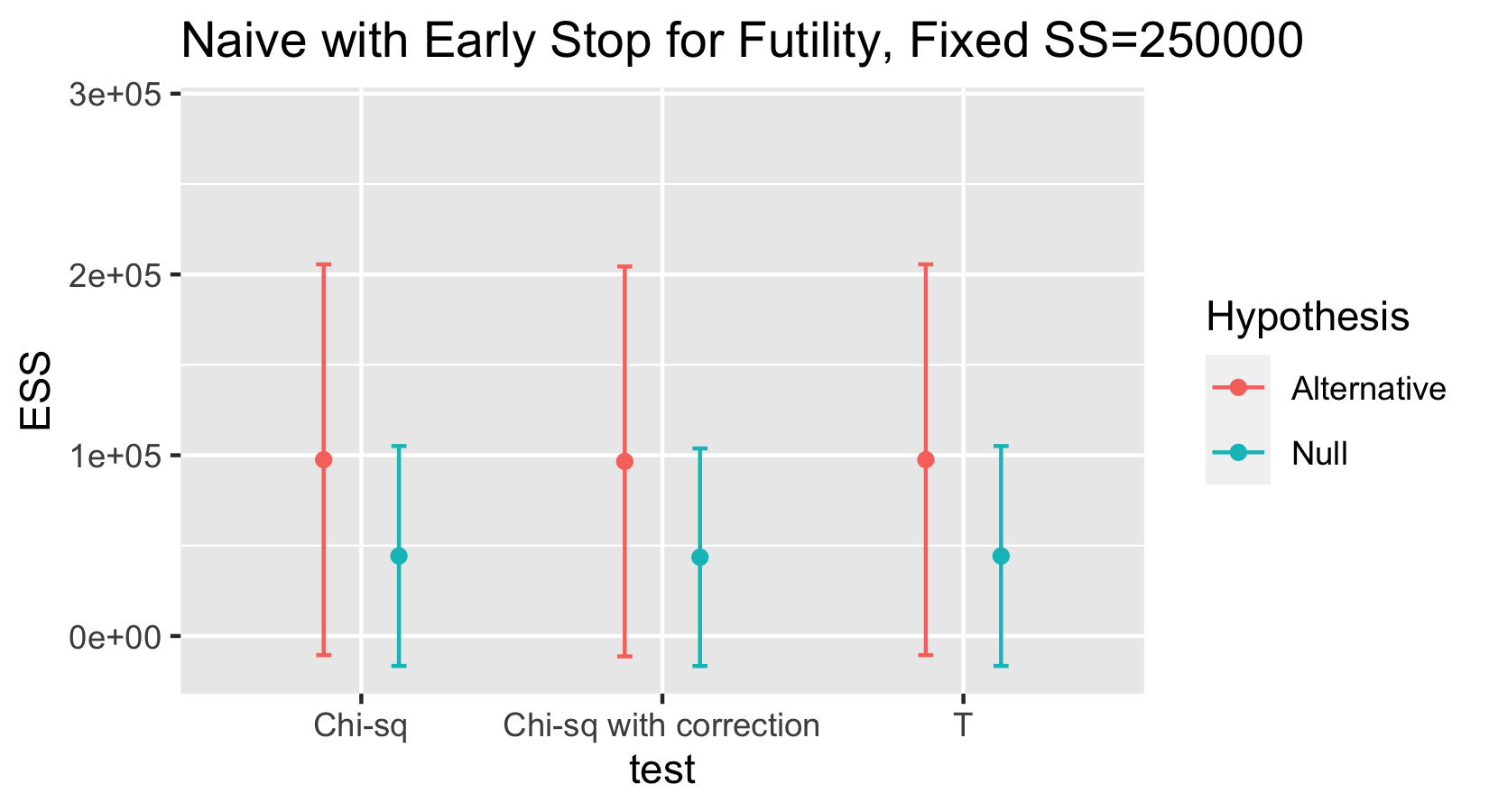

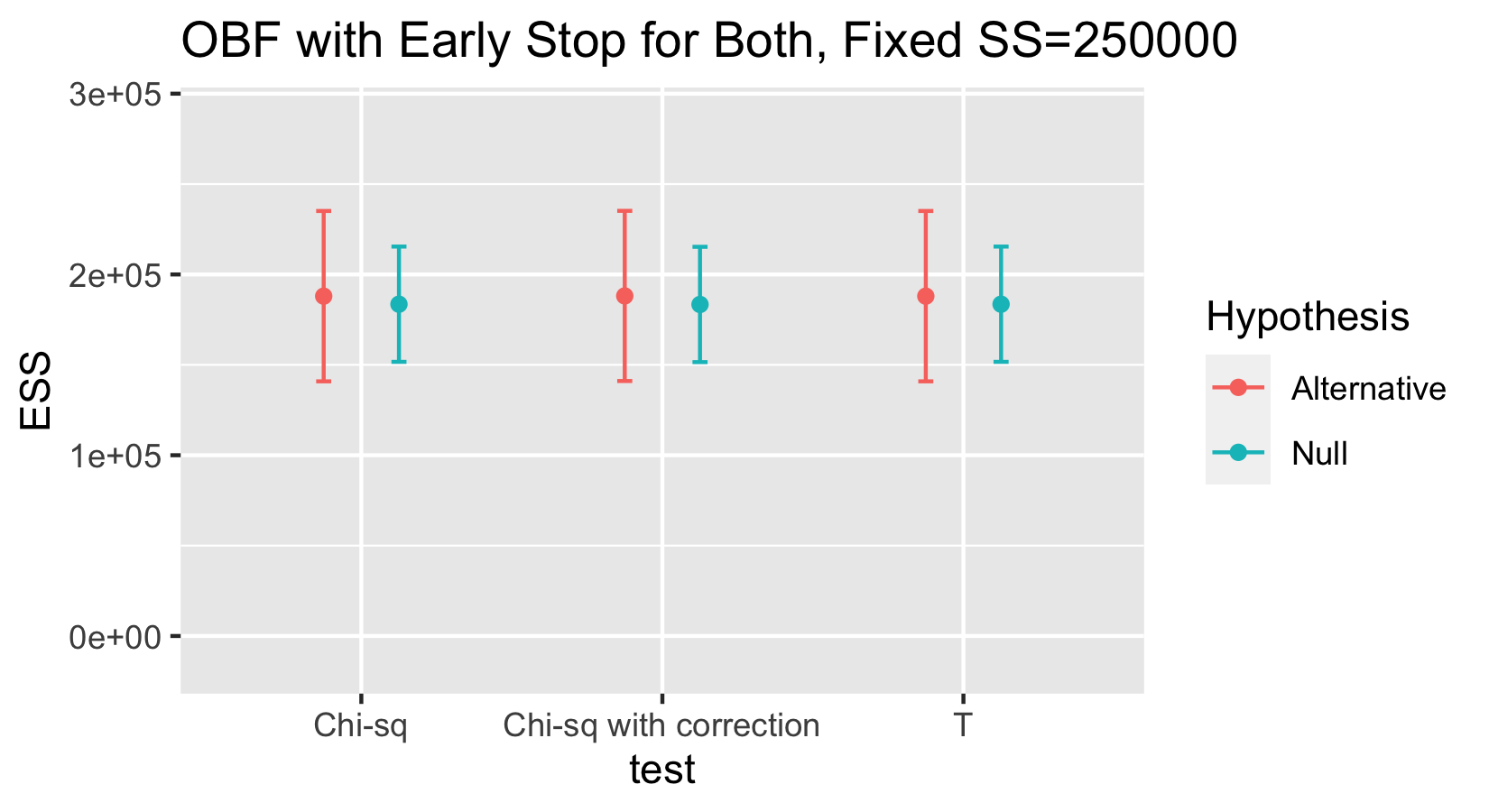

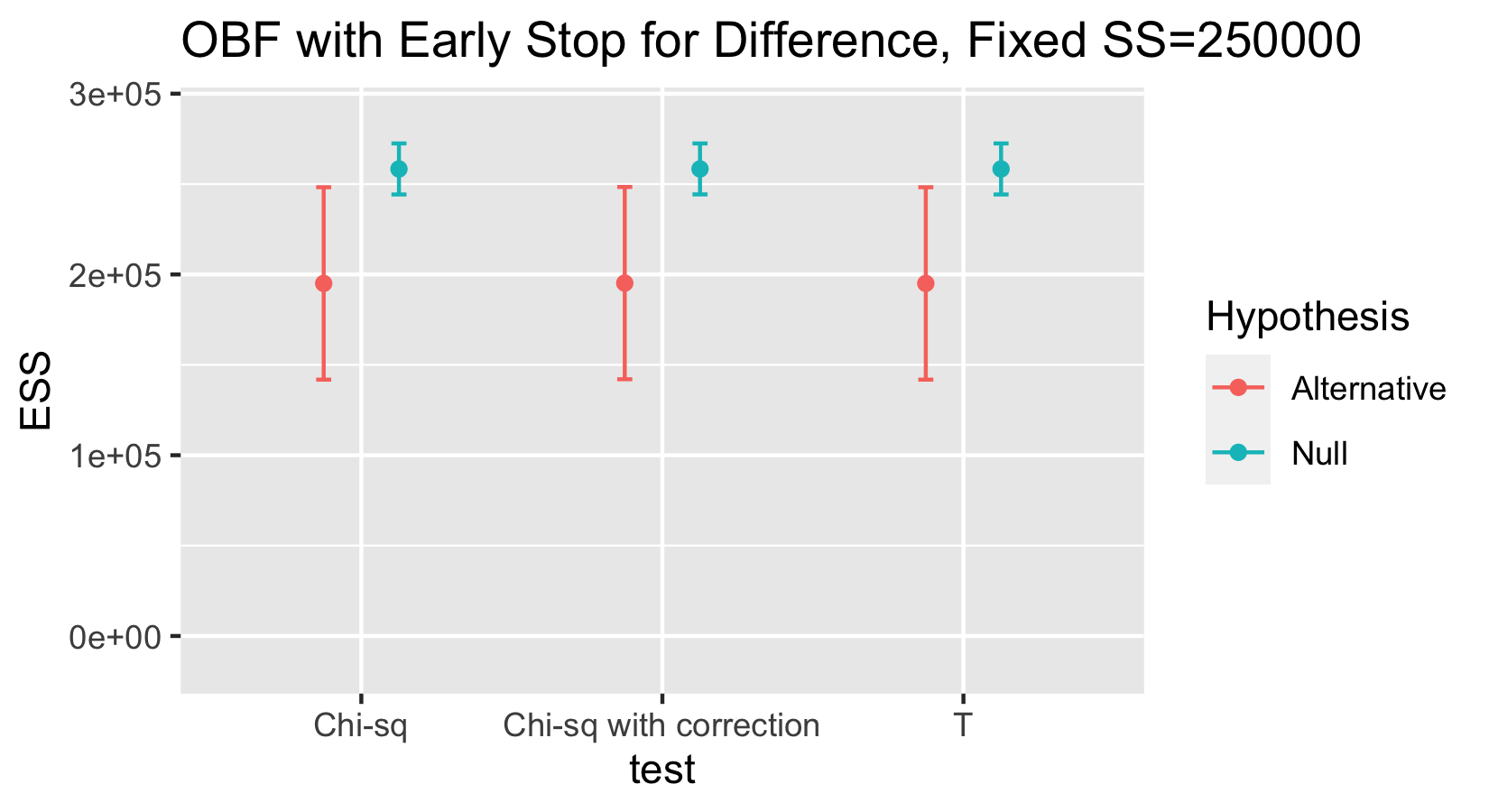


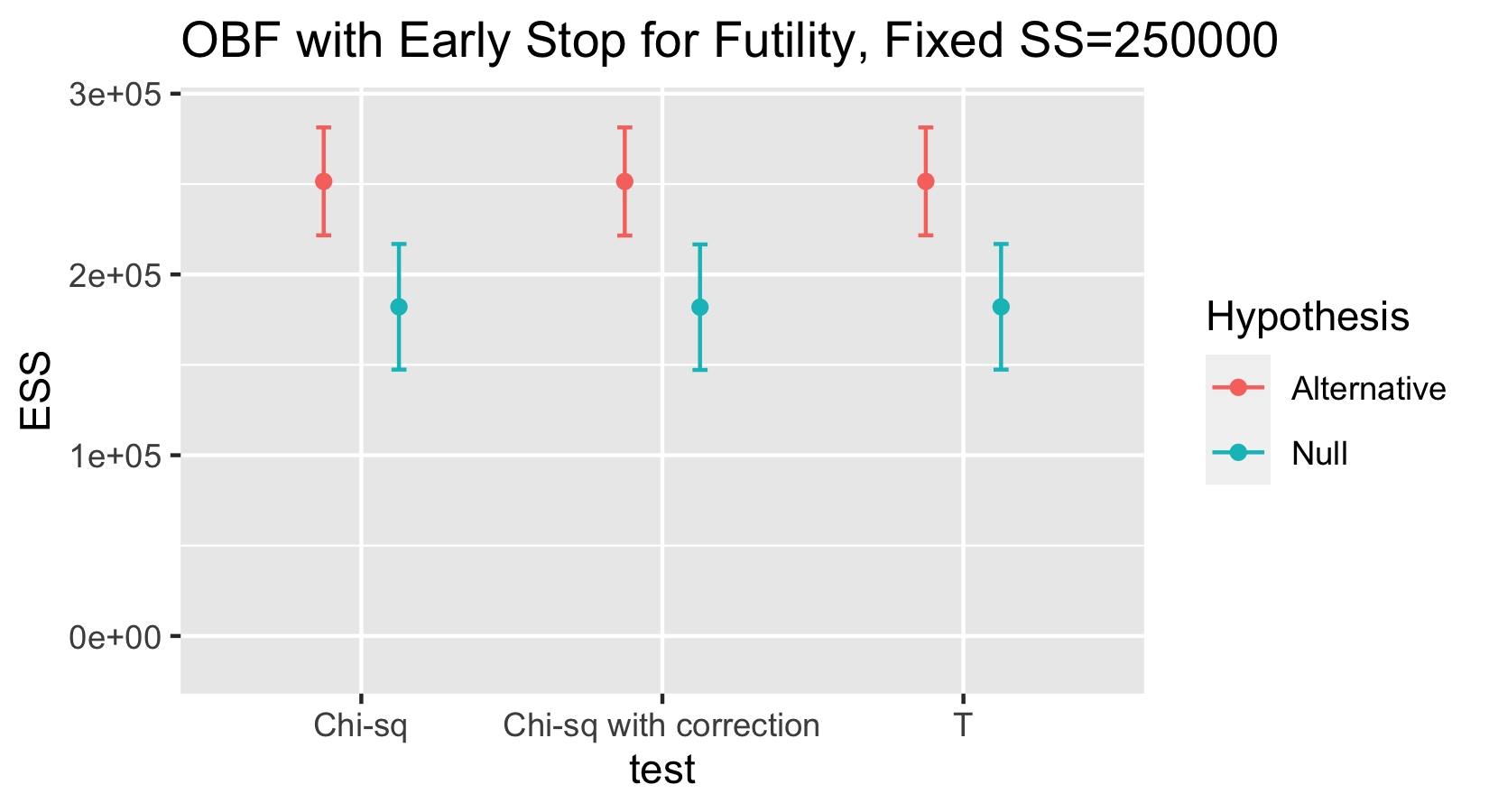

Supplement: Multimedia component 1 [file mmc1.docx]
